# An early-diverging iguanodontian (Dinosauria: Rhabdodontomorpha) from the Late Cretaceous of North America

**DOI:** 10.1371/journal.pone.0286042

**Published:** 2023-06-07

**Authors:** Lindsay E. Zanno, Terry A. Gates, Haviv M. Avrahami, Ryan T. Tucker, Peter J. Makovicky

**Affiliations:** 1 Paleontology, North Carolina Museum of Natural Sciences, Raleigh, North Carolina, United States of America; 2 Department of Biological Sciences, North Carolina State University, Raleigh, North Carolina, United States of America; 3 Department of Earth Sciences, Stellenbosch University, Stellenbosch, South Africa; 4 Department of Earth and Environmental Sciences, University of Minnesota, Minneapolis, Minnesota, United States of America; Chinese Academy of Sciences, CHINA

## Abstract

Intensifying macrovertebrate reconnaissance together with refined age-dating of mid-Cretaceous assemblages in recent decades is producing a more nuanced understanding of the impact of the Cretaceous Thermal Maximum on terrestrial ecosystems. Here we report discovery of a new early-diverging ornithopod, *Iani smithi* gen. et sp. nov., from the Cenomanian-age lower Mussentuchit Member, Cedar Mountain Formation of Utah, USA. The single known specimen of this species (NCSM 29373) includes a well-preserved, disarticulated skull, partial axial column, and portions of the appendicular skeleton. Apomorphic traits are concentrated on the frontal, squamosal, braincase, and premaxilla, including the presence of three premaxillary teeth. Phylogenetic analyses using parsimony and Bayesian inference posit *Iani* as a North American rhabdodontomorph based on the presence of enlarged, spatulate teeth bearing up to 12 secondary ridges, maxillary teeth lacking a primary ridge, a laterally depressed maxillary process of the jugal, and a posttemporal foramen restricted to the squamosal, among other features. Prior to this discovery, neornithischian paleobiodiversity in the Mussentuchit Member was based primarily on isolated teeth, with only the hadrosauroid *Eolambia caroljonesa* named from macrovertebrate remains. Documentation of a possible rhabdodontomorph in this assemblage, along with published reports of an as-of-yet undescribed thescelosaurid, and fragmentary remains of ankylosaurians and ceratopsians confirms a minimum of five, cohabiting neornithischian clades in earliest Late Cretaceous terrestrial ecosystems of North America. Due to poor preservation and exploration of Turonian–Santonian assemblages, the timing of rhabdodontomorph extirpation in the Western Interior Basin is, as of yet, unclear. However, *Iani* documents survival of all three major clades of Early Cretaceous neornithischians (Thescelosauridae, Rhabdodontomorpha, and Ankylopollexia) into the dawn of the Late Cretaceous of North America.

## Introduction

Refined spatiotemporal data on mid-Cretaceous biota is key to detangling the stepwise turnover and reassembly of terrestrial ecosystems leading up to a global temperature spike dubbed the Cretaceous Thermal Maximum (KTM) [1]. Paleobiodiversity data from the mid-Cretaceous of Western North America has long played a pivotal role in documenting patterns of mass extinction and subsequent diversification within the marine realm (e.g. [2]). However, tracing these patterns in terrestrial vertebrates has proven more challenging due to their lower relative preservation potential and collection biases that focus on more productive rock formations, for example, those of the intensely surveyed Campano-Maastricthtian (e.g., [3–5]). Such impediments notwithstanding, targeted explorations of early Late Cretaceous fossiliferous formations in the Western Interior Basin, North America are producing an increasingly rich and refined dataset that can be used to infer the impact of the KTM and corollary events, such as eurybatic changes [6], basin evolution [7], the “flowering” of landscapes and restructuring of Cretaceous forests [8, 9], and the aftermath of paleobiogeographic exchange (e.g., [10, 11]).

One of these fossil archives is the Cenomanian-age Mussentuchit Member of the Cedar Mountain Formation, central Utah, USA—a stratum known to entomb one of the most diverse early Late Cretaceous terrestrial assemblages globally [10], with nearly 100 species identified to date [12]. Historically, published palaeobiodiversity data from the Mussentuchit Member was almost exclusively based on species-rich and abundant microvertebrate bonebeds (e.g., [10, 13, 14]). Dinosaur-specific taxon tables were primarily generated from qualitative assays of isolated teeth, known to be reliable only at supraspecific taxonomic scales (see [15–17]). They included purported records of tyrannosaurids (cf. *Alectrosaurus* sp.); dromaeosaurine, velociraptorine, and troodontid paravians; therizinosaurs; a large-bodied indet. theropod; avialans; the tooth taxa cf. *Paranychodon* and cf. *Richardoestia*; and titanosauromorphs (cf. *Astrodon* sp.); as well as a diverse ornithischian assemblage characterized by thescelosaurids (denoted as “hypsilophodontids” and/or cf. *Zephyrosaurus* sp.), pachycephalosaurids, neoceratopsians, nodosaurids (cf. *Pawpawsaurus* sp.), hadrosaurids, and non-hadrosaurid iguanodontians (cf. *Tenontosaurus* sp.) [10, 12, 13, 18–20]. The discovery and study of macrovertebrate materials from the member resulted in increasing taxonomic specificity of some ornithischians such as refinement of “hadrosaurid indet.” to the hadrosauromorph *Eolambia caroljonesa* [21–23]; and nodosaurid indet. to *Animantarx ramaljonesi* [24]. Most recently, refinements have been made to the theropod fauna, including identification of the “indet. large-bodied theropod” as the allosauroid *Siats meekerorum* [25], and replacement of the tyrannosaurid cf. *Alectrosaurus* with the tyrannosauroid *Moros intrepidus* [11].

Here we describe a partial skeleton from the lower Mussentuchit Member, Cedar Mountain Formation, Utah representing the first record of an early-diverging ornithopod in the Late Cretaceous of North America and the first Late Cretaceous North American rhabdodontomorph documented to date. The identification of this new species permits refinement of previously identified records in the Mussentuchit dinosaur assemblage of indeterminate “iguanodontian” and cf. *Tenontosaurus*, and offers essential information on the evolutionary history, paleobiogeography, and morphological trends within Rhabdodontomorpha, an emerging, yet taxonomically unstable, clade of poorly known early-diverging ornithopods that current evidence suggests, may have had a more global distribution than historically recognized.

## Materials and methods

### Specimen

The specimen described in this study was collected from land managed by the Bureau of Land Management and reposited at the North Carolina Museum of Natural Sciences in Raleigh, NC, USA in 2014. All necessary permits were obtained for the described study, which complied with all relevant regulations.

### Terminology

We follow Nomina Anatomica Veterinaria [26] for anatomical terminology; Bell et al. [27] for dental terminology; Madzia et al. [28] for taxonomic definitions; the International Code of Phylogenetic Nomenclature (Phylocode), Version 4c [29] for taxonomic rules, and Cohen et al. [30] for chronostratigraphic age boundaries. For Fig 3, we scanned specimens with an Artec Space Spider high-resolution blue light scanner, post-processed them in Artec Studio 16 Professional, and manipulated scans in Blender version 3.3.1. Photographs were taken with an EOS 5D Mark II 21.1 megapixel full-frame CMOS digital single-lens reflex camera, processed with Adobe Photoshop 21.1.0, and layouts composed with Adobe Illustrator 24.1.

### Nomenclatural act

The electronic edition of this article conforms to the requirements of the amended International Code of Zoological Nomenclature, and hence the new names contained herein are available under that Code from the electronic edition of this article. This published work and the nomenclatural acts it contains have been registered in ZooBank, the online registration system for the ICZN. The ZooBank LSIDs (Life Science Identifiers) can be resolved and the associated information viewed through any standard web browser by appending the LSID to the prefix "http://zoobank.org/". The LSID for this publication is: urn:lsid:zoobank.org:pub:73607FBB-C153-4C4D-9BA4-453773F163D4. The electronic edition of this work was published in a journal with an ISSN, and has been archived and is available from the following digital repositories: PubMed Central, LOCKSS.

### Matrices

Hypotheses regarding the phylogenetic relationships of early-diverging (non-iguanodontian) neornithischian taxa are inconsistently resolved. We analyzed the evolutionary relationships of *Iani smithi* using maximum parsimony optimality and Bayesian inference with three recent phylogenetic matrices—Barta and Norell [31], Poole [32], and Dieudonné et al., [33]. These matrices include a representative sampling of early-diverging ornithopods, yet offer different taxonomic subsampling, morphological characterizations, homology statements, and best-supported primary tree topologies. Character matrices, a discussion of omitted characters, tree files, and result files are downloadable via MorphoBank Project 4556 http://morphobank.org/permalink/?P455

### Barta and Norell 2021

The Barta and Norell [31] matrix is a recent version of Madzia et al., [34], which largely derives from Boyd [35]. We updated character states for *Tenontosaurus tilletti* and *Te*. *dossi* based on first-hand observation of specimens (OMNH 58340, 34784, 34191, 10132, 16562, 08137; SMU 93B2, FWMSH 932B1; MOR 682, 2571). Changes to *Tenontosaurus* were substantial, between ~30–36% in both species, the majority (~16–22%) being previously unidentified (“?”) states. Key state modifications were made to dental traits of these taxa (e.g., documenting the presence of ridges confluent with denticles on the maxillary dentition and the presence of ridges on both sides of the dentary crowns). We further modified certain character states of other OTUs when our observations differed with existing assessments (e.g., *Zalmoxes*, *Gasparinisaura*, *Iguanodon*, *Camptosaurus*). Character 5 of Barta and Norell [31] only describes two states, yet codes for 3, therefore we changed OTUs coded as states 1 or 2, to state 1. We omitted characters 4, 6, 43, 46, 47, 50, 60, 67, and 111 because they were uninformative/duplicative, had unclear partitioning of states or had problematic homologies. We treated one character (char. 112) as additive following [31]. We used the composite Haya OTU. Note that in our Bayesian analysis, the latter character exceeded the required six-state maximum of MrBayes; therefore, character states 0 (six teeth) and 1 (five teeth) were combined into a single character state 0 (five/six teeth) since these traits occur in taxa outside Ornithopoda. *Marasuchus* was used as the outgroup in this analysis. We added the scores for *Transylvanosaurus* from Augustin et al., [36]; however, we changed the frontal participation in the orbit from more than to less than 25% (char. 63) because it is nearly excluded; and we changed char. 108 from 0 to? because the text states that the prootic and laterosphenoid sutures are indistinct [36].

#### Dieudonné et al., 2021

Dieudonné et al., [33] matrix is a more recent version of Dieudonné et al., [37] and combines characters formulated by Butler et al., [38] (modified by [39]), McDonald et al., [40], Brown et al., [41] and Boyd [35]. As before, we updated character states for the two species of *Tenontosaurus* based on personal observations and modified character states for taxa when our assessments differed from the authors. All character states modified from Dieudonné et al., [33] are noted in the archived data matrices. We omitted characters 62, 80, 83, 120, 228, and 243 because we found them to be uninformative, duplicative, or to have unclear partitioning of states or problematic homologies. Following [33], we treated characters 110,150, 159, and 203, as additive and used *Herrerasaurus* as the outgroup. We added the scores for *Transylvanosaurus* from [36] with the same modifications described for Barta and Norell [31] above.

#### Poole 2022

Poole [32] is the most recently published phylogeny to focus on early-diverging iguanodontians, comprised of a sample of traits encoded in existing matrices (184: 57%) and new characters (139: 43%). We recoded craniodental traits only for *Te*. *tilletti* and *Te*. *dossi* based on personal observations representing 11% and 18% character change for these taxa respectively, with a large portion being previously indeterminate states. We did not make modifications to the state codes of other taxa. We changed state (2) of character 120 to include premaxillary tooth number ranging from four–two in order to capture the state of *Iani*, and we changed character 113 to represent only the maximum number of ridges observed on maxillary teeth, removing polymorphisms. We omitted character 111 because of problematic homologies. We followed Poole [32] in considering the following characters additive: 22, 31, 48, 69, 70, 72, 81, 91, 96, 103, 105, 109, 120, 123, 124, 127, 129, 130, 136, 137, 150, 151, 153, 162, 172, 186, 200, 204, 205, 216, 218, 228, 248, 262, 263, 271, 278, 301, 321 and using *Eocursor* as the outgroup.

### Phylogenetic protocol

#### Parsimony

Phylogenetic analyses using equally weighted maximum parsimony were executed in TNT ver. 1.6 [42]. Trees were visualized and characters traced using Mesquite ver. 3.70 [43]. For all matrices, we conducted a new technology search with sectorial, ratchet, drift, and tree fuse under a driven search with 10 initial addseqs and 100 random seeds, finding min length 1,000 times. Ambiguous nodes were collapsed [44]. We recovered Most Parsimonious Trees (MPTs) with a minimum tree length of 901, 1,377, and 1,390 steps for the Barta and Norell [31], Dieudonné et al., [33], and Poole [32] matrices, respectively. MPTs from new technology searches of matrices were used for consensus estimates (strict and reduced consensus methods) and exhaustive heuristic searches for support metrics (Bremer support values [45] Maximum agreement subtrees [46] were calculated in TNT to identify unstable taxa and common topology among MPTs in each individual analysis. We mapped common synapomorphies in TNT for discussion.

#### Bayesian

Bayesian phylogenetic analyses were implemented in MrBayes ver. 3.2.7a [47]. Data matrices were divided into two anatomical partitions: 1) characters of the cranial and 2) postcranial skeleton, with each partition unlinked to allow independent character evolution between these anatomical regions [48, 49]. We partitioned characters by anatomical region, instead of the automatically applied partitioning by number of character states, for several reasons. First, the number of states in a character is arbitrarily set by the researcher and does not necessarily have any biological or evolutionary significance. Second, the arbitrary selection of character states does not necessarily equate to equivalent rates of evolution between character states (i.e., the amount of change between hypothetical character states 0 and 1 can be more or less than the amount of change between states 1 and 2 in the same character). Finally, dividing the data into only two partitions allows for enough data to be present in each partition, unlike the more dispersed data in up to six partitions (one character in the Barta and Norell [31] matrix has six character states). In non-tip-dated analyses, the Mk-parsinf model [47, 50, 51] was not implemented, but since all parsimony non-informative characters were removed prior to the analysis, running the Mkv model with log-normal character rate variation mimics this model. Runs consisted of 20 million generations for the Barta and Norell [31] and Poole [32] matrices and 25 million for the Dieudonné et al., [33] matrix, utilizing six chains with three swaps on a 4-core Mac Pro. For both analyses, we implemented a 25% burn-in, and sampling every 4,000 generations. The split frequency of the final 1,000 samples from the Dieudonné et al., [33] and Poole [32] non-calibrated time matrices were 0.008 and 0.009, respectively.

### Paleohistology

To measure enamel thickness, we molded and cast one dentary tooth from NCSM 29373 (NCSM field number MM14-FS10) prior to sampling. The tooth was then embedded in a clear epoxy resin (EPO-TEK 301), cut along the labiolingual plane with a Buehler IsoMet 1000 Precision Saw, and polished on one side with a Buehler MetaServ 250 Grinder Polisher using a series of abrasive paper disks with increasing grit sizes (400–1200). The polished blocks were mounted on frosted glass slides with epoxy and ground to desired thickness. Thin-sections were observed and images were captured with a Keyence VHX-7000 digital microscope with an FI (VHX-7100) head with a polarizer, and custom-built lambda filter. We used ImageJ (ver. 1.53a) to measure enamel thickness.

### Institutional abbreviations

**CM**, Carnegie Museum, Pittsburgh, PA, USA; **FWMSH**, Fort Worth Museum of Science and History, Fort Worth, TX, USA; **LPB (FGGUB)**, Laboratory of Paleontology, Faculty of Geology and Geophysics, University of Bucharest, Bucharest, Romania; **MC**, Musée de Cruzy, France; **MDS**, Dinosaur Museum of Salas de los Infantes, Burgos, Spain; **MOR**, Museum of the Rockies, Bozeman, MT, USA; **NCSM**, North Carolina Museum of Natural Sciences, Raleigh, NC, USA; **NHMUK R**, Natural History Museum, London, U.K.; **NMV P**, Museum Victoria, Melbourne, Victoria, Australia; **OMNH**, Oklahoma Museum of Natural History, Norman, OK, USA; **PIUW**, Paläontologisches Institut, University of Vienna, Vienna, Austria; **SMU**, Southern Methodist University, Dallas, TX, USA; **UBB**, Universitatea din Babes-Bolyai, Cluj-Napoca, Romania; **YPM**, Yale Peabody Museum, New Haven, Connecticut, USA.

## Results

### Systematic palaeontology

Dinosauria Owen, 1842 [52]

Ornithischia Seeley, 1888 [53]

Ornithopoda Marsh, 1881 [54]

Iguanodontia Baur, 1891 [55]

Rhabdodontomorpha [37]

Heterodefinitional junior synonym. Rhabdodontoidea [32]

### Remarks

Madzia et al., [28] provided a stem-based definition of Rhabdodontomorpha, converting the node-based definition provided by Dieudonné et al., [37]. Under the phylogenetic topologies recovered here (and most recent published topologies e.g., [34, 36, 37, 39, 61], the definition provided by Poole [32] for Rhabdodontoidea—a stem-based taxon including all taxa more closely related to *Zalmoxes robustus* and *Rhabdodon priscus* than to *Dryosaurus altus*—is a heterodefinitional junior synonym of the clade Rhabdodontomorpha defined by Madzia et al., [28]—the maximum clade containing *Rhabdodon priscus* (Matheron [56]), but not *Hypsilophodon foxii* (Huxley [57]) and *Iguanodon bernissartensis* (Boulenger [58]).

*Iani* n. gen.

urn:lsid:zoobank.org:act:75BB88A7-DE02-48F2-9BFC-B8AF913EC4E

#### Etymology

*Iani* (Latin)(/YAN-ee/), of Ianus, as stemming from the Roman god who presides over transitions and in reference to the changing biota of the mid-Cretaceous of western North America.

*Iani smithi* sp. nov.

urn:lsid:zoobank.org:act:11117CEF-49C9-4B26-A409-039D635A6095

#### Diagnosis

As for the type species (see below).

#### Holotype

NCSM 29373, a partial skeleton of a skeletally immature individual.

#### Etymology

The species nomen honors Joshua Aaron Smith for his contributions to the discovery and conservation of paleontological resources in the region, particularly early explorations by the NCMNS.

#### Locality and horizon

**Fig 1:**
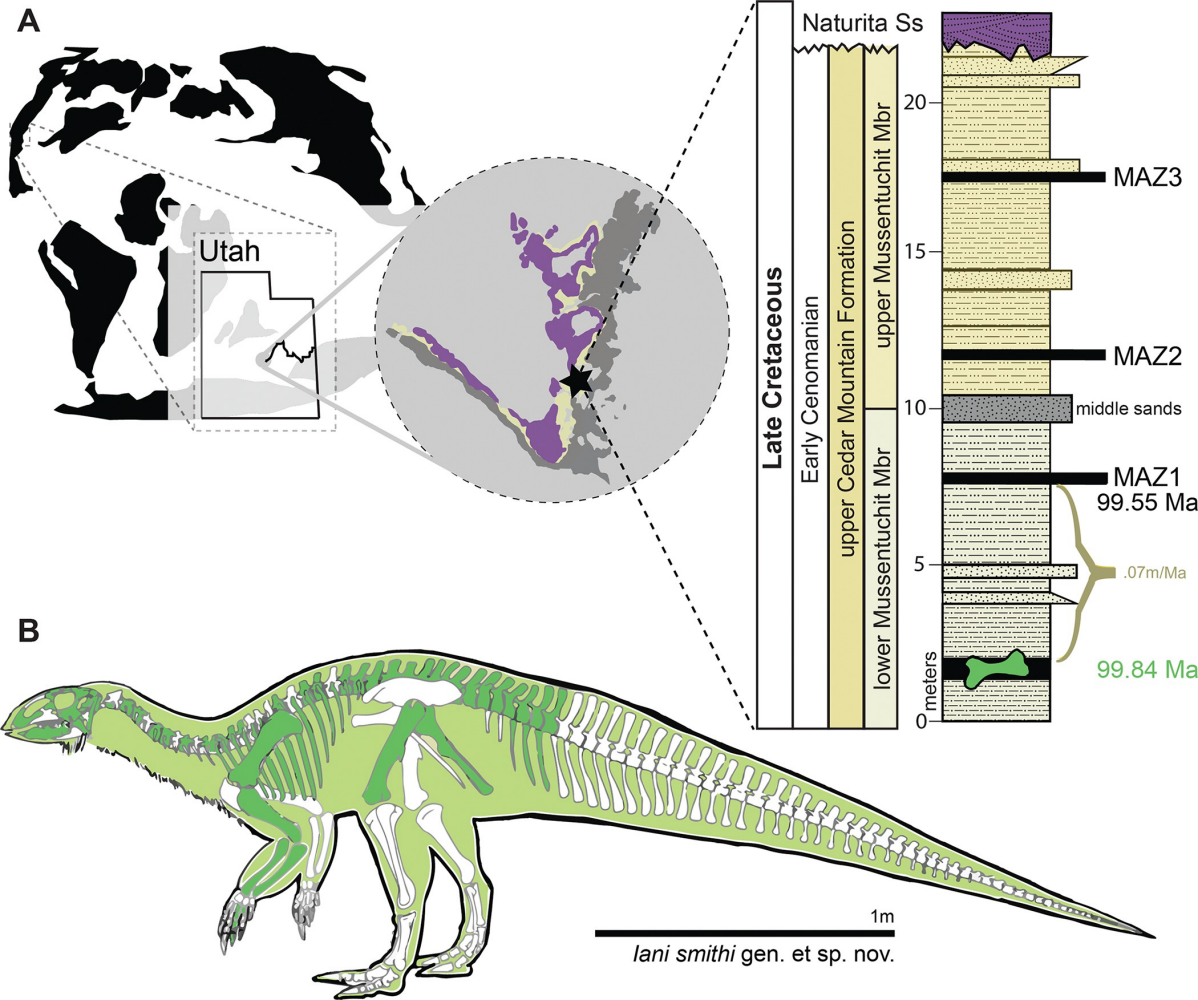
Location of holotype locality for *Iani smithi* (NCSM 29373). (A) Global map showing location of Mussentuchit Member outcrop in central Utah, western North America, and a stratigraphic section at the quarry with dated ash horizons; and (B) graphical representation of preserved skeletal elements of the holotype specimen (NCSM 29373). Preserved elements are colored on the left facing skeletal whether they derive from the right or left side of the body. Exact positions of chevrons and ribs unknown due to poor preservation. See text and figures for specific positioning and completeness of elements. Abbreviations: MAZ1–4, Mussentuchit Ash Zones 1–4 [59]. Outcrop extent adapted from USGS National Map Viewer (public domain): http://viewer.nationalmap.gov/viewer/. Scale bar 1 m.

NCSM 29373 was recovered from the lower Mussentuchit Member, upper Cedar Mountain Formation, Emery County, Utah, USA (“Fortunate Son” NCPALEOUT14; Fig 1) approximately 1.0 m above the contact with the underlying Ruby Ranch Member and ±7.0 m below MAZ1 (99.490 +0.057/-0.050 Ma) [59]. A Bayesian depositional age estimation and age-depth modelling yielded an estimated age for emplacement for NCSM 29373 that is no later than 99.652 +0.413/-0.094 Ma [59: Figs 3 and 4]. Skeletal elements of a single individual were found disarticulated and scattered across an area of approximately four-square meters. *Iani* was the only taxon represented in the quarry.

#### Diagnosis

**Fig 2:**
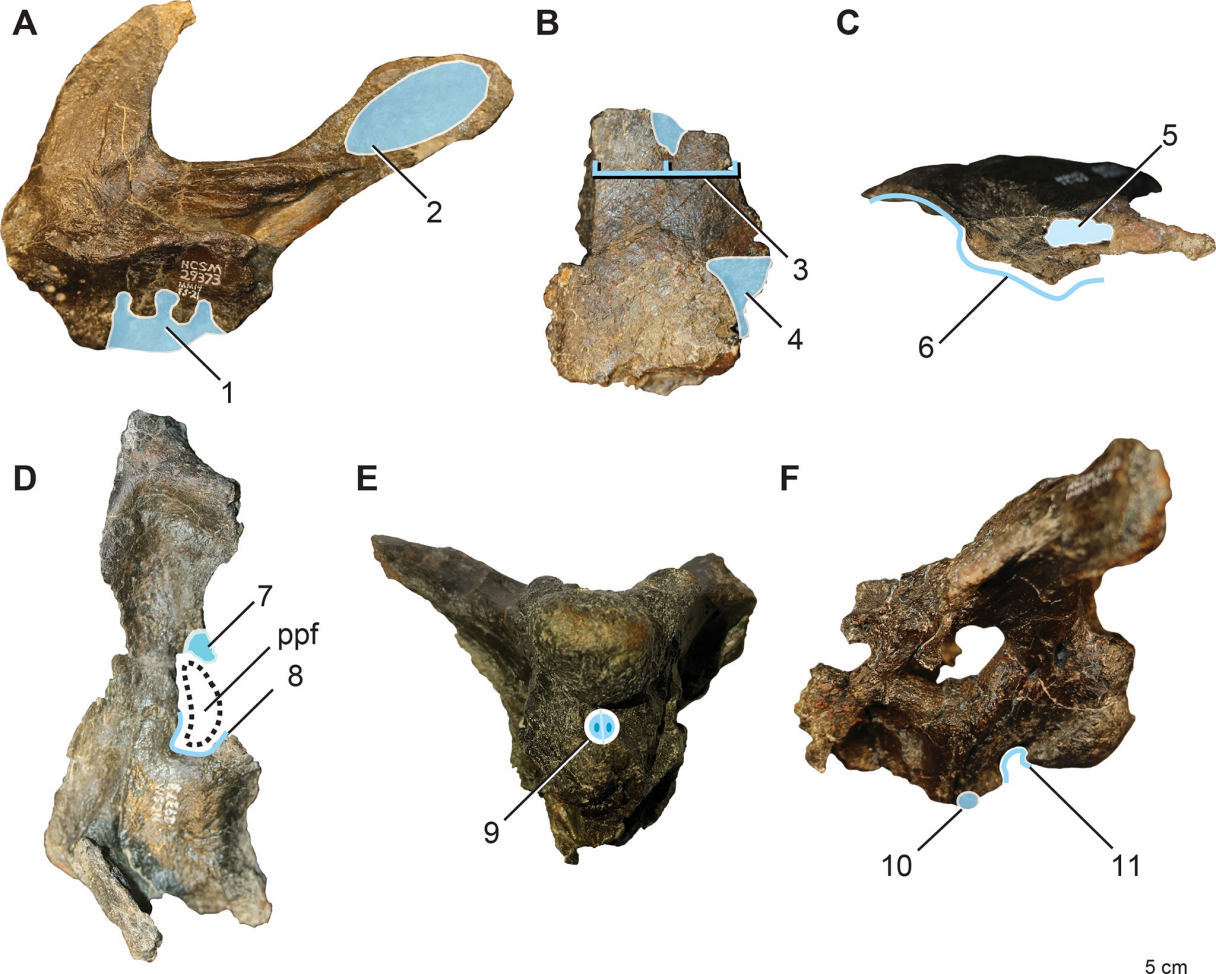
Diagnostic features of *Iani smithi* (NCSM 29373). (A) right premaxilla in medial view; (B) right frontal in dorsal view; (C) left squamosal in caudal view; (D) right palatine, ectopterygoid, and pterygoid in dorsal view (see Fig 9); braincase in (E)ventral and (F) left lateral views. Numbered traits (1–11) correspond to taxon diagnosis. Abbreviations: ppf, postpalatine foramen. Elements not to scale.

Skeletally immature non-dryomorphan ornithopod differentiated by the following combination of characters (autapomorphies denoted with asterisk): three premaxillary teeth* (Fig 2A1); distinct oval fossa on the caudomedial aspect of premaxillary lateral process* (Fig 2A2); caudalmost margin of prefrontal facet on frontal tapering and centered between interfrontal suture and orbital rim (shared with *Convolosauru*s) (Fig 2B3); hatchet shaped postorbital facet on frontal with rostrally convex caudal and straight rostral margins* (Fig 2B4); posttemporal foramen housed entirely in squamosal (shared with *Zalmoxes*) (Fig 2C5); robust tab extending from the caudal aspect of the squamosal into concavity on the paroccipital process near its base (shared with *Te*. *tilletti*)(Fig 2C6); distinct, triangular caudomedial prong projecting off the caudal aspect of the palatine that, along with the maxilla, forms part of the lateral margin of the postpalatine foramen* (Fig 2D7); distinct u-shaped notch in ectopterygoid between the palatine and jugal process forming part of the caudal, medial, and lateral margins of the postpalatine (suborbital) foramen* (Fig 2D8); basioccipital with sharp midline lamina bordered contralaterally by two basioccipital foramina housed entirely within a depressed fossa on the rostroventral surface* (Fig 2E9); caudally projecting conical tubercle on midline of rostroventralmost tip of basioccipital* (Fig 2F10); angular occipital condyle on basioccipital with flattened ventral and caudal aspects, and a sharp, rostrally projecting lip, which together with the caudally projecting tubercle on the rostral basioccipital forms a nearly enclosed, fish-hook shaped profile of the caudoventral perimeter in lateral view* (Fig 2F11).

#### Description and comparisons

NCSM 29373 is a skeletally immature individual represented by a largely complete, disarticulated skull; cervical, dorsal, sacral, and caudal vertebrae; associated ribs and haemal arches; and portions of the right and left pectoral girdle, left pelvic girdle, right forelimb, and right hindlimb. Elements of the braincase (except the supraoccipital) are in an early state of fusion; however, much of the axial column remains unfused. Standard skeletal measurements are provided in Table 1. A three-dimensional rendering of the skull reconstruction in 360° is downloadable via MorphoBank Project 4556 http://morphobank.org/permalink/?P455

**Table 1:** Skeletal measurements of *Iani smithi* (NCSM 29373).

Element	Measurement	Value (mm)
Femur (right)	Proximodistal length of element	360
Femur (right)	Circumference proximal to fourth trochanter	169
Femur (right)	Circumference distal to fourth trochanter	156
Ischium (left)	Proximodistal length as preserved	303
Ischium (left)	Circumference distal to obturator process	77
Ischium (left)	Expansion of distal boot as preserved	40
Ulna (left)	Proximodistal length of element	240
Ulna (left)	Craniocaudal length of ulnar head	59
Ulan (left)	Mediolateral length of distal end	40
Ulna (left)	Minimum circumference of shaft (mid length)	75
Radius	Proximodistal length as preserved of longest segment	143
Humerus (right)	Proximodistal length of proximal segment	165
Humerus (right)	Proximodistal length of distal segment	115
Humerus (right)	*Minimum proximodistal length	*285
Humerus (right)	Craniocaudal length of humeral head	87
Humerus (right)	Circumference of shaft along dorsal margin of distal segment (approximately minimal shaft girth)	115
Scapula (right)	Length of scapular head between acromion process and ventral corner of glenoid	84
Scapula (left)	Length of scapular head between acromion process and ventral corner of glenoid	110
Scapula (right)	Minimum width across scapular neck	53
Scapula (left)	Minimum width across scapular neck	55
Scapula (right)	Length from junction of acromion and corocoid to midpoint along distal-most blade	280
Scapula (right)	Length from ventral glenoid corner to tip of distal blade following contour of scapula	260
Dentary (right)	Rostrocaudal length complete measuring parallel to base of dentary	145
Dentary (left)	Rostrocaudal length complete measuring parallel to base of dentary	144
Dentary (right)	Height of coronoid process measured perpendicular to the root of the proximal-most tooth	23.3
Dentary (left)	Height of coronoid process measured perpendicular to the root of the proximal-most tooth	23.4
Dentary (right)	Length of diastema measured from the rostral end of the dentary to the rostral end of first tooth	26.4
Dentary (left)	Length of diastema measured from the rostral end of the dentary to the rostral end of first tooth	30.8
Dentary (right)	Dorsoventral height of dentary at middle of tooth row	31.4
Dentary (left)	Dorsoventral height of dentary at middle of tooth row	33.2
Dentary (right)	Rostrocaudal length of tooth row	107.4
Dentary (left)	Rostrocaudal length of tooth row	105.0

#### Skull

**Fig 3:**
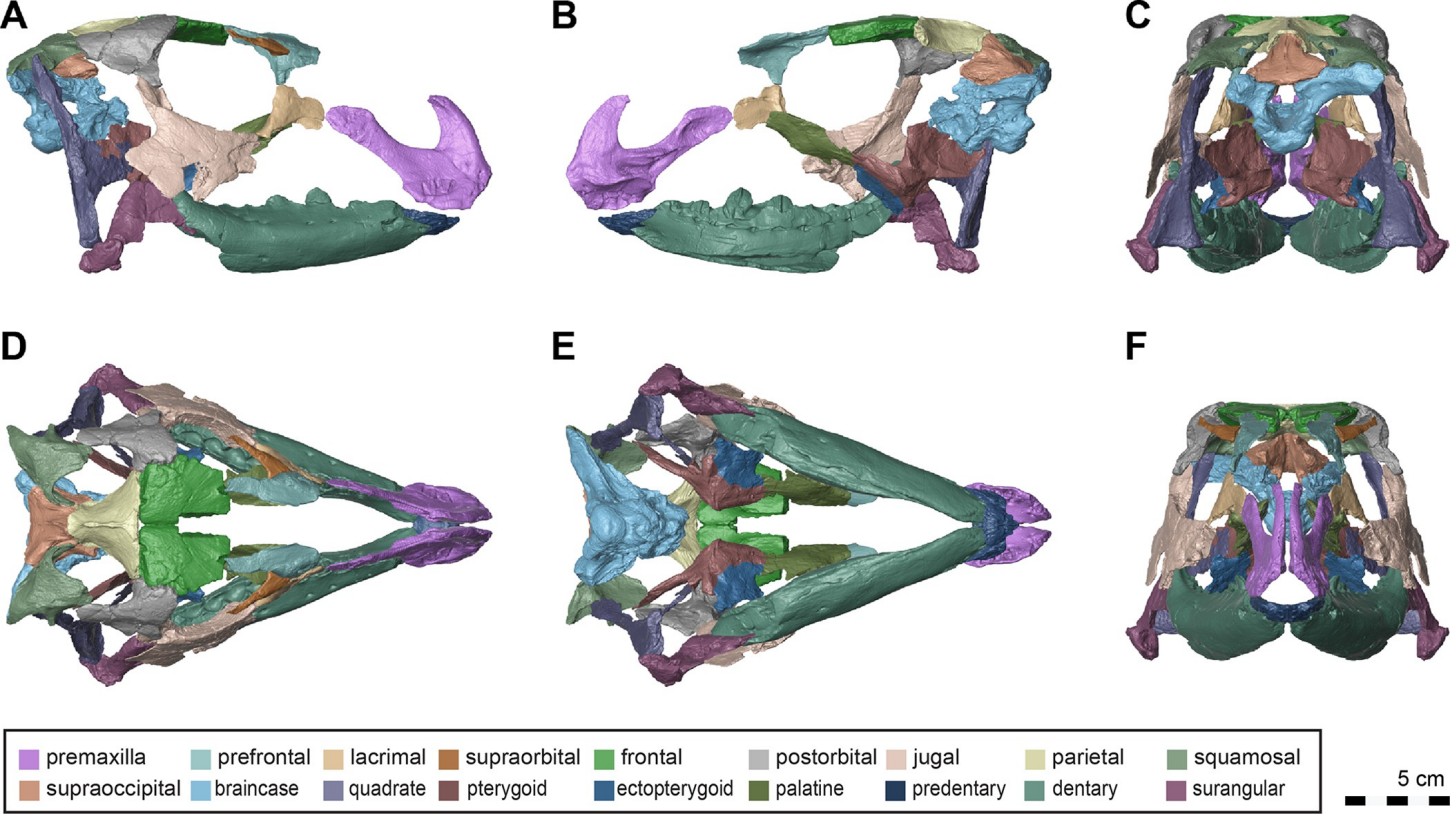
Three-dimensional reconstruction of the skull of *Iani smithi* (NCSM 29373). (A) right lateral and (B) medial views of skull with left facial bones removed; skull reconstruction with all preserved elements (some mirrored) in (C) caudal, (D) dorsal, (E) ventral, and (F) rostral views. Scale bar 5 cm.

The majority of the skull is preserved. However the nasal and maxilla are among the missing elements; therefore, we have estimated facial length from mandibular length (Fig 3). Although incomplete, the naris of *Iani* was suboval and likely rostrocaudally longer than dorsoventrally tall as in *Tenontosaurus* (*Te*.), and pointed rostrally (Fig 3). *Iani* also exhibits a large, subrectangular orbit with an angular rostroventral margin; the latter is shared with *Te*. *tilletti* [60] (and less so *Z*. *robustus* [61]). The supratemporal fenestra is slightly deformed, yet rostrocaudally longer than mediolaterally wide and its long axis is oriented rostrolaterally/caudomedially. The infratemporal fenestra is rostrocaudally wide and possesses a sharp, 90° rostroventral corner. We estimate the floor of the infratemporal fenestra to be slightly lower than the orbit.

#### Premaxilla

**Fig 4:**
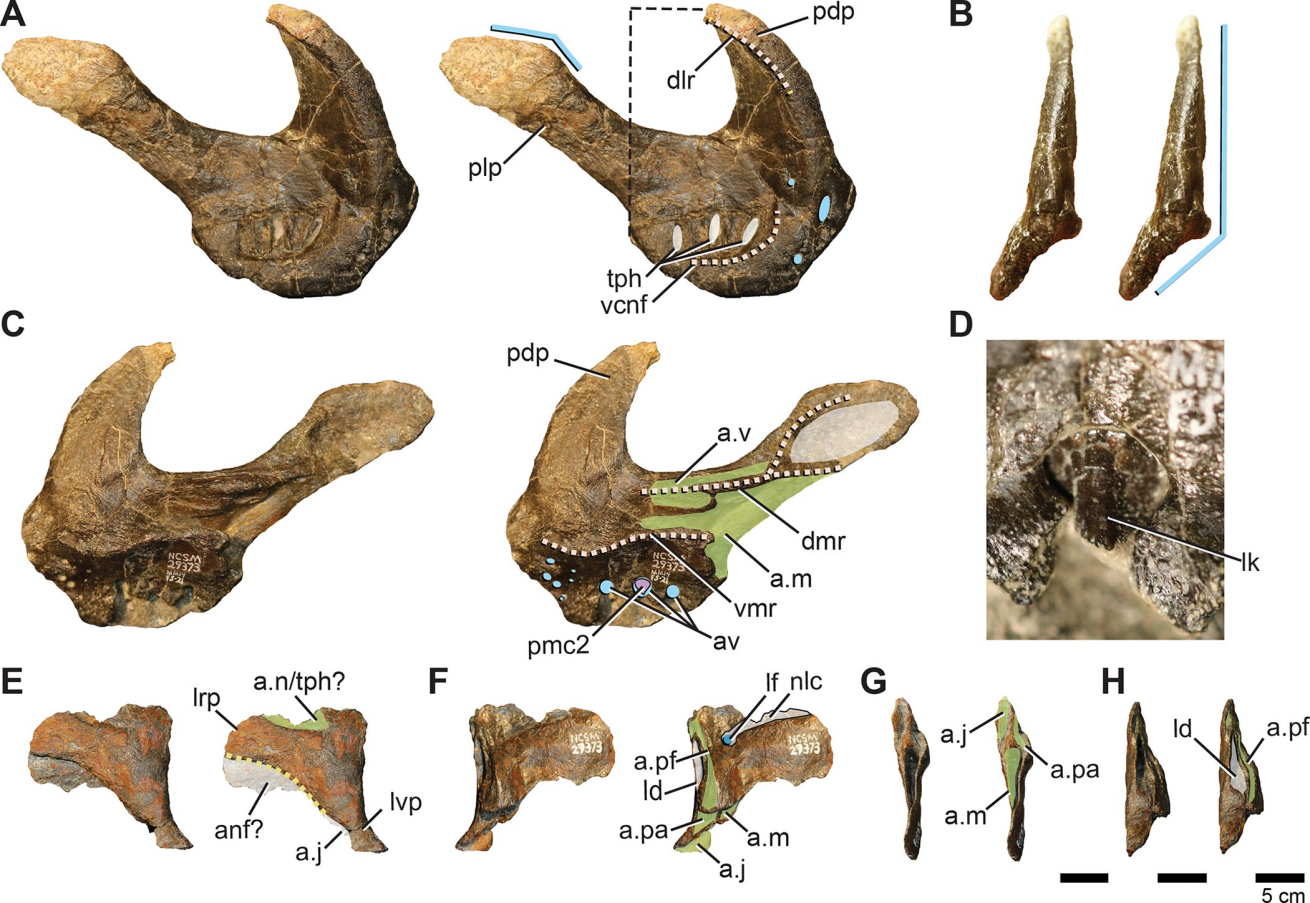
Premaxilla, premaxillary dentition, and lacrimal of *Iani smithi* (NCSM 29373). Right premaxilla in (A) lateral, (B) rostral, (C) medial views; premaxillary tooth in (D) lingual view; left lacrimal in (E) lateral, (F) medial, (G) rostral, and (H) caudal views. Abbreviations: a.j, jugal articulation; a.m, maxilla articulation; a.n, nasal articulation; anf, antorbital fossa; a.pa, palatine articulation; a.pf, prefrontal articulation; a.v, vomer articulation; av, alveoli; dlr, dorsal lateral ridge; dmr, dorsal medial ridge; ld, lateral duct; lf, lacrimal foramen; lk, lingual keel; lrp, lacrimal, rostral process; lvp, lacrimal, ventral process; nlc, nasolacrimal canal; pdp, premaxilla, dorsal process; plp, premaxilla, lateral process; pc2, premaxilla, second tooth crown; tph, taphonomic artifact; vcnf, ventral circumnarial fossa; vmr, ventral medial ridge. Color annotation: white, depressions/fossae/grooves; green, articular surfaces; blue circles, foramina; light blue lines, marginal contours; peach dashed lines, ridges/internal contours; light purple, dentition. Scale bar 5 cm. (D) not to scale.

A nearly complete right premaxilla (missing only the rostral-most extent of the body and perhaps a fraction of the dorsal process) (Fig 4A) and fragments of the left premaxilla are preserved. The premaxilla exhibits the general ornithopod condition of a broad, slightly transversely expanded, rostrum (Fig 4B). The edentulous portion of the oral margin is pierced by numerous foramina (Fig 4A and 4C). Caudally, the oral rim thickens and is disrupted by alveoli for three premaxillary teeth (Fig 4C). Ornithopods retaining premaxillary dentition are rare. *Te*. *dossi* retains at least one premaxillary tooth [62], *Talenkauen* retains two [63], and *Convolosaurus* has four premaxillary teeth [64]. An unnumbered premaxillary fragment of an indeterminate rhabdodontomorph is reported to contain three tooth roots; however, it is unclear if this was the full complement of teeth [37]. The rostrolateral margin of the premaxilla is positioned only about 45 degrees from the vertical (Fig 4B), similar to *Convolosaurus* (SMU 72316, SMU 72834) and *Rhabdodon* [65]. This differs from the derived condition of hadrosaurids in which the same region is nearly horizontal, and the intermediate condition of *Te*. *tilletti* (OMNH 34191) [60].

**Fig 5:**
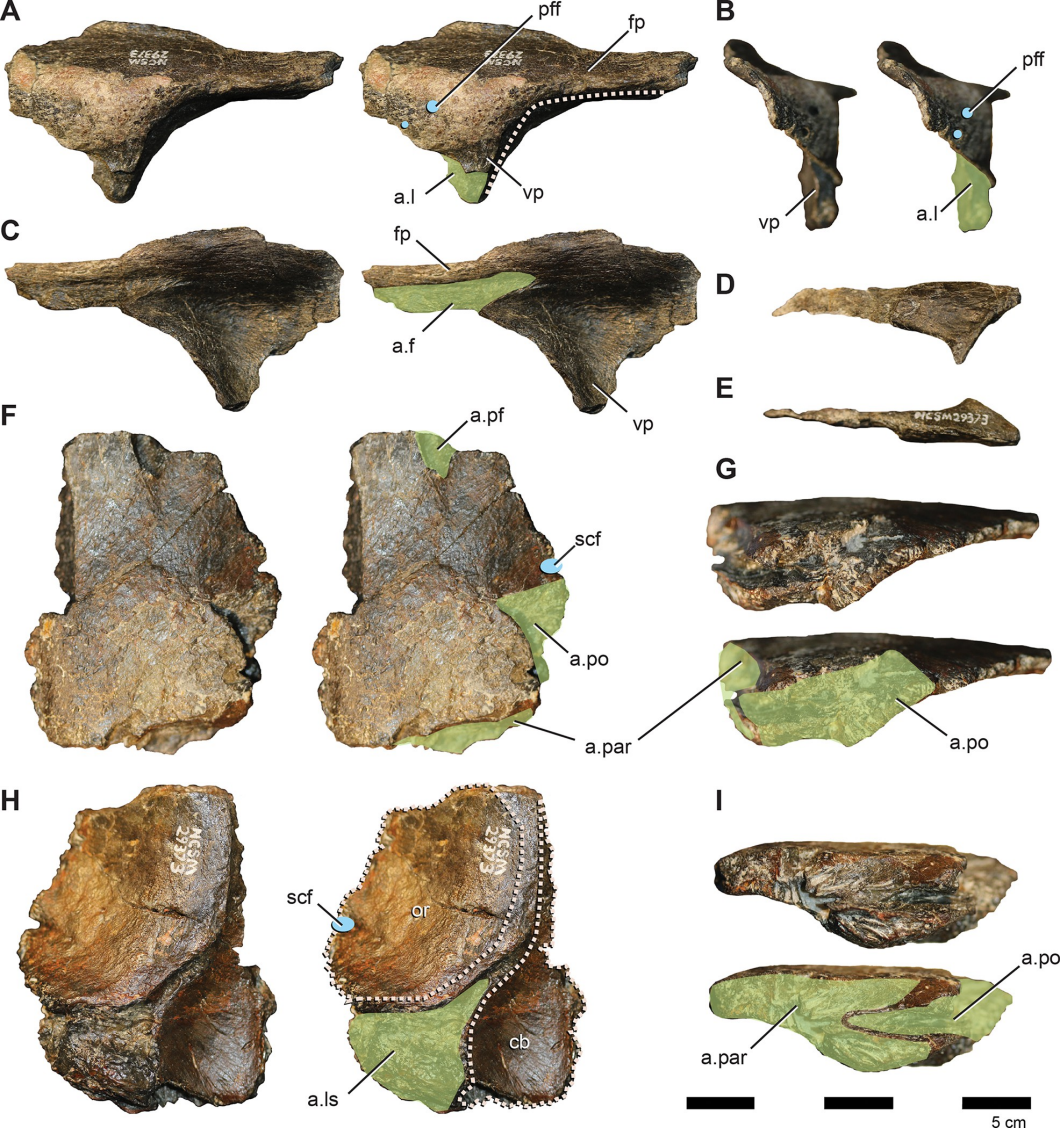
Prefrontal, frontal, and supraorbital of *Iani smithi* (NCSM 29373). Left prefrontal in (A) lateral, (B) rostral, (C) medial views; right supraorbital in (D) lateral and (E) dorsal views; right frontal in (F) dorsal, (G) lateral, (H) ventral, and (I) caudal views. Abbreviations: a.f, frontal articulation; a.l, lacrimal articulation; a.pf, prefrontal articulation; a.ls, laterosphenoid articulation; a.par, parietal articulation; a.po, postorbital articulation; cb, depression for the cerebral bulb; or, orbit; pff, prefrontal foramen; scf, supraciliary foramen. Color annotation: white, depressions/fossae/grooves; green, articular surfaces; blue circles, foramina; light blue lines, marginal contours; peach dashed lines, ridges/internal contours. Scale bar 5 cm.

In lateral view, the dorsal process is rostrocaudally wide and rapidly tapers, terminating prior to or maximally at the caudalmost aspect of the premaxillary oral margin (Fig 4A) as in *Rhabdodon* [65], as opposed to the elongate, gently tapering dorsal process of *Te*. *tilletti* [60], which extends caudally as far as the lateral process [60: Fig 5]. The dorsal process is subtriangular in cross-section due to a pronounced lateral ridge that grades rostrally into a rounded rostral margin of the premaxillary body (Fig 4A), a feature also observed on *Rhabdodon* [65]. The lateral process is much longer than its dorsal counterpart as in *Rhabdodon* [65]. In lateral view, the lateral process is rostrally constricted and bears a dorsal peak at 65% of its length before sharply tapering to the caudal termination (Fig 4A and 4C). This mid-process dorsal peak is similar to that of *Camptosaurus dispar*, *Dryosaurus altus* [66], and *Rhabdodon* [65]. *Te*. *dossi* and *Te*. *tilletti*, on the other hand, exhibit a rostrally constricted and dorsoventrally expanded blunt end to the lateral process (FWMSH 93B2) [60], lacking a pronounced dorsal step. A weakly demarcated ridge runs from the tip of the dorsal process around the expanded rostral portion of the premaxilla to terminate just caudal to the premaxillary teeth. This marks the rostral periphery of the circumnarial fossa (Fig 4A). Dorsal to this, are three depressions corresponding to the three premaxillary alveoli and resulting from taphonomic crushing (Fig 4A).

Medially, the most prominent features are two horizontal shelves that underlie the rostral narial fenestra and encapsulate the articulation with the rostral maxilla (Fig 4C). The ventral shelf is a mediolaterally broad plate roofing the rostral oral cavity that would have ventrally buttressed the rostral process of the maxilla. Some crushing has forced the floor of this shelf to curl dorsally. The dorsal shelf is mediolaterally more reduced and dorsoventrally thicker than its ventral partner. There is a deep subcircular fossa, likely marking where the lateral process on the rostrodorsal part of the maxilla inserted (Fig 4C), as in *Te*. *tilletti* [65]. Dorsally, it forms the ventral rim of a horizontally inclined articular surface that likely held the vomer (Fig 4C) as in *Z*. *robustus* [61] and *Te*. *tilletti* [60]. Ventral to the dorsal ridge there is an elongate, striated depression that likely marks a secondary contact with the vomer (dorsal and ventral to the ridge) as in *Te*. *tilletti* [60: Fig 10].

Caudal to this, the dorsal shelf bifurcates onto the lateral process of the premaxilla, becoming a thin ridge (Fig 4C). The ventral branch divided the articulation with the rostrodorsal maxilla from a pronounced fossa embossed on the caudomedial region of the premaxillary lateral process (Figs 2A and 4C). A thin ventral ridge is present on *Rhabdodon* [65]. The dorsal branch rises caudodorsally following the lateral process to form the dorsal border of the medial fossa.

#### Maxilla

No maxilla is preserved with *Iani*, nonetheless based on the articulation facet on the premaxilla, it is possible to ascertain that the anterior region possessed a straight, elongate rostral process

#### Lacrimal

Left and right lacrimals are preserved; however, the right element is mediolaterally crushed. The lacrimal is subtriangular, as opposed to the subrectangular lacrimal of *Te*. *tilletti* [60]. In dorsal view, it bears a transversely thickened orbital margin that rapidly tapers rostrally to a mediolaterally thin rostral process.

In lateral view, there is a slight depression on the rostrodorsal lacrimal that may signify overlap of the nasal (Fig 4E) as in *Te*. *tilletti* and *Thescelosaurus* (*Th*.) *neglectus* [60, 67], although due to damage it is difficult to interpret on *Iani* and it may be a taphonomic artifact. Weishampel et al., [61] report that on *Z*. *robustus*, the lacrimal and prefrontal form an interlocking scarf joint, where the prefrontal laterally overlapped the lacrimal rostrally, and the lacrimal overlapped the prefrontal caudally. This cannot be evaluated on *Iani*, and we consider this scar more likely to represent overlap of the nasal; however, it is clear that caudal to this region the lacrimal overlapped the prefrontal ventral ramus medially (Fig 4F).

On the ventral margin of the rostral process, a thin flange of bone extends from the medial aspect creating the medial wall of a deep, tear-drop shaped sulcus along the ventral margin that widens caudally and is rimmed laterally by a sharp ridge (Fig 4E). This medial wall originates at the junction of the rostral and ventral processes and becomes dorsoventrally more extensive rostrally (Fig 4E). A bony flange in this region forms the medial lamina of the antorbital fossa in *Lesothosaurus* and *Hypsilophodon* [68–70] and *Te*. *dossi* (FWMSH 93B1). In *Te*. *tilletti*, the medial lamina is not exposed in lateral view; however, a deep, ventrally open pocket formed by medial and lateral walls does comprise the dorsal margin of the antorbital fenestra [60]. On *Iani* we interpret this feature as indicative of an external antorbital fenestra and foramen, consistent with the morphology of *Te*. *dossi*.

*Zalmoxes robustus* [61] appears to lack a defined ventral process. In contrast, the ventral ramus of *Iani* is elongate and angles caudally as it approaches articulation with the jugal (Fig 3), forming an angular rostroventral margin of the orbit as in *Te*. *tilletti* [60]; however in the case of *Iani*, the lacrimal also contributes slightly to the ventral rim of the orbit. The jugal articulates with the lacrimal via a caudomedially oriented, oblique sulcus visible in medial and caudal views (Fig 4F and 4G). An additional sulcus excavates the ventral process in medial view and is walled off from the jugal and maxillary sulci. This likely represents an articulation point with the palatine, although a maxillary contact here cannot be ruled out (Fig 4F and 4G).

In caudal view, there are lateral and medial sulci along the orbital margin. The lateral sulcus is the lacrimal duct. It passes through the orbital margin and reappears as a groove (nasolacrimal canal, Fig 4F) on the medial surface of the rostral process as in *Hypsilophodon* [68, 69] and *Z*. *robustus* [61]. As in *Z*. *robustus* and *Te*. *tilletti*, the medial face of the lacrimal is depressed ventral to the nasolacrimal canal [60, 61]. We suggest that the medial sulcus likely marks the articulation point with the prefrontal (Fig 4F), which would have been ventrally extensive as in *Te*. *tilletti* [60], rimming the entire orbital margin caudomedially and touching the palatine ventrally. A groove is noted in this area on *Hypsilophodon*, filled by a “slender rod” of bone [69, pg. 34], and may represent an elongated ventral process of the prefrontal on that taxon.

#### Prefrontal

A complete left prefrontal is preserved (Fig 5A–5C). It is substantial in length, estimated to be three-quarters the length of the frontal. Similar frontal/prefrontal proportions are present in *Te*. *tilletti* (~70%, OMNH 58340), *Th*. *neglectus* (67%, NCSM 15728), and *Convolosaurus* (75–80%, SMU 74678, 72834). In contrast, *Haya* [31] has a prefrontal that is ~50% or less the length of the frontal. In dorsal view, the caudal aspect is mediolaterally widest, as in neornithischians generally. In general form, the prefrontal of NCSM 29373 is similar to *Convolosaurus* [64] in possessing a rostrally extensive lamina connecting the rostral and ventral processes in lateral view (Fig 5A), in contrast to the more “T-shaped” morphology of *Te*. *tilletti* [60]. *Iani* also possesses a long, straight, mediolaterally narrow, and caudally tapering frontal process (Fig 5A) that is cradled both medially and laterally by the frontal as in *Convolosaurus* [64]. This contrasts with the shorter, transversely broader, and caudally rounded frontal process of *Te*. *tilletti* and *Z*. *shqiperorum* (UBB NVZ1-38) [61]. In lateral view, the orbital margin is angular (Fig 5A) as opposed to gently curved, and creates an oblique rostrodorsal corner to the orbit as in *Te*. *tilletti* (OMNH 58340) [60]. Judging by the extent of the articular facet on the lacrimal, the ventral process appears to have been elongate, extending nearly to the contact with the jugal in caudomedial view as in *Te*. *tilletti* [60], and in contrast with thescelosaurids (e.g., *Haya* [31]; *Th*. *neglectus* [67]). When articulated, the prefrontal and frontal yield a straight dorsal margin of the orbit. The prefrontal of *Iani* lacks the lateral tubercle present on *Te*. *tilletti*, and there is less overlap between the lacrimal and prefrontal both medially and laterally than observed on *Te*. *tilletti* [60]. A foramen is centrally positioned between the rostral and ventral processes (craniocaudally) and faces laterally (Fig 5A and 5B). Such a foramen is absent on *Tenontosaurus* [60] (FWMSH 93B1), but present in *Haya* [71] and was previously considered autapomorphic for *Th*. *neglectus* [72]. However, in thescelosaurids, the foramen is located directly dorsal to the ventral process and faces dorsally.

#### Supraorbital (palpebral)

A single right supraorbital is preserved among the elements of the skull (Fig 5D and 5E). It is free from the orbit and traverses less than three-quarters of the rostrocaudal width as in some thescelosaurids (e.g., *Zephyrosaurus*, *Orodromeus* [73]), *Dysalotosaurus* [74], and *Hypsilophodon* [69], and in contrast to *Te*. *tilletti* [60]. The shaft is dorsoventrally flattened and mediolaterally wider as in *Convolosaurus* [64], yet does not exhibit the strongly strap-like condition of *Th*. *neglectus* [67].

#### Frontal

A partial right frontal missing the rostral-most portion and the rostral half of the midline suture is preserved (Fig 5F–5I). Its length can be estimated via the medial suture on the prefrontal and it was clearly rostrocaudally longer than transversely wide, in contrast to the more squat frontals of *Z*. *robustus* [61]. In dorsal view, the frontal is mediolaterally widest at the rostral-most contact with the prefrontal. The caudal portion is flat, unlike the dorsally concave condition of *Te*. *tilletti* [60] (Fig 5G–5I). In dorsal view, the caudal-most extension of the prefrontal suture of *Iani* is transversely narrow and tapers to a point (Fig 5F). The caudalmost extent of the suture for the prefrontal terminates halfway between the suture with the contralateral frontal and the orbital rim (Fig 2). A similar morphology is observed on *Convolosaurus* [64]. This contrasts with *Te*. *tilletti* [60], which bears a broad facet for the prefrontal with a caudalmost extent expressed at the orbital rim, and *Th*. *neglectus* and *Hypsilophodon*, in which the caudal-most extent is medial to the orbital rim, but only slightly so. The caudalmost extent of the prefrontal facet is sharply pointed in *Iani* and *Convolosaurus*, yet blunt in *Th*. *neglectus* [67], *Hypsilophodon* [69], and *Haya* [75].

The orbital rim is rugose and bears a supraciliary foramen directly on the margin just rostral to the postorbital facet. Due to its marginal location, it manifests as a groove rather than an enclosed foramen (Fig 5F). The contact with the postorbital is extensive. The rostral-most aspect curves medially to form a hatchet-shaped medial projection that terminates well medial to the orbital margin (Fig 2) as in *Convolosaurus* (SMU 74748); however, in *Iani* the rostral margin is straight (Fig 5F), whereas, in *Convolosaurus*, this margin is rostrally convex. The medial margin of this suture is laterally convex as in *Convolosaurus* (SMU 72834) and to a lesser degree *Z*. *robustus* [61], whereas in *Te*. *tilletti* this margin is laterally concave [60]. The rostromedial aspect terminates slightly medial to the lateral-most extent of the parietal suture (Fig 5F). This feature is not seen on *Te*. *tilletti* or *Th*. *neglectus*.

Despite some damage medially, the contact with the parietal appears to have been relatively horizontal in dorsal view (Fig 5F), with a slight caudal extension at the interfrontal suture as in *Th*. *neglectus* and *Convolosaurus* [64], as opposed to concave for reception of a rostral prong on the parietal as in *Hypsilophodon* [69]. The supratemporal fossa does not appear to have extended onto the caudal aspect of the frontal, likely being restricted to the parietal as in *Dysalotosaurus*, *Th*. *neglectus*, *Convolosaurus* (SMU 72834), *Iguanodon bernissartensis*, and *Mantellisaurus atherfieldiensis*, and in contrast to *Z*. *robustus* [74]. In ventral view, the crista cranii are expressed only as gentle rugosities (Fig 5H). A large rugose facet for the laterosphenoid is present lateral to the cerebral lobe (Fig 5H). It rims approximately half of the portion of the cerebral hemispheres on the frontal as in *Convolosaurus* (SMU 72834), thescelosaurids (MM15-MT-37-051), and *Te*. *tilletti* (OMNH 58340), in contrast to later diverging taxa such as the hadrosauroid *Prosaurolophus* [76, 77], in which the contact is so extensive as to entirely rim the cerebral hemispheres of the frontal.

#### Parietal

**Fig 6:**
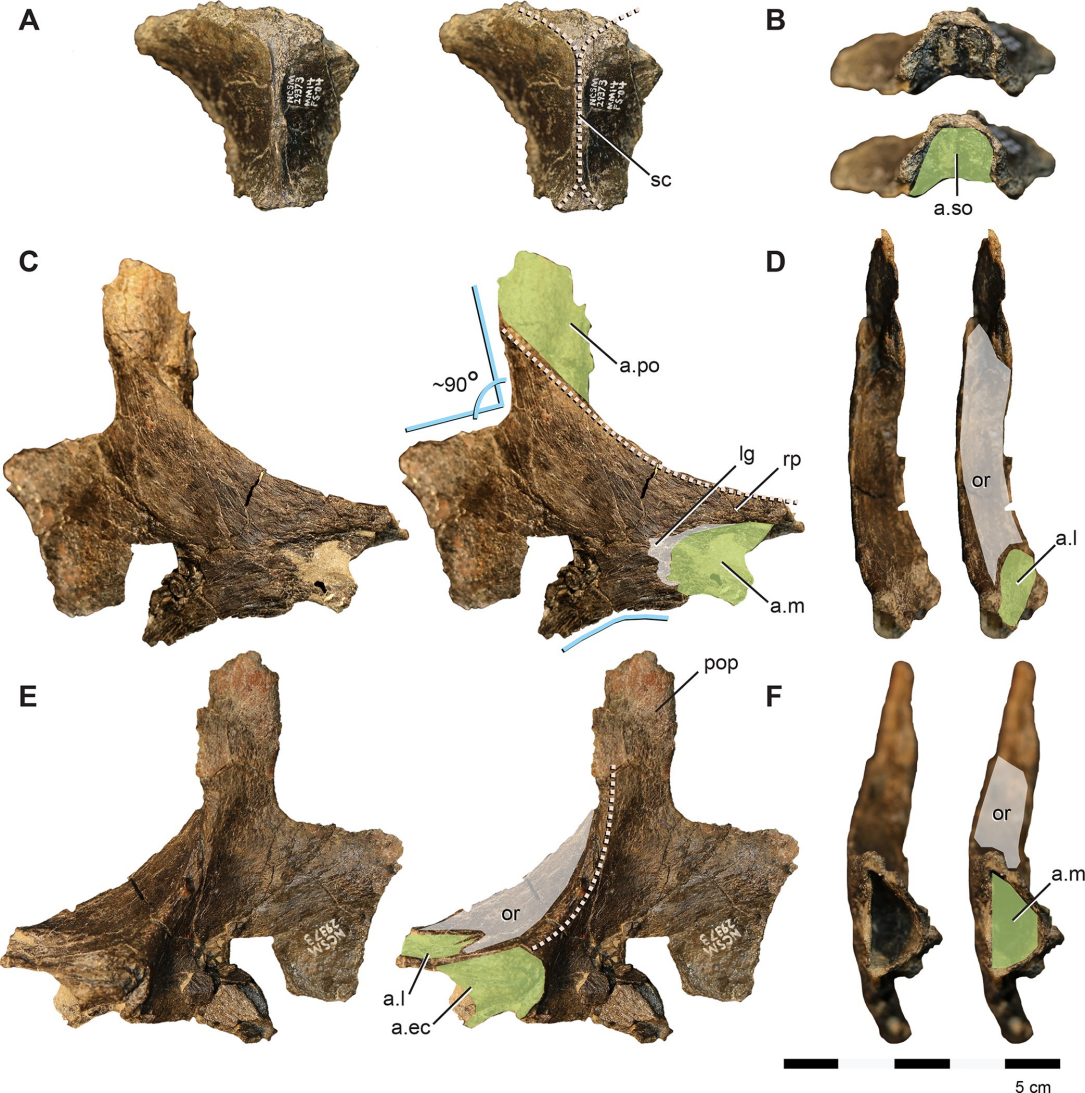
Parietal and jugal of *Iani smithi* (NCSM 29373). Partial paired parietals in (A) dorsal and (B) caudal views; right jugal in (C) lateral, (D) dorsal, (E) medial, and (F) rostral views. Abbreviations: a.ec, ectopterygoid articulation; a.l, lacrimal articulation; a.m, maxilla articulation; a.po, postorbital articulation; a.so, supraorbital articulation; lg, rugose lateral groove on the maxillary process of the jugal; or, orbit; sc, sagittal crest. Color annotation: white, depressions/fossae/grooves; green, articular surfaces; blue circles, foramina; light blue lines, marginal contours; peach dashed lines, ridges/internal contours. Scale bar 5 cm.

A pair of fused parietals are preserved, missing only the right craniolateral aspect (Fig 6A and 6B). In dorsal view, the parietals are fan-shaped, with a rostral transverse width more than double the caudalmost width. There is a well-developed sagittal crest bearing a mesial groove that bifurcates rostrally to rim the rostromedial margin of the supratemporal fenestrae as in most ornithopods. However, on NCMS 29373 the rostromedial margin of the supratemporal fenestra splays from the midline to the frontal suture only minorly (Fig 6A), as in *Th*. *neglectus*, and in contrast to the widely separated rims of *Zalmoxes* sp. (PIUW 2349/54) [78] and *Hypsilophodon* [69]. The condition on *Convolosaurus* (SMU 72834) varies from similar to that of *Iani* in the small juvenile specimen (SMU 74748) to widely separated and paired midline ridges that never meet in the large individual (SMU 72834), so this feature may be ontogenetically variable.

#### Jugal

The jugal (Fig 6C–6F) closely approximates the juvenile *Te*. *tilletti* specimen OMNH 08137. The dorsoventral height and rostrocaudal length are subequal, resulting in a robust, blocky, and relatively equidimensional element, with a subrectangular quadratojugal ramus (Fig 6C and 6E). In lateral view, the maxillary (rostral) ramus has parallel dorsal and ventral margins as in *Convolosaurus* (SMU 72834) and *Z*. *robustus* [61] (Fig 6C and 6E). This is listed as a synapomorphy of rhabdodontomorphs by Dieudonné et al., [37] but is present more broadly among ornithopods. As in *Z*. *robustus* [61], *Z*. *shqipororum* [79], and *Te*. *tilletti*, the rostral aspect has a subtriangular depression/groove for reception of the caudolateral process of the maxilla (Fig 6C), and bears a rugose fossa caudal to the extent of maxillary overlap (Fig 6D). In medial view, there is a deep, subtriangular, enclosed socket for the caudal body of the maxilla (Fig 6F) as on *Te*. *tilletti* (OMNH 08137), yet unlike the more open articular surface of *Z*. *shqipororum* [79] and possibly *Z*. *robustus* [61]. Matrix covers the maxillary articulation facet on *Convolosaurus* (SMU 72834) and it is not possible to determine if this feature is a peg and socket configuration as in *Iani*.

In dorsal view, there is a shallow depression on the rostral process with a wavy margin (Fig 6D). This likely represents the articulation with the lacrimal (Fig 6F), corresponding with its caudally deflected ventral process rimming the ventral orbit as in *Z*. *shqipororum* [79]. If so, it is unlike the butt joint articulation between the jugal and lacrimal on *Te*. *tilletti* [60]. Medially, there is a robust, rugose, crescent-shaped articulation for the ectopterygoid (Fig 6F). On *Te*. *tilletti* the ectopterygoid contact is “stalked” and separated from the maxillary articulation, whereas on *Iani* it is continuous with the maxilla facet and the ectopterygoid contact is not medially extended.

The postorbital process is a mediolaterally flattened, rostrocaudally wide strap of bone (Fig 6F). Medially, the rostral edge forms a sharp ridge that curves rostroventrally to contact the ectopterygoid process (shared with *Convolosaurus* [SMU 72834]) (Fig 6E). In lateral view, the caudal edge trends rostroventrally, becoming a sharp ridge that forms the rostroventral margin of the orbit as in *Te*. *tilletti* [60] and *Convolosaurus* [64]. The postorbital articulates via a scarf joint as in other ornithopods, except on its ventral-most tip. On *Iani* the ventral tip of the postorbital tucks into a small socket on the jugal at the caudoventral corner of the orbit. When viewed laterally, this part of the postorbital would have been obscured by the ventral margin of the orbit (Fig 6C).

The ventral margin of the jugal is damaged caudally. It is subtly concave ventral to the orbit (Fig 6E) and then widens caudally becoming sigmoid as in *Z*. *robustus* [61], *Z*. *shqipororum* [79], and *Te*. *tilletti* [60], more so than the relatively straight margins of *Hypsilophodon* [69] and *Convolosaurus* [64]. The quadratojugal (caudal) ramus is quadrilateral in shape with well preserved dorsal and caudal margins. The dorsal margin extends caudally at a right angle to the postorbital process, forming a nearly 90° angle at the rostroventral corner of the infratemporal fenestra (Fig 6C) as in *Tenontosaurus* (it is slightly acute in *Convolosaurus*, SMU 74678). This is in contrast to the dorsally rising dorsal margin of the quadratojugal process of *Zalmoxes* [61, 79]. It is not apparent on *Iani* how the quadratojugal articulates with the caudal jugal process because no impressions are present for scarf joints and the caudoventral margin is not preserved.

#### Postorbital

**Fig 7:**
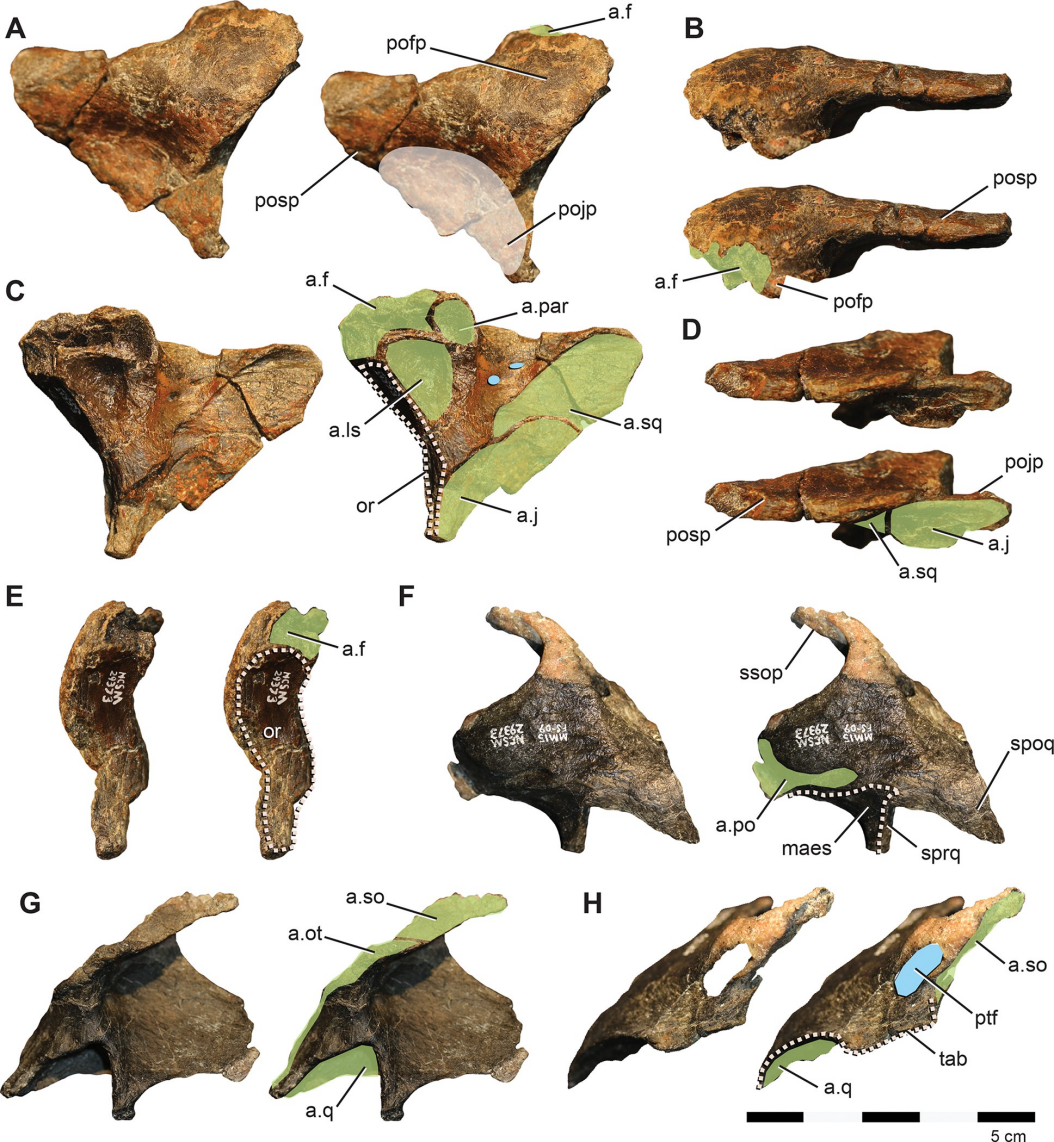
Postorbital and squamosal of *Iani smithi* (NCSM 29373). Right postorbital in (A) lateral, (B) dorsal, (C) medial, (D) ventral, and (E) rostral views; left squamosal in (F) lateral, (G) medial, and (H) caudal views. Abbreviations: a.f, frontal articulation; a.j, jugal articulation; a.ls, laterosphenoid articulation; a.par, parietal articulation; a.po, postorbital articulation; a.so, supraorbital articulation; a.sq, squamosal articulation; a.q, quadrate articulation; a.ot, otosphenoid articulation; maes, m. adductor externus superficialis; or, orbit; pofp, postorbital, frontal process; pojp, postorbital, jugal process, posp, postorbital, squamosal process; ptf, postemporal foramen; ssop, squamosal, supraorbital process; spoq, squamosal, postquadratic process; sprq, squamosal, prequadratic process; tab, squamosal tab. Color annotation: white, depressions/fossae/grooves; green, articular surfaces; blue circles, foramina; light blue lines, marginal contours; peach dashed lines, ridges/internal contours. Scale bar 5 cm.

A right and left postorbital are represented. The right postorbital is more complete, whereas the left is substantially eroded, affecting its thickness throughout. The postorbitals of *Iani* are robust and triangular (Fig 7A–7E), similar in form to *Convolosaurus* (SMU 72834) and *Te*. *tilletti*. The frontal process is craniocaudally extensive and braces the parietal caudally via an elongate caudomedial process that forms a substantial portion of the rostromedial margin of the supratemporal fenestra in dorsal view as in *Z*. *robustus* [61] (incompletely preserved on the right postorbital on Fig 7). The jugal and squamosal processes are connected by a broad sheet of bone, creating a straight caudoventral margin as in *Te*. *tilletti* (OMNH 58340) and *Haya* [75] (Fig 7A), and in contrast to the caudally concave margin of *Z*. *robustus* [61], *Convolosaurus* (SMU 72834), and *Th*. *neglectus* [67]. There is a concave depression in this region as is typical for ornithopods, yet it is more pronounced in *Iani*, largely due to a mediolaterally “inflated” dorsal aspect of the orbit, that rapidly tapers ventrally in rostral view. A similar, yet slightly less pronounced dorsal thickening of the postorbital is observed on *Te*. *tilletti* [60] and *Convolosaurus* (SMU 72834).

Several foramina pierce obliquely through the center of the main body (Fig 7C). Although missing its ventral-most extent, the jugal process does not appear to curve rostrally, and as a result, the orbital margin is weakly curved as in *Convolosaurus*, but in contrast to *Te*. *tilletti* [60] and *Th*. *neglectus* (NCSM 15728). In medial view, there is a deep, subtriangular socket for the head of the laterosphenoid restricted to the postorbital as in *Z*. *robustus* [61], *Convolosaurus* [64], and *Te*. *dossi* (FWMSH 93B2) (Fig 7C). The facet for the postorbital process of the jugal extends dorsally to nearly contact the squamosal facet, terminating caudally and slightly ventral to the laterosphenoid sulcus (Fig 7C and 7D).

#### Squamosal

The squamosal is relatively gracile and bears parietal (medial), postorbital (rostral), prequadradic, and postquadratic processes, as opposed to the triradiate condition of *Z*. *robustus* [61] (Fig 7F–7H). Together, the postorbital and rostrally extensive parietal process form more than the caudal third of the supratemporal fenestra, including the caudolateral and caudomedial margin (Fig 3), in contrast to rhabdodontomorphs, in which the parietal process does not form the medial aspect of the supratemporal fenestra [79]. A large posttemporal foramen is housed entirely within the parietal process of the squamosal (Figs 2 and 7H) as in *Z*. *robustus* [61] and *Z*. *shqipororum* [79] and in contrast to *Te*. *tilletti*, *Te*. *dossi* and *Th*. *neglectus*, where the posttemporal foramen divides the squamosal from the paroccipital process (OMNH 58340, pers obs LZ). The condition in *Convolosaurus* cannot be determined. Godefroit et al., [79] list the condition of having the posttemporal foramen entirely enclosed by the squamosal as a diagnostic feature of the genus *Zalmoxes*; however, it appears to have a wider distribution among rhabdodontomorphs.

The caudal margin of the squamosal is complex (Fig 7H). The caudal margin of the postquadratic process bears a dorsally concave notch for a correspondingly dorsally flaring paroccipital process of the otoccipital. Moving medially, a robust tab extends ventrally to brace the paroccipital process near its base (Figs 2 and 7H). Notably, this subrectangular tab descending from the caudal margin of the squamosal is also present on *Te*. *tilletti* [60] (MOR 2571). The caudal margin then angles sharply rostromedially at the contact with the supraoccipital to cap the parietal process. Although the ventralmost extent of the prequadratic process is broken, we estimate it was approximately subequal in length with the postquadratic process as in *Te*. *tilletti* [60] and *Convolosaurus* (SMU 74678) and in contrast to rhabdodontomorphs (e.g., *Z*. *robustus* and *Z*. *shqiperorum*) for which the postquadratic process is weak to absent [79]. The postorbital inserts into a caudally tapering groove on the lateral squamosal (Fig 7F) as in *Te*. *tilletti*, *Te*. *dossi* (FWMSH 93B2), and *Th*. *neglectus* (NCSM 15728). A gentle, subtriangular depression represents the insertion point for the m. adductor externus superficialis between the postorbital and prequadratic process (Fig 7F). A sharp dorsal margin demarcating this feature is absent as in *Convolosaurus* (SMU 72834) and *Z*. *robustus* [61], in contrast to the well-defined morphology of *Th*. *neglecus* [67], *Te*. *dossi*, and *Te*. *tilletti* [60]. In contrast to *Th*. *neglectus*, it does not extend onto the postquadratic process [76].

#### Quadrate

**Fig 8:**
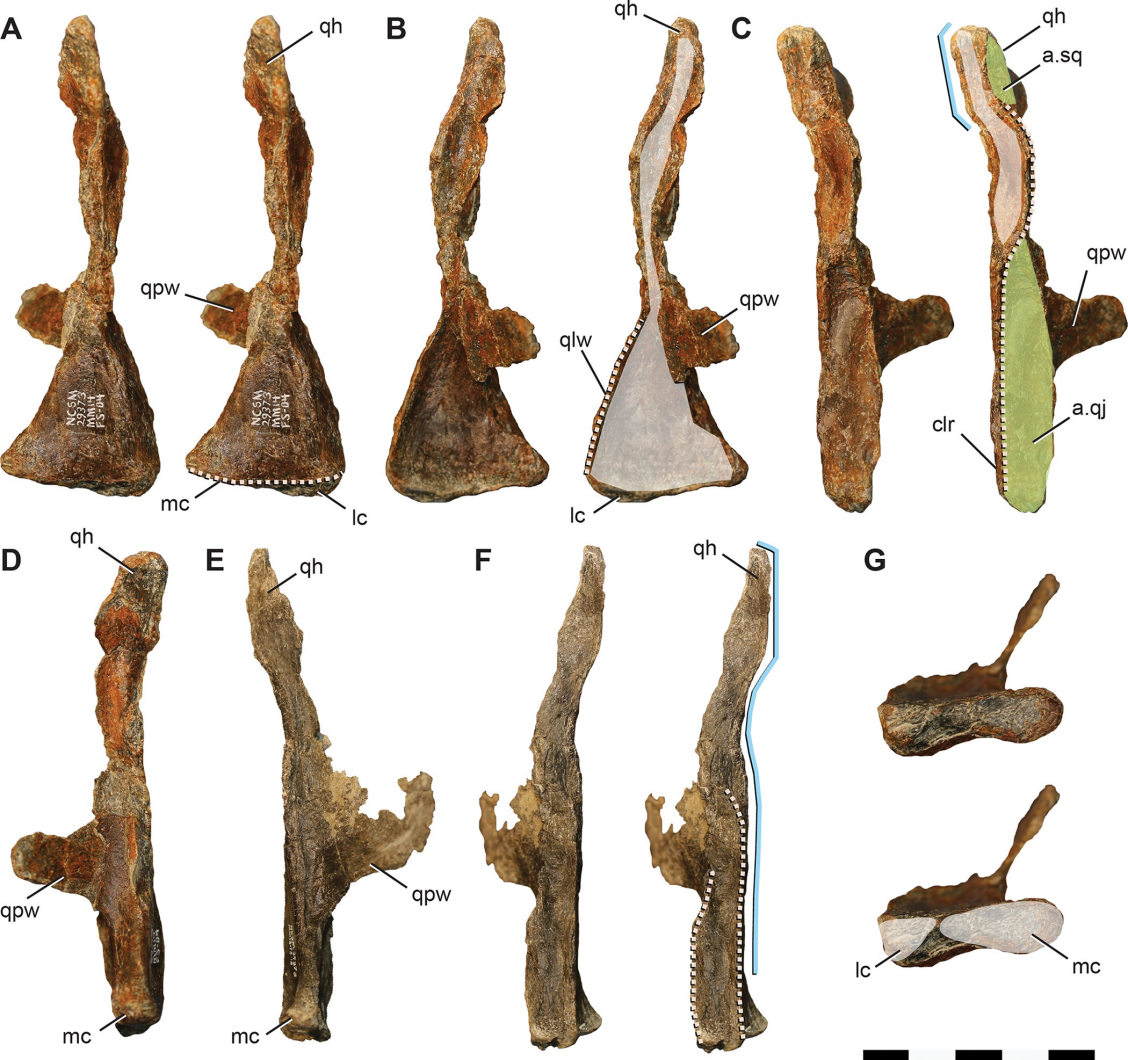
Quadrates of *Iani smithi* (NCSM 29373). Right quadrate in (A) caudal, (B) rostral, (C) lateral, (D) medial, and (G) ventral views; left quadrate in (E) medial and (F) lateral views. Abbreviations: a.sq, squamosal articulation; a.qj, quadratojugal articulation; clr, caudolateral ridge; lc, lateral condyle; mc, medial condyle; qh, quadrate head; qlw, quadrate lateral (jugal) wing; qpw, quadrate, pterygoid wing. Color annotation: white, depressions/fossae/grooves; green, articular surfaces; blue circles, foramina; light blue lines, marginal contours; peach dashed lines, ridges/internal contours. Scale bar 5 cm.

Nearly complete right and left quadrates are preserved. Both exhibit minor loss to the pterygoid wing, taphonomic compression, and distortion (Fig 8). Despite this, the quadrates appear more gracile than those of rhabdodontomorphs [39] and *Te*. *tilletti* [60]. The quadrate head appears only slightly deflected caudally (Fig 8C) unlike the strong deflection of rhabdodontomorphs [39, 79], *Te*. *tilletti* [60], and *Changchunsaurus* [80], although this may in part be preservational and is known to vary ontogenetically [81].

The caudolateral margin of the quadrate head is straight before angling rostrally, creating a subrectangular profile (Fig 8C and 8F). This feature is widely distributed among ornithischians (e.g., *Lesothosaurus*, *Th*. *neglectus*, *Changchunsaurus*, *Te*. *tilletti*) yet appears to be lost in rhabdodontomorphs (*M*. *vorosi* [39] and *Z*. *shqiperorum* [79]). Ventral to this angle, the body of the quadrate would have been straight (Fig 8C and 8F) as in *Hypsilophodon* [69], *Changchunsaurus* [80], and rhabdodontomorphs [39, 79] or slightly caudally convex as in *Te*. *tilletti* (OMNH 58340), but not rostrally convex as in *Lesothosaurus* [70].

The caudolateral margin of the quadrate shaft manifests as a sharp ridge delineating the caudal margin of the quadratojugal contact (Fig 8C). Ventrally, this ridge extends to contact the lateral mandibular condyle as in *Changchunsaurus* [80] indicating a similarly ventrally extensive quadratojugal. This is in contrast to *M*. *vorosi*, *Z*. *shqiperorum*, and *Th*. *neglectus*, and likely also *Te*. *tilletti* (OMNH 10132), in which the ridge terminates dorsal to the condyle. Dorsally, the caudolateral ridge curves rostrally near the midpoint of the quadrate shaft to circumscribe a concave facet that would have been rimmed by the dorsal process of the quadratojugal rostrally in lateral view (Fig 8C) as on *Te*. *tilletti* (OMNH 58340) [60] and *M*. *vorosi* [39]. In contrast, only a poorly defined ridge and fossa are present on the isolated quadrate of *Th*. *neglectus* (NCSM 15728).

The dorsal facet for the quadratojugal is difficult to discern; however, it appears the quadratojugal would have covered at least the ventral half of the quadrate body (based on the rostral curvature of the caudolateral ridge), if not extending dorsally far enough along the rostral margin of the lateral quadrate to contact the prequadratic process of the squamosal as in *Te*. *tilletti* [60]. A lateral (jugal) wing is present (Fig 8B). Ventrally it also contacts the lateral condyle, as in *M*. *vorosi*, yet in contrast to *Z*. *shqiperorum* [79]. In general form, it is weakly developed, similar to that of rhabdodontomorphs [39, 79]. The lateral wing exhibits a slight medial curvature, creating a concave facet on the rostral surface (Fig 8B) as in *Changchunsaurus* [80], *M*. *vorosi* [39], and *Z*. *robustus* [61]. On *Iani*, as in other ornithopods, this rostral concavity extends the entire length of the quadrate body to the dorsalmost aspect of the head of the quadrate (Fig 8B).

The ventral margin of the pterygoid wing originates well dorsal to the distal condyles (a distance of ~ 1/4th the total dorsoventral height of the quadrate) (Fig 8D and 8E) (proportionally more so than *Te*. *tilletti* [60] and less than in *M*. *vorosi* [39]) and extends to the dorsal quadrate cotylus. Thus the pterygoid wing comprises ~75% of the total dorsoventral height of the quadrate. A foramen near the ventral origination of the quadrate wing observed on *M*. *vorosi* [39] is absent. The lateral and medial distal condyles are similar in rostrocaudal breadth; however, the medial condyle is mediolaterally longer and bears a rostrally positioned flattened facet also seen on *Te*. *tilletti* (OMNH 10132) (Fig 8G). In caudal view, the lateral condyle projects ventrally as in *Z*. *shqiperorum* [79] and *Rhabdodon*, but not in *M*. *vorosi* [39]

#### Ectopterygoid

**Fig 9:**
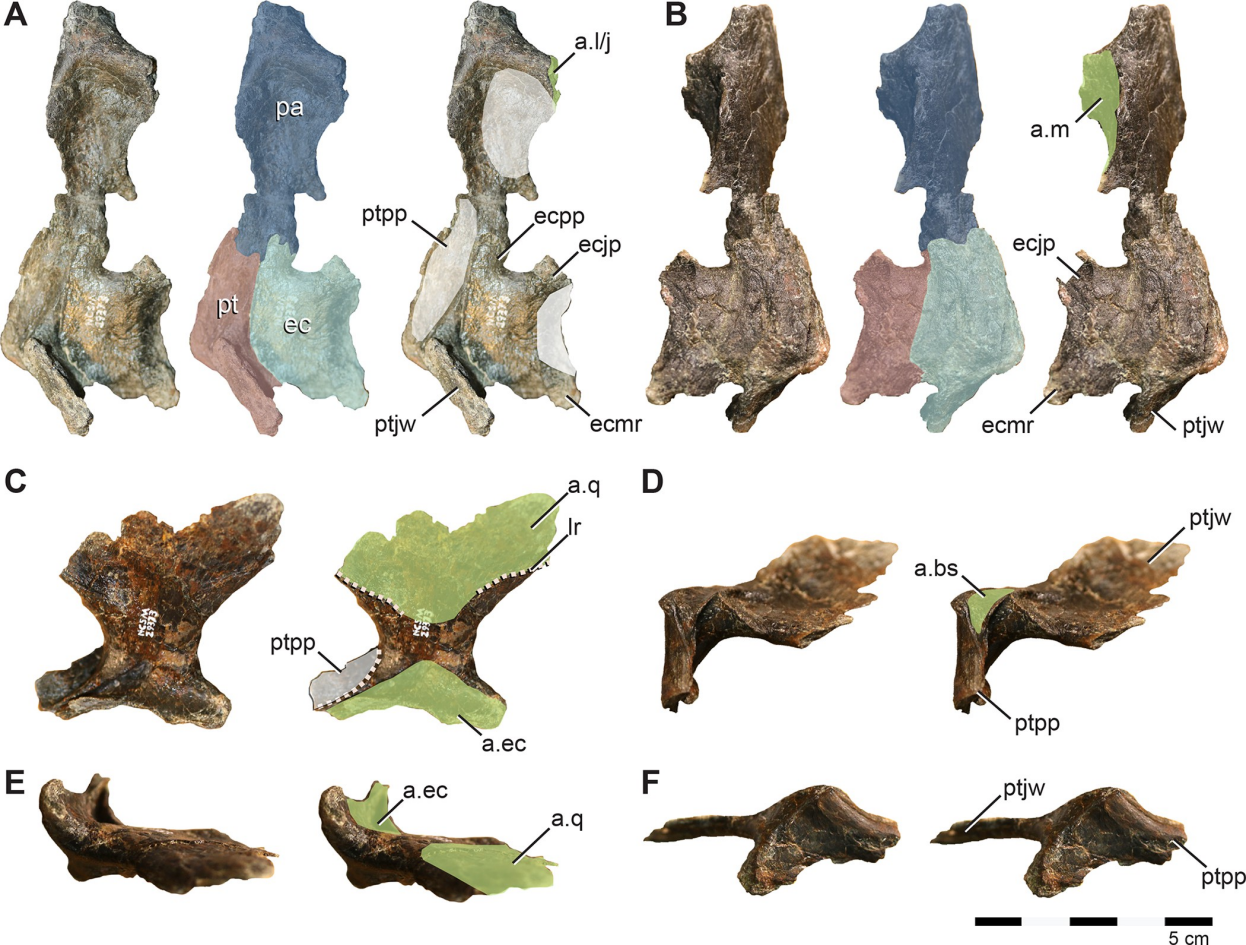
Palate of *Iani smithi* (NCSM 29373). Articulated right palatine, ectopterygoid, and pterygoid in (A) dorsolateral and (B) ventromedial views; isolated left pterygoid in (C) rostrolateral, (D) rostromedial, (E) caudomedial, and (F) ventromedial views. Abbreviations: a.bs, basisphenoid articulation; a.ec, ectopterygoid articulation; a.j, jugal articulation; a.l, lacrimal articulation; a.m, maxilla articulation; a.q, quadrate articulation; ec, ectopterygoid; ecjp, ectopterygoid, jugal process; ecmr, ectopterygoid; ecpp, ectopterygoid; palatine process; lr, lateral ridge; pa, palatine; pt, pterygoid; ptjw, pterygoid, jugal wing; ptpp, pterygoid, palatine process. Color annotation: white, depressions/fossae/grooves; green, articular surfaces; blue circles, foramina; light blue lines, marginal contours; peach dashed lines, ridges/internal contours. Scale bar 5 cm.

A partial right ectopterygoid is preserved in articulation with the right pterygoid and palatine (Fig 9A and 9B). The jugal process is damaged and its full length is unclear. The ectopterygoid has a robust pterygoid (medial) flange that is mediolaterally broad and contacts the pterygoid along the entire mandibular processes as in other early-diverging ornithopods. On *Th*. *neglectus*, the ectopterygoid contribution to the mandibular process is restricted such that the pterygoid extends significantly beyond the ectopterygoid laterally [67], whereas, on *Iani* this region of the ectopterygoid is substantial and it appears likely that it would have extended equal to (as in *Tenontosaurus* [60]), if not laterally beyond, the pterygoid (as in *Hypsilophodon* [69]). Rostrally, the ectopterygoid broadly contacts the palatine, excluding the pterygoid from the margin of the postpalatine fenestra (Fig 9A and 9B) as in *Haya* [75], and likely also *Changchunsaurus* [31]. It bears a deep notch between the palatine and jugal (lateral) processes, forming the caudal, and approximately half of the medial and lateral margins of the postpalatine (suborbital) foramen (Figs 2 and 9A and 9B). This feature contrasts with *Th*. *neglectus* [67] and *Te*. *tilletti* [60], in which the notch is absent and the ectopterygoid only contributes to the caudal margin, and to *Haya* in which it only forms the caudal and medial margin [31: fig 23]. Contact between the ectopterygoid and the palatine is widespread among ornithischians (e.g., *Lesothosaurus* [70], *Haya* [31], *Tenontosaurus* and possibly *Dryosaurus* [60]); it is indeterminate on *Hypsilophodon* [69] and is likely present on *Changchunsaurus* [31] but see [80].

#### Pterygoid

*Iani* includes a relatively complete left pterygoid missing only the medialmost aspect of the quadrate wing and part of the dorsal margin of the palatine ramus (Fig 9C–9F). A more fragmentary right pterygoid, preserving only the mandibular and palatine rami, and the base of the quadrate wing, and articulated with the ectopterygoid and palatine is also preserved (Fig 9A, 9B). The caudomedial aspect of the quadrate wing is concave. A u-shaped trough for reception of the basipterygoid processes (as observed on *Haya* and *Changchunsaurus* [31, 75] is absent; only a shallow facet is present and it is not dorsally displaced as in those taxa (Fig 9D). The rostrolateral face of the quadrate wing lacks the groove present on *Th*. *neglectus* [67]; however, a lateral pterygoid ridge for reception of the pterygoid wing of the quadrate is present (Fig 9D) as on *Th*. *neglectus* [67], *Haya* [31], and *Changchunsaurus* [80]. The pterygoid of *Iani* lacks a well-defined medial process such as that present on thescelosaurids (e.g., *Th*. *neglectus* and MM15-MT-37-018), yet possesses a more extensive mandibular ramus than observed on these taxa [67]. The palatine ramus underlies both the pterygoid process of the palatine and palatine ramus of the ectopterygoid medially in a lap joint, nearly obscuring contact between the palatine and ectopterygoid in medial view.

#### Palatine

The palatine is damaged along its caudodorsal margin. It is robust and increases in thickness rostrally (Fig 9A and 9B) as in *Hypsilophodon* [69]. The medial surface is flat and the lateral surface is medially concave. As in *Hypsilophodon* [69] and *Lesothosaurus*, a stalk-like bar extends laterally to brace the lacrimal and/or jugal (Fig 9A), in contrast to *Haya*, where the palatine only contacts the maxilla [31]. Ventral to this is a deep, rostrocaudally-elongate groove for articulation with the maxilla. There is a distinct caudomedial process projecting off the caudal aspect of the palatine that forms half of the lateral margin of the postpalatine foramen. This prong does not appear to be present and/or well-developed on the palatines of *Hypsilophodon*, *Haya*, *Th*. *neglectus*, or *Te*. *tilletti*.

#### Braincase

**Fig 10:**
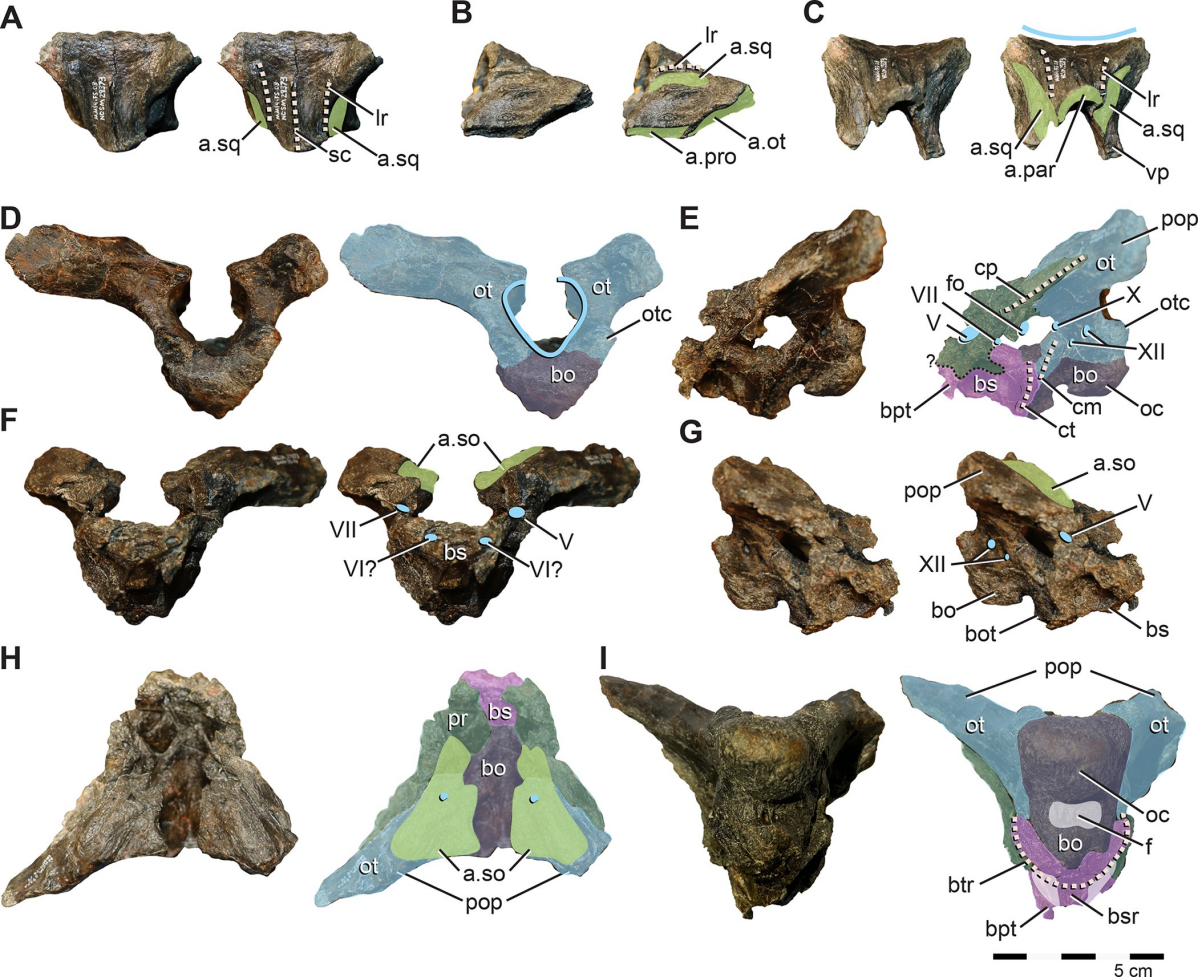
Braincase of *Iani smithi* (NCSM 29373). Supraoccipital in (A) dorsocaudal, (B) left lateral, and (C) rostral views; fused otoccipital, basioccipital, basisphenoid, prootic, and laterosphenoid in (D) caudal, (E) left lateral, (F) rostral, (G) right lateral, (H) dorsal, and (I) ventral views. Abbreviations: V, trigeminal foramen; VI, abducens nerve foramen; VII, facialis foramen; X, vagus foramen; XII, hypoglossal canal; a.par, parietal articulation a.so, articulation supraoccipitial; a.sq, articulation squamosal; bo, basioccipital; bot, basioccipital tuberosity; bs, basisphenoid; bsr, basisphenoid midline ridge; bt, basituberal ridge; bpt, basipterygoid processes; cm, crista metotica; cp, crista prootica; ct, crista tuberalis; f, fossa fo, foramen ovale; ls, laterosphenoid; oc, occipital condyle; ot, otoccipial; otc, otoccipital condylid; pop, paroccipital process; pr, prootic. Color annotation: white, depressions/fossae/grooves; green, articular surfaces; blue circles, foramina; light blue lines, marginal contours; peach dashed lines, ridges/internal contours. Scale bar 5 cm.

Most of the braincase is preserved; however the rostralmost and rostroventral portions are damaged and/or missing and the lateral walls of the braincase are eroded. Additional erosion to the right and left otic vestibules has resulted in artificially enlarged, singular openings contralaterally. Elements preserved include the left and partial right otoccipitals, basioccipital, partial basisphenoid, partial prootics, and possibly parts of the caudalmost laterosphenoids. Due to damage, erosion, and extensive cracking of the rostral braincase, it is difficult to distinguish sutures along the dorsal and lateral aspects, particularly the prootic/basisphenoid/laterosphenoid articulations. Sutures that can be confidently identified in gross inspection include defining the extent of the otoccipital and basioccipital and the nature of the basioccipital/basisphenoid contact in dorsal view (Fig 10). Rostral and lateral sutures between the basisphenoid, prootic, and laterosphenoids are indistinct and we caution that our interpretations of these sutures and corresponding neurovascular foramina (Fig 10) should be considered tentative until confirmed with x-ray computed tomographic data.

#### Supraoccipital

The supraoccipital appears to have contributed to a small portion of the dorsal foramen magnum (Fig 10D) as in *Rhabdodon* and *Zalmoxes* [65], *Te*. *dossi* (FWMSH 93B2), *Dysalotosaurus* [82], and *Haya* [31]; whereas a thin flange of otoccipital crowds out the supraoccipital from the foramen magnum on *Te*. *tilletti* [60]. The supraoccipital of NCMS 29373 is subtriangular shape in dorsal view as in *Te*. *tilletti* [60] and *Convolosaurus* [64]. The dorsal surface is concave (Fig 10C) in contrast to *Te*. *tilletti*, where the dorsal aspect of the supraoccipital is flat, and it bears a subtle, yet sharp, sagittal crest that extends only halfway from the cranial edge of the element (Fig 10A). This crest is well-developed on *Z*. *robustus* [61], yet termed the nuchal crest on that taxon, whereas a nuchal crest is variably considered a mediolaterally transverse feature (e.g., [60]; this paper). There are paired lateral ridges demarcating the dorsal from the lateral aspects of the rostral process of the supraoccipital (Fig 10B) as in *Convolosaurus* (SMU 72834), *Tenontosaurus* [60] (FWMSH 93B2); however, on *Tenontosaurus*, these ridges are robust and rounded instead of sharp as on *Iani*. Ventral to the ridges, deep furrows cut into the lateral supraoccipital for the squamosal that are supported dorsally and ventrally by rostrocaudally trending struts (Fig 10B). They extend across more than half the body of the supraoccipital. Rostroventral sides of the supraoccipital articulate with the parietal. In contrast to *Te*. *tilletti* [60], only slight ventral processes are present (Fig 10C).

#### Otoccipital

The otoccipitals (fused exoccipital and opisthotic) articulate with the prootics, basioccipital, and supraoccipital. In caudal view, the angle of contact between the otoccipital pillars and the basioccipital creates a ventrally pinched foramen magnum (Fig 10D) as in *Te*. *tilletti* [60] and *Te*. *dossi* (FWMSH 93B1), rather than the more circular foramen magnum of *Convolosaurus* [64] and *Dryosaurus* (CM 87688) [83], or the dorsoventrally-elongate, ovular opening of *Rhabdodon* (M4) [84] and *Z*. *robustus* [61]. Well-developed condylids (sensu [85]) on the dorsocaudal aspects of the ventral pillars (Fig 10D) comprise a substantial portion of the occipital condyle as in *Z*. *robustus* [61]. The paroccipital processes extend caudolaterally with no dorsal deflection; however, the ventral margin is damaged and it cannot be determined if a ventral hook was present. The relatively long, subhorizontal paroccipital processes are similar to those of *Rhabodon* MC-M1575 [65]. Two small foramina pierce the occipital pillars subhorizontally and are exposed in medial and lateral views. We interpret the caudalmost as the hypoglossal canal (CN XII) (Fig 10E and 10G). Just rostral to this, a second, smaller canal that does not merge medially, may have housed a second branch of CN XII if interpreted as homologous with *Th*. *neglectus* [67] or CN XI (spinal accessory nerve) if interpreted as homologous with that of *Te*. *tilletti* [60] and *Z*. *robustus* [61] (Fig 10E and 10G). Rostral to this, cutting into the caudal margin of the damaged external metotic region is what likely remains of the canal for the vagus nerve (CN X) (Fig 10E and 10G). The otoccipital extends ventrally along the contact with the basioccipital to form a bulbous crista metotica as in *Te*. *tilletti* [60] (Fig 10E and 10G).

#### Basioccipital

Forming the caudoventral region of the braincase, the basioccipital articulates with the basisphenoid through a narrow contact along the midline(Fig 10H) and contacts the prootics dorsolaterally. The occipital condyle exhibits a strong rostroventral orientation most similar to, yet more reclined than *Hypsilophodon* [69] and *Dryosaurus* [83]. Due to this, it is relatively compressed against the rest of the basioccipital, nearly lacking a “neck” (Fig 10E and 10G). Overall the occipital condyle is relatively reniform, if flattened somewhat caudally and ventrally, and bears a distinct ventral neck rimmed by a sharp, overhanging ventral margin (Fig 10D and 10E). This contrasts with the rostrocaudally-elongate, dorsoventrally flattened occipital condyles of *Rhabdodon* (​​MC-M4) [65], *Te*. *tilletti* (FWMSH 93B1) [60], and *Dysalotosaurus* [82] that also grade smoothly into the rostral basioccipital. Rostral to the occipital condyle the basioccipital grades into a tongue-shaped rostroventral body, bearing a weak depressed fossa on the midline of the rostroventral surface (Fig 10I). This weak depression also characterizes rhabdodontomorph basioccipitals from the Haţeg Basin [36] and possibly also *Rhabdodon* (MC-M4) [84]. On later-diverging hadrosauroids such as *Telmatosaurus*, this depression is more extreme [36].

On *Iani*, the ventral depression is punctured by paired basioccipital foramina divided by a midline lamina, a feature distinct to this taxon (Fig 2). A single ventral basioccipital foramen may also be present on the Haţeg rhabdodontomorph braincase LPB (FGGUB) R.1629 [36: Fig 5] and on *Te*. *dossi* (FWMSH 93B2). Moving rostrally, the basioccipital recurves caudally and bears a distinct tuberosity at its rostroventral most extent (Fig 10G and 10I). This feature is shared with *Rhabdodon* (MC-M1575) [65], rhabdodontomorphs of the Haţeg Basin [36], and *Te*. *tilletti* [60], where it is expressed variably as a ridge or pointed tuberosity. On *Tenontosaurus* [60] (FWMSH 93B2), this ridge is bordered laterally by an additional pair of foramina at the suture of the basioccipital and basisphenoids. On *Iani* the rostralmost basioccipital tuberosity is unique in nearly contacting the rostrally projecting ventral lip on the occipital condyle, forming a deep furrow in lateral view (Fig 10E and 10G; Fig 2).

The basioccipital wraps around the otoccipital pillars rostrally, reaching the lateral braincase, where it is exposed in lateral view (Fig 10E).

#### Basisphenoid

Only the caudal aspect of the basisphenoid is preserved. A wide ridge (damaged) runs the rostrocaudal length of the ventral basisphenoid (Fig 10I); lateral to this are paired depressions as in rhabdodontomorph basioccipitals from the Haţeg Basin [36], *Rhabdodon* (MC-M1575) [65], and *Te*. *tilletti* [60]. The basipterygoid processes are incompletely preserved (Fig 10E and 10I). In dorsal view, the basisphenoid makes up only a small medial strip of the endocranial floor. It is transversely narrowest caudally and expands, making up a greater portion of the floor of the endocranial space rostrally (Fig 10H).

The basal tubera form a rostrocaudally compressed ridge or “fan” buttressing the basioccipital as in by Haţeg rhabdodontomorphs [36] and *Tenontosaurus* [60, 62] (Fig 10I). On *Te*. *tilletti* this basituberal ridge is also interpreted as forming the crista tuberalis and is comprised of the basisphenoid [60]. On *Dryosaurus*, the basisphenoid possesses similar contralateral “stalks” that wrap dorsocaudally to embrace the rostral basioccipital. On *Iani* these processes overlap the basioccipital laterally and form a pronounced crista tuberalis that nearly extends to the midline of the transversely pinched basisphenoid (Fig 10E and 10I). It is unclear in gross inspection, which element comprises these features, as there appears to be a suture medially between this feature and the basisphenoid and between this feature and the prootic; however, given the phylogenetically bracketed condition of *Te*. *tilletti* and *Dryosaurus* [86], it is likely that this is comprised of the basisphenoid. Ultimately, the configuration needs to be confirmed via CT scan data, and presently we leave this feature unassigned (Fig 10E and 10I). Such a configuration may also be present on *Rhabdodon* (MC-M1575) [65]; however, it is unclear which element contributes to this feature in that taxon.

#### Prootic

The left and right prootics are incompletely preserved on *Iani* and due to damage and contralateral erosion of the otic region, the dorsal and rostral prootic boundaries are indistinct. Nonetheless, the prootic appears to have comprised most of the dorsolateral braincase wall (Fig 10E). Caudally, the prootic extends along a substantial portion of the paroccipital process and bears a pronounced crista prootica, originating its caudalmost extent and continuing across the dorsolateral braincase (Fig 10E). Although damaged, it appears likely to have reached the contact with the laterosphenoid dorsal to the trigeminal foramen (CN V) (Fig 10E). The foramen ovale, foramen metotica, and crista interfenestralis are eroded and unidentifiable; however, the approximate location of the foramina ovalis and metotica can be estimated based on the opening to the vestibule of the inner ear (Fig 10E and 10G). The prootic contributes to the dorsal, caudal, and ventral margins of CN V, yet does not completely enclose it (Fig 10E). Rostrally, the prootic may laterally overlap a small portion of the laterosphenoids.

#### Laterosphenoid

A small portion of the caudoventral laterosphenoids may be present and sutured to the basisphenoid ventrally and prootic medially. If correctly interpreted, these elements encroach on the basisphenoid medially as in *Te*. *tilletti* [60], but they do not completely obscure the basisphenoid from participation in the ventral floor of the endocranial space. They also contribute to the rostral margin of the trigeminal foramen.

#### Lower mandible

A fragmentary predentary, complete pair of dentaries, right surangular, partial splenial, and several isolated dentary teeth are preserved.

#### Predentary

**Fig 11:**
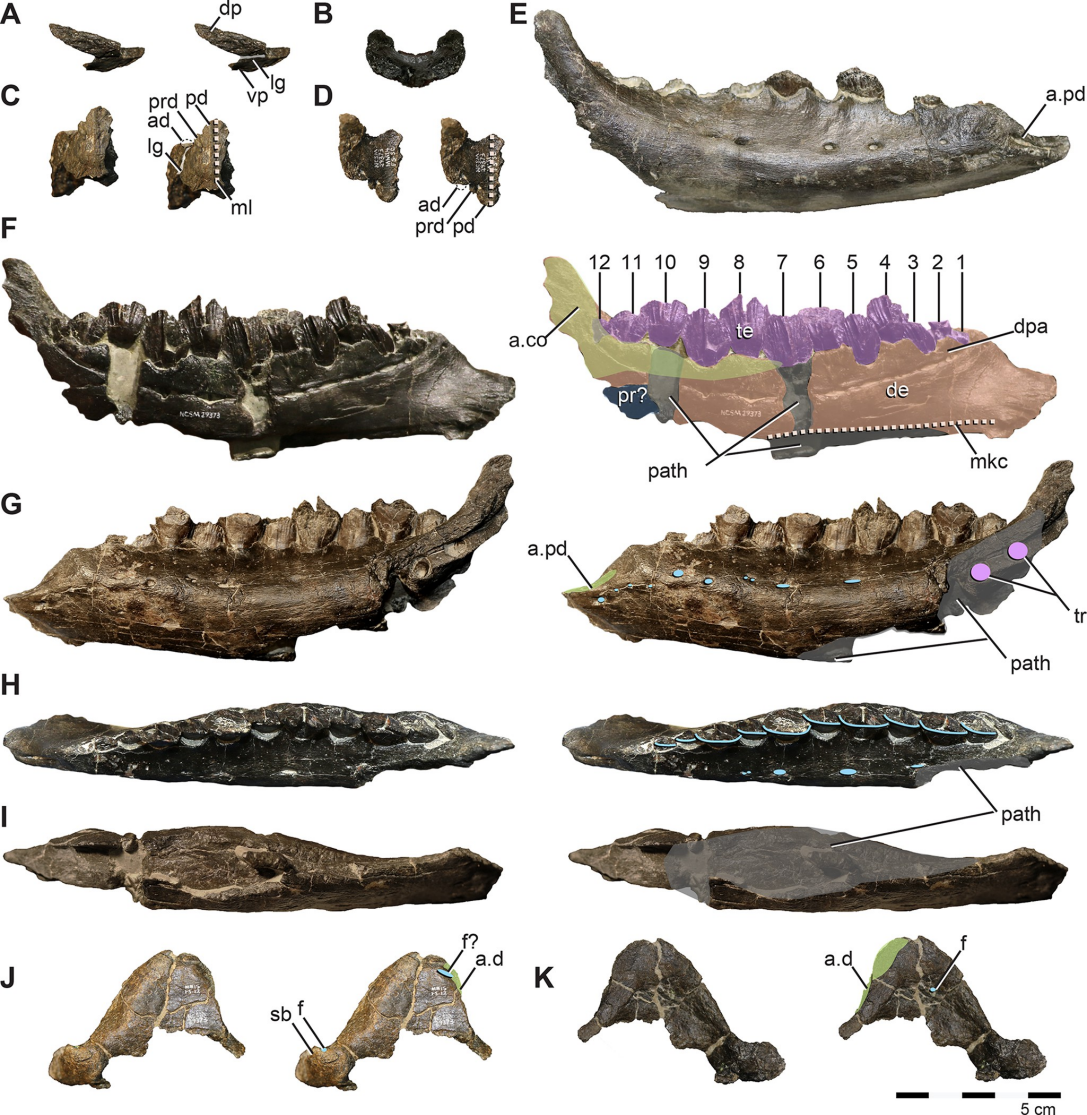
Predentary, dentary, and surangular of *Iani smithi* (NCSM 29373). Predentary in (A) right lateral, (B), rostral (right half mirrored and combined to show approximate shape of complete element), (C) ventral, and (D) dorsal views. Right dentary in (E) lateral view; left (pathological) dentary in (F) medial, (G) lateral, (H) dorsal, and (I) ventral views; right surangular in (J), lateral and (K), medial views. Abbreviations: a.co, estimated coronoid articulation; ad, accessory denticle; a.pd, predentary articulation; a.sr, surangular articulation; de, dentary; dp, dorsal process; dpa, dentary parapet; f, foramen; lg, lateral groove; mkc, Meckelian canal; ml, midline; path, pathological bone; pd, primary denticle; pathological bone; pr?, prearticular?; prd, peripheral denticle; sb, surangular boss; te, teeth; tr, exposed tooth root; vp, ventral process. Color annotation: white, depressions/fossae/grooves; green, articular surfaces; blue circles, foramina; light blue lines, marginal contours; peach dashed lines, ridges/internal contours; light purple, dentition. Scale bar 5 cm.

The predentary is nearly complete and preserved in two pieces. In general form, it is intermediate between the u-shaped condition of *Te*. *tilletti* [60] and *Z*. *shqiperorum* [79], and the narrow and tapering V-shaped morphology of *Haya*, *Changchunsaurus*, and *Th*. *neglectus* [67, 75, 80] and elasmarians (e.g., *Talenkauen* [63]) (Fig 11B). The oral margin is denticulate (Fig 11C). It exhibits a single rostrally extensive primary denticle (sensu [60]) with a diminutive pair of peripheral denticles, and an additional pair of denticles lateral to the dorsal process that are damaged (Fig 11C). *Te*. *tilletti* also exhibits three main denticles and also has additional minor accessory lateral denticles [60]. The dorsal process and accessory lateral denticles are divided by a deep groove that extends from the dentary articulation all the way to the oral margin (Fig 11A and 11C) as in *Haya* [75]; yet unlike in *Te*. *tilletti* [60], *Convolosaurus* [64], and *Th*. *neglectus* [67] in which the groove is less well developed and terminates prior to the oral margin. Due to damage, it is unclear if the ventral process was bilobate; however, the articular surfaces preserved on the dentary indicate the ventral process did not extend further caudally than the lateral processes, unlike *Convolosaurus* [64].

#### Dentary

The opposite dentaries of NCSM 29373 are asymmetrical. The left dentary is pathological, bearing multiple regions of bone resorption/remodelling medially, laterally, and ventrally (Fig 11F–11I). These regions are identified as pathological bone resorption and remodeling rather than taphonomic damage because they are emarginated by rugose, reactionary bone visible via in gross inspection (Fig 11I) and internally via CT-scanning. Two tooth roots are bent abnormally to extrude laterally into the caudalmost region of resorption (Fig 11G). Detailed description of these pathologies is beyond the scope of this manuscript. The right dentary is free from pathological indicators, but was recovered from the surface and bears slight erosion of the ventral region including the symphysis. Pathological bone resorption is generally localized to the tooth roots and the Meckelian groove (Fig 11F–11I). Resorption occurs at the caudal-most three alveoli laterally (exposing curved roots), and the third and sixth alveolus medially. A large region of bone resorption (about one-half the length of the tooth row) is present ventrolaterally, originating from the Meckelian groove. Given that neither dentary is free from pathology and weathering, we used what appears to be the most unaltered regions of both dentaries for trait characterizations and descriptions.

The dentaries are robust (max-width to max-height ratio ~74–78%) and nearly identical in form and proportion to *M*. *suessi* (PIUW 2349/2). In lateral view, the dentary is relatively straight, tapers slightly rostrally, and bears subparallel dorsal and ventral margins (Fig 11E and 11G). The ventral margin is slightly ventrally convex in lateral view as in rhabdodontomorphs (e.g., [39]) and some specimens of *Jeholosaurus* [87], in contrast to *Te*. *tilletti*, other iguanodontians, and thescelosaurids in which it is slightly sigmoidal (i.e., ventrally concave caudally and convex rostrally). The tooth row is deeply inset from the lateral face of the dentary creating a subhorizontal dentary shelf (Fig 11F) with a subtriangular cross-section. The buccal (lateral) ridge is gently convex as in *Z*. *robustus* and lacks the sharper profile of *Rhabdodon* sp. and *M*. *vorosi* (MC 443) [39], and “*M*.” *suessi* (ridge restricted to the caudal portion [78]. The ridge is located at approximately mid-height (~55%) as in rhabdodontomorphs [39] and *Te*. *tilletti* (OMNH 58340) as opposed to thescelosaurids, in which this inflection point is nearer to one-third the dorsoventral height of the dentary (e.g., *Th*. *neglectus*) and *Changchunsaurus*. The caudolateral depression on the lateral dentary of some rhabdodontomorphs (e.g., *M*. *vorosi*; [39]) and thescelosaurids (e.g., *Th*. *neglectus*, NCSM 15728) is absent on *Iani* and *Tenontosaurus*. The dentary symphysis curves rostrodorsally and medially as in “*M*.” *suessi* (PIUW 2349/2), and bears a distinct groove on the dorsal surface for articulation with the predentary (Fig 11E and 11F). This groove is consistent in width, unlike the caudally expanding groove of *M*. *vorosi* [39]. In lateral view, the symphysis is relatively symmetrical, with a gently arching ventral aspect (Fig 11E). The rostralmost point is located near mid-height (Fig 11G). The tooth row extends caudal to the rostralmost rise of the coronoid process, hiding the single caudalmost alveolus from lateral view, as in *Th*. *neglectus* [67], *Convolosaurus* [64], and non-hadrosauroid iguanodontians (e.g., [88]) (Fig 11F). The region between the caudalmost tooth position and the coronoid process is depressed as in *Te*. *tilletti*.

In dorsal view, the medial face of the dentary is arched and the lateral margin is relatively straight as in *M*. *suessi* (PIUW 2349/2) [78]. As a result, the tooth row is equidistant from the lateral margin across the caudal half of the dentary (Fig 11H), as in rhabdodontomorphs generally. This contrasts with the condition of *Te*. *tilletti* (OMNH 58340), where both the medial and lateral margins arch confluently, such that the distance between the tooth row and buccal ridge steadily increases caudally. The Meckelian canal extends rostrally to the point of contact with the predentary (Fig 11F). Replacement tooth crowns are visible within the series of slit-like alveolar foramina at the base of the alveolar parapet.

#### Surangular

The surangular is well preserved with only minor damage to the ventralmost rostral wing and some loss to the retroarticular process. The rostral margin of the rostral wing is sigmoidal, whereas, the caudal margin is dorsally arched (Fig 11J and 11K). This is in contrast to *Te*. *tilletti* (OMNH 58340) and *Z*. *robustus* (NHMUK [BMNH] R.4903), which have a subtriangular wing with comparatively straight caudal margins and slightly concave rostral margins [60, 61]. Due to damage to the retroarticular process, its caudal extent is unclear. Similarly, the lateral aspect of this region is poorly preserved so the exact morphology cannot be determined although a boss or lip is clearly present (Fig 11J) as in many neornithischians, e.g., *Haya* [75], *Changchunsaurus* [80], *Te*. *tilletti* [60], *Choyrodon* [88].

Foraminal terminology on the ornithischian surangular is inconsistent, a problem compounded by extreme variation in the size, shape, position, and number of surangular foramina interspecifically, intraspecifically, and even contralaterally [67]. For example, Boyd [67] considers a foramen positioned near the dentary-surangular contact on *Th*. *neglectus* the “surangular foramen.” A foramen in this area is widespread in early-diverging ornithischians, and is seen on *Hypsilophodon* [69], *Gasparinisaura* [89] and *Convolosaurus* [64], and may represent the remnant of the external mandibular fenestra (EMF) [89]. It was termed the “accessory surangular foramen by Norman et al., [90]. On *Changchunsaurus*, *Te*. *tilletti*, and *Choyrodon* a foramen positioned just rostral to the lateral process (lip) is dubbed the “surangular foramen” [60, 80, 88] and “surangular foramen sensu stricto” [90], whereas, a likely homologous feature is named the “lateral process foramen” on *Th*. *neglectus* [67] and *Haya* [31]. Yet a third foramen that pierces the lateral process/boss is present on the right surangular of *Th*. *neglectus* (NCSM 15728), *Haya*, and *Changchunsaurus* [75, 80: Fig 8B] is more consistent in position with the “surangular foramen” of *Lesothosaurus* in being on or ventral to the lateral lip as opposed to rostral to it [90]. A foramen centrally located within the rostral wing on *Z*. *shqiperorum* (UBB NVZ1-1) is considered a remnant EMF [79]; whereas on *Z*. *robustus*, a foramen in this region is variably termed the EMF (BMNH.R 4903) or the “surangular foramen” (BMNH R.3390) [61]. We observe several features on the rostral wing of the surangular on *Iani* including a small medial foramen within the abductor fossa without a clear lateral exit (Fig 11K), and the remnants of a channel at the rostralmost margin at the dentary contact that is similar in form and position to that of the “surangular foramen” (sensu [67]) of *Th*. *neglectus* (NCSM 15728) (Fig 11J). *Iani* also preserves a small foramen just dorsal to the lateral boss (Fig 11J) that may be homologous with the “lateral process foramen” sensu Boyd [67] and the “surangular foramen” of *Te*. *tilletti* sensu Thomas [60].

Avrahami et al., [91] recommend a standardized nomenclature for surangular foramina based on the internal branching structure of neurovascular canals proximal to the lateral process/boss (derived from CT scan data). They dub the three foramina (piercing the lateral process/lip, just rostral to the lateral process, rostrally located on the rostral wing near the dentary contact) the lateral process, accessory, and surangular foramina, respectively. Given that neornithischians reportedly possess between one and three distinct foramina on the surangular, that these foramina migrate in position, and moreover, that they are commonly used and discrepantly coded in phylogenetic matrices of ornithischian taxa (see e.g., [32, 35], additional research is needed to sort out the homology (and nomenclature) of these features more broadly across ornithischians.

#### Coronoid

The articular surfaces for the coronoid are preserved on the right and left dentaries and right surangular, indicating a relatively substantial, L-shaped element as in *Te*. *tilletti* that extended rostrally for approximately half of the dentary tooth row (Fig 11F). It is not possible to discern if a ventral process (as in *Th*. *neglectus* [67]) was present. The rostral process of the coronoid was dorsally positioned, overlapping the dental parapet and resting just ventral to the alveoli buttresses as *Th*. *neglectus* and *Te*. *tilletti* [60, 67]. From the preserved articular surface, we estimate that the rostral process terminated ventral to the eighth or ninth alveolus.

#### Prearticular

A fragment of transversely thin bone is preserved on the ventral dentary, in line with the second and third alveolus (Fig 11F). This either represents the dorsal process of the splenial or the dorsal extent of the prearticular.

#### Splenial

An isolated fragment of thin, striated bone likely represents a portion of the splenial (not figured).

#### Dentition

Overall, the dentition of *Iani* is most similar to that of early diverging ornithopods such as *Tenontosaurus*, rhabdodontomorphs, and *Qantassaurus* [39, 60, 61, 92]. All erupted teeth are preserved on the left dentary, although the tooth crowns of the mesialmost teeth are damaged. Five functional teeth are in situ within the right dentary. An additional 13 teeth were found isolated including eight maxillary and five dentary teeth, and one indeterminate tooth; many are poorly preserved. Enamel is present on both sides of the crown.

#### Premaxillary dentition

A single premaxillary tooth is exposed in lingual view (Fig 4D). Only the apical portion is visible. Overall, it is similar in form to a premaxillary tooth from an indeterminate rhabdodontomorph (MDS-VG,3) [37] in being labiolingually compressed, with a lingually recurved tip bearing pointed denticles, and a weak lingual keel that divides mesial and distal concavities on the crown (Fig 4D). At least one weak mesial ridge appears to be present on the lingual surface.

#### Maxillary dentition

**Fig 12:**
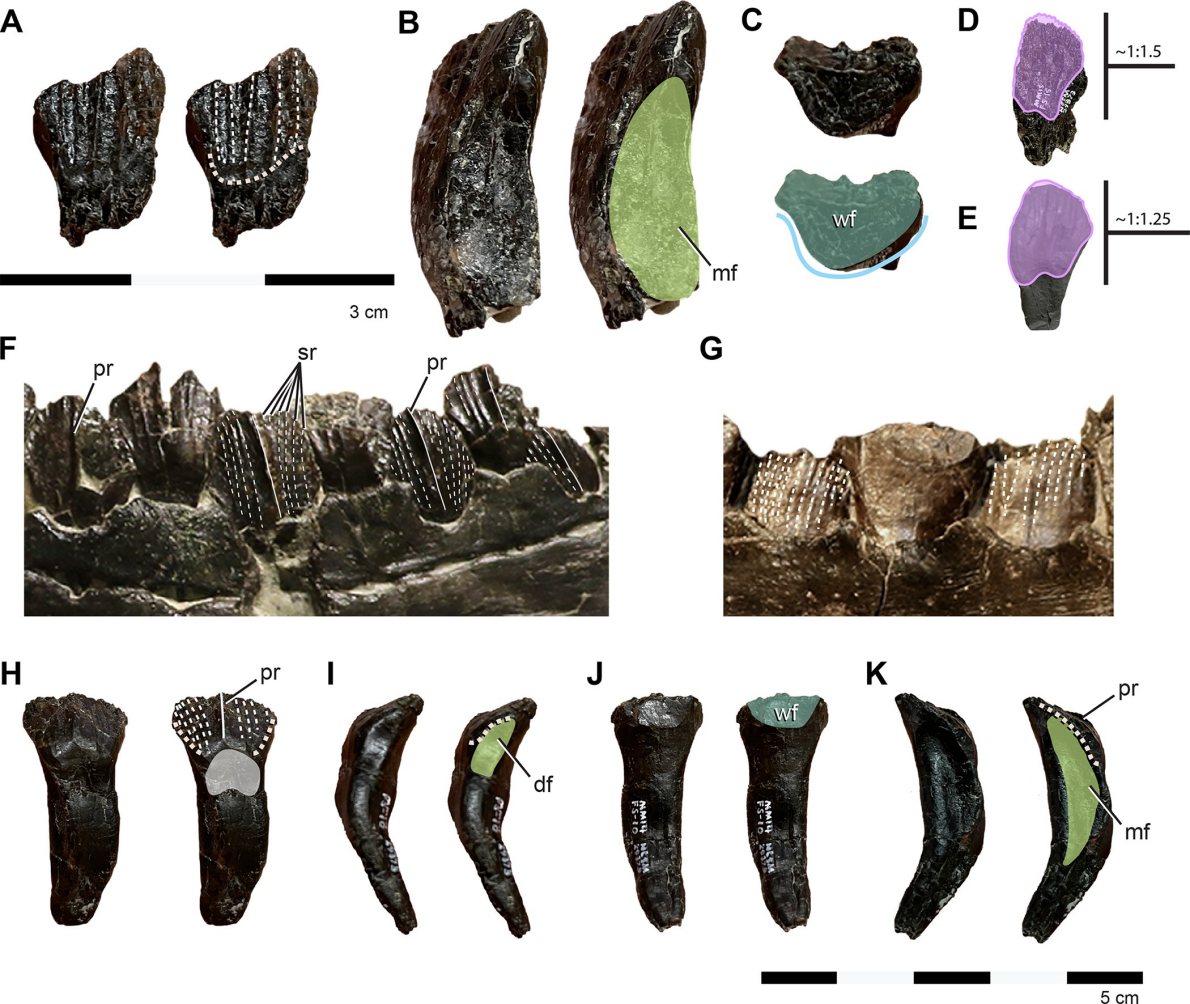
Buccal dentition of *Iani smithi* (NCSM 29373). Maxillary teeth FS-13 in (A) labial (C) occlusal views; FS-13B in (B) mesial? view, and (D) FS-16 in labial view showing reconstructed outline of unworn crown. (E) dentary tooth FS-SC showing reconstructed outline of unworn crown. In situ left dentary tooth crowns in (F) lingual and (G) labial views. Isolated dentary tooth crown and root FS-10 in (H) lingual, (I) distal, (J) labial, and (K) mesial views. Abbreviations: df, distal fossa; mf, mesial fossa; pr, primary ridge; sr, secondary ridge; wf, wear facet. Color annotation: white, depressions/fossae/grooves; green, articular surfaces; blue circles, foramina; light blue lines, marginal contours; peach dashed lines, ridges/internal contours. Scale bars (A–C) 3 cm; (H–K) 5 cm; (F–G) See Fig 11(D) and 11(E) not to scale.

All maxillary teeth are isolated and incomplete and/or taphonomically distorted. They appear asymmetrical in outline in buccal view, and subrectangular when compared to the dentary crowns, being taller than wide (~150%) as in *Z*. *robustus* [61]. The maxillary crowns are relatively flat labially, with only a slight lingual deflection near the root-crown junction in mesiodistal view (Fig 12B). The lingual surfaces are slightly bulbous (labiolingually and mesiodistally convex) apical to the root/crown junction, creating a lenticular cross-section as noted for *Z*. *robustus* [61] (Fig 12C). Labially, crowns bear a weakly developed primary ridge and a total of four to six, well-defined, parallel secondary ridges, with additional incompletely developed ridges confluent with the denticles that terminate prior to reaching the crown base. On some maxillary teeth, some secondary ridges are as well developed as the central ridge and thus no real “primary” ridge exists (Fig 12A). This variation is similar to that observed for *M*. *vorosi*, which displays some maxillary teeth with a more prominent central longitudinal ridge and lesser secondary ridges, and some with more similarly developed longitudinal ridges across the labial crown surface [39]. Likewise, some maxillary teeth bear evidence of faint ridges on the lingual face near the crown base as in *M*. *vorosi* [39], *Te*. *tilletti*. There are approximately eight to 12 denticles on either side of the median ridge on the maxillary teeth. Unlike *M*. *vorosi* [39] and *Te*. *tilletti* (OMNH 58340) there is no evidence of a pronounced ridge emarginating the basal margin of the crown, although the base of the crown is slightly thickened and the labial face appears slightly inset (Fig 12A). It is unclear if the roots of the maxillary teeth were curved due to crushing.

#### Dentary dentition

The dentary bears a low number of large teeth arranged in 12 tooth families (Fig 11F), as in other non-iguanodontian ornithopods (e.g., 10 in *Quantassaurus* and *M*. *suessi* [39, 93], 11 in *Convolosaurus* [64], ~12 in *Weewarrasaurus* [27], <12 in “Victoria Dentary Morphotype 3” (NMV P252006, [94]), and some specimens of *Tenontosaurus* (e.g., 12 in *Te*. *dossi* FWMSH 93B2 [62] but not *Te*. *tilletti* (OMNH 58340), which bears 14 [60]). Tooth count is known to vary ontogenetically in ornithopods [39, 64] although not isometrically [94]. Dentary teeth trend slightly apicodistally along the axis and are oriented en echelon (mesiolingually-distolabially) as in other ornithopods (e.g., [61]) (Fig 11H). The largest crowns are located in the middle of the tooth row. They decrease in apicobasal height and transverse width mesially and distally as in *Te*. *tilletti* [60] and rhabdodontomorphs [61, 79] (Fig 11F), and the mesialmost and distalmost teeth are substantially smaller (the distalmost crown is approximately one-half the mesiodistal width of the middle-most crown). Dentary teeth differ from the maxillary dentition in having more diamond-shaped crowns that are labiolingually thickest near the root crown junction, strongly convex lingually, and only slightly convex labially in mesiodistal view, with strongly recurved roots (Fig 12). Tooth crowns are tightly packed and overlapping.

**Fig 13:**
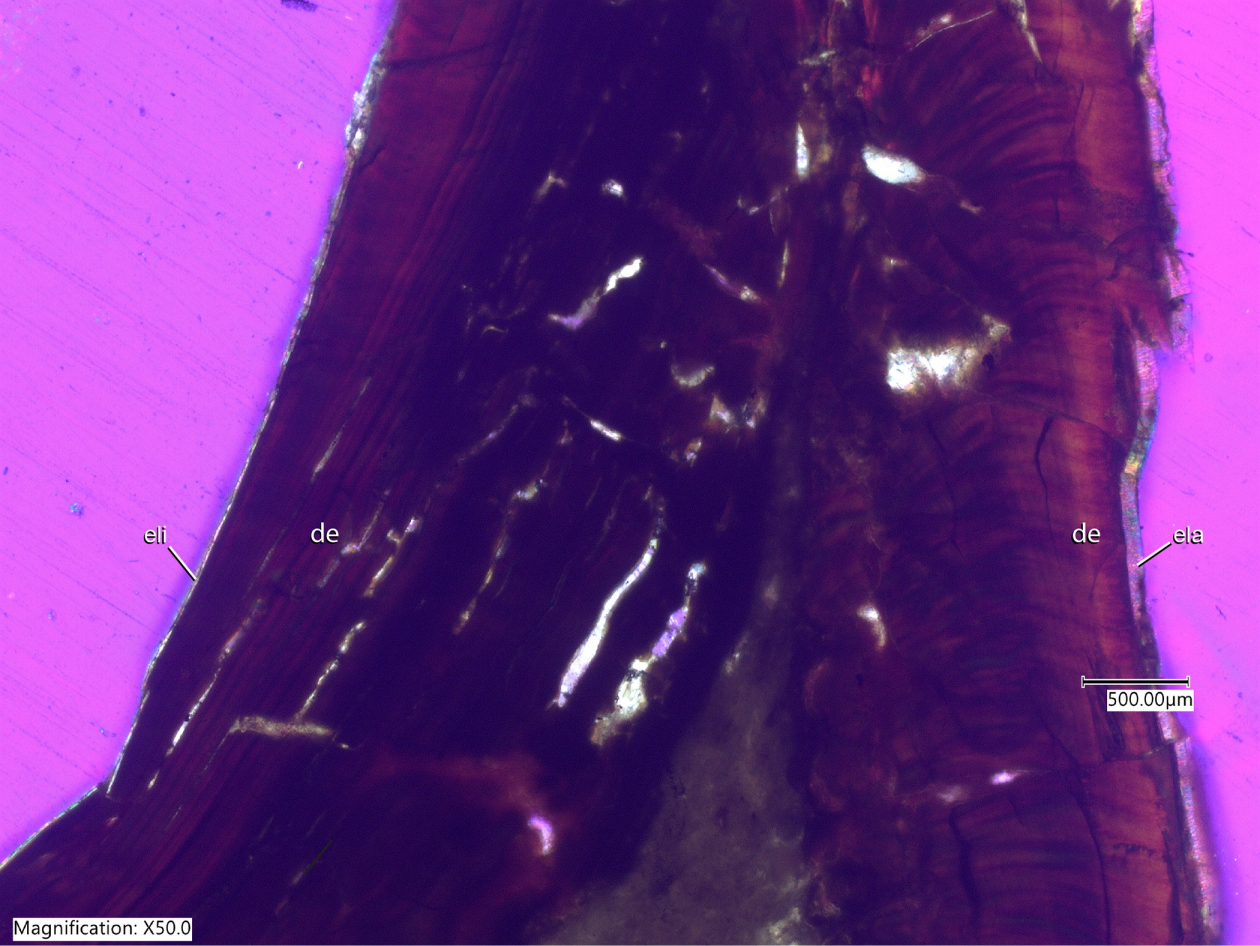
Labiolingual paleohistological section of the dentary tooth of *Iani smithi* (NCSM 29373). Abbreviations: de, dentine; eli, enamel on the lingual surface; ela, enamel on the labial surface; pc, pulp cavity. Image in transmitted polarized light with λ filter. Scale bar 500 μm.

The mesial aspect of the crowns bears a distinct groove that continues onto the root to accommodate the crown of the neighboring tooth (Fig 12I and 12K) as in *Zalmoxes* [61, 79] and *Te*. *tilletti* (OMNH 58340). Although the mesiolingual margin is sharp, it is not emarginated basally by the lingually expanded ridge extending from the cingulum (mesial bounding ridge sensu Bell et al., [27]) evident on some rhabdodontomorphs [39]. In this respect, the dentary teeth are similar to *Qantassaurus* [92] and *Te*. *tilletti* (OMNH 58340). A more shallow depression is present on the distal face, emarginated by a defined distal bounding ridge (Fig 12I) as *Te*. *tilletti* (OMNH 58340) and rhabdodontomorphs. All dentary teeth bear a pronounced primary ridge that is relatively centered on the crown (Fig 12F and 12H) (as in *Weewarrasaurus* [27] and in contrast to *Zalmoxes* [61] where it is slightly offset), creating a triangular lingual face in cross-section. Four to six well-developed secondary ridges are variably present on each side of the primary ridge (Fig 12F), four of which typically extend from the apex to the crown base; this is similar to *Te*. *tilletti* which has two to five extensive ridges per side [60]. Rhabdodontids generally bear a greater number of secondary ridges on either side of the primary ridge (e.g., 5–13; [39, 79]). Slight ridges are also present on the labial face of the dentary crowns (Fig 12G) as in *Te*. *tilletti* (OMNH 58340) and rhabdodontomorphs, although they are less prominent. As in rhabdodontids, wear initiated on the distal aspect of the dentary crowns, yet it is unclear, due to preservation, if there were distinct mesial and distal facets, as observed in that clade [39, 61]. Enamel thickness on the labial surface (79.3 μm) is four times that of the lingual surface (19.8 μm) (Fig 13).

#### Vertebrae

Representative cervical, dorsal, sacral, and caudal vertebrae are preserved; however, the majority of vertebrae recovered were pieced together from surface collected fragments and are incomplete. In addition, many vertebral fragments are present.

#### Cervical vertebrae

**Fig 14:**
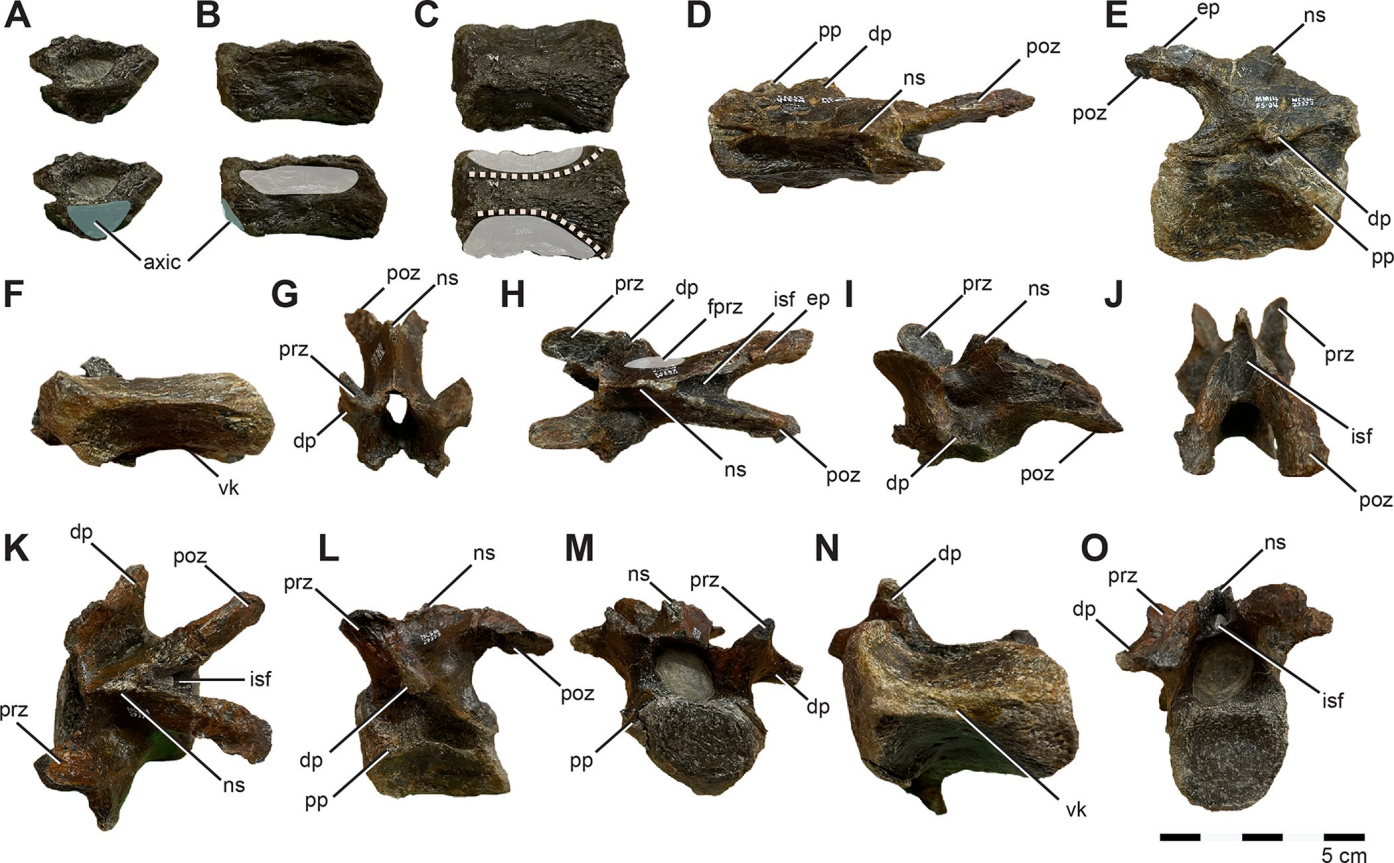
Cervical vertebrae of *Iani smithi* (NCSM 29373). Axis in (A) cranial, (B) left lateral, and (C) ventral views; C3? in (D) dorsal, (E) right lateral, and (F) ventral views; C4? in (G) cranial, (H) dorsal, (I) left lateral, and (J) caudal views; Caudal cervical (C9?) in (K) dorsal, (L) left lateral, (M) cranial, (N) ventral, and (O) caudal views. Abbreviations: axis, axis intercentrum; dp, diapophysis; ep, epipophysis; isf, interspinous fossa, ns, neural spine; pp, parapophysis; pop, postzygapophysis; prz, prezygapophysis; vk, ventral keel. Color annotation: white, depressions/fossae/grooves; green, articular surfaces; blue circles, foramina; light blue lines, marginal contours; peach dashed lines, ridges/internal contours. Scale bar 5 cm.

Portions of at least seven cervical vertebrae are preserved with *Iani*. All bear transversely constricted centra with a pronounced ventral keel (Fig 14). The atlas is not present; however, an isolated axis centrum apparently bearing a fused intercentrum is preserved (Fig 14A and 14B). The axis of *Iani* is compressed dorsoventrally, somewhat eroded ventrally, and approximately 150% longer than wide (Fig 14A–14C). The cranial articular facet bears a deep furrow for reception of the missing odontoid, ventral to this the fused atlas intercentrum forms a sharp, cranially projecting lip on the cranioventral margin as in *Te*. *tilletti*, *Hypsilophodon*, *Z*. *robustus*, and *Rhabdodon* [61, 65, 69, 95]. It is only poorly developed as in *Z*. *robustus* and *Rhabdodon* [61, 65] and does not create a pronounced ventral lip as observed on *Te*. *tilletti* [95]. The caudal articular facet is concave. In ventral view, the axis centrum is medially constricted and widens toward the caudal articular facet (Fig 14C), in contrast to *Hypsilophodon* where the ventral keel widens cranially [69]. Cervicals three and four are represented by a nearly complete, laterally compressed vertebra (Fig 14D–14F), and an undistorted isolated neural arch (Fig 14G–14J). The neural arch on C3 is missing the prezygapophyses and the neural spine is triangular and formed by a gently sloping ridge arising between the bases of the prezygapophyses. The fourth cervical bears a centered and craniocaudally restricted neural spine that rises abruptly from the space between prezygapophyses. In dorsal view, pre- and postzygapophyses are relatively parallel to the main axis of the arch on each vertebra (Fig 14D and 14H), which is relatively elongate (in contrast to the cranial cervicals of *Rhabdodon* [65] and *Z*. *robustus* [61], which are blunter and more x-shaped in dorsal view. Postzygapophyses on C4 bear pronounced epipophyses confluent with a dorsal ridge along the postzygapophyses as in *Te*. *tilletti* (OMNH 58340) and the mid-cervicals of *Rhabdodon* (CM- 441) [65] and *Z*. *robustus* [61]. Due to poor preservation, it is difficult to determine how extensively they were distributed across the cervical series. On the cervical neural arches distal to C3, the combination of a cranially migrated neural spine with strong dorsal ridges on the postzygapophyses creates a deep interpostzygapophyseal v-shaped channel (interspinous fossa) in dorsal view (Fig 14D and 14H). In dorsal view, the neural arch of C4 possesses deep, laterally facing, axially oriented, slit-like furrows for reception of the postzygapophysis (Fig 14H). Cervical centrum 3 is amphicoelous; however caudal to this all cervical centra are platy- to slightly opisthocoelous as in *Te*. *tilletti* [95]. Cervical centra three and four would have been approximately twice as long as wide.

An additional two vertebrae represent the mid-cervical series (C6–7) and possess partially fused neural arches. Mid-cervical centra and neural arches reduce in craniocaudal length toward the trunk region, becoming more robust and their postzygapophysis begin to arch dorsally and flare laterally as in *Te*. *tilletti* [95]. A well-developed lateral ridge extends confluently from the lateral margin of the postzygapophyseal facet along the lateral postzygapophysis on C6 as in *Te*. *tilletti* (MOR 682). In the same order, the neural spine begins to migrate caudally. In caudal view, the dorsalmost margin of the neural canal is pinched by fine medial shelves projecting from the dorsomedial walls of the neural canal. This creates a bilobate neural canal in C4–6. However, in the caudal cervicals, these shelves become more robust and eventually contact creating an interspinous fossa floored ventrally by a well-developed intrapostzygapophyseal lamina. The caudal cervicals are the most robust and are represented by one well-preserved vertebra (Fig 14K–14O) and a fragment of a neural arch (C9-11). Centra are more equidimensional (~1–1.25 length-to-width ratio). The cranial and caudal articular facets do not appear offset as reported for *M*. *vorosi* and *Zalmoxes* [39].

#### Dorsal vertebrae

**Fig 15:**
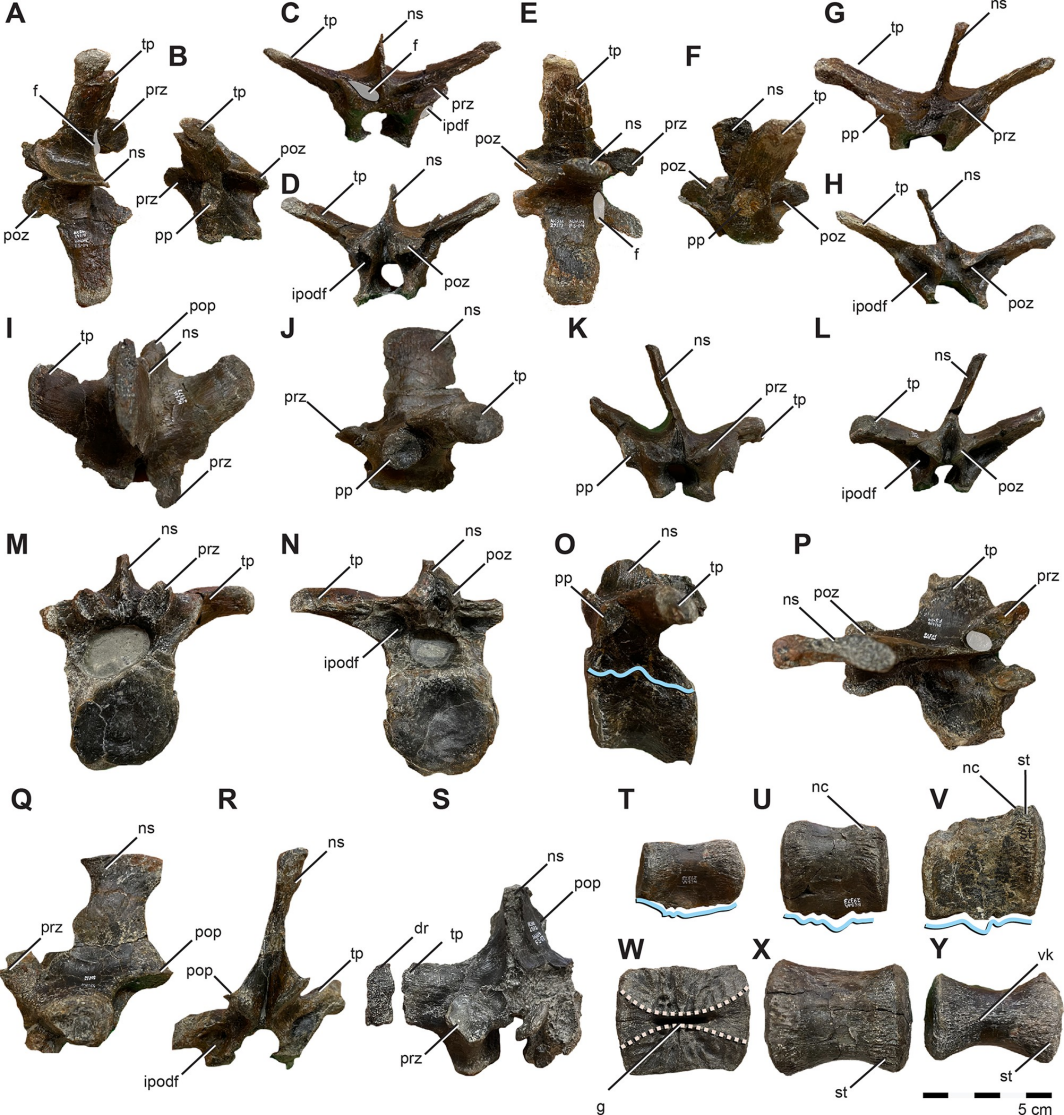
Dorsal vertebrae of *Iani smithi* (NCSM 29373). D1? in (A) dorsal, (B) left lateral, and (C) cranial, and (D) caudal views; D2? In (E) dorsal, and (F) left lateral, (G) cranial, and (H) caudal views; D9/10? in (I) dorsal, (J) left lateral, (K) cranial, and (L) caudal views; D11/12? in (M) cranial, (N) caudal, and (O) left lateral views; D15? in (P) dorsal, (Q), left lateral, and (R) caudal views; (S) D16? in cranial view. Isolated dorsal centra in (T, U, V) lateral, (W) dorsal, and (X, Y) ventral views. Abbreviations: dr, distalmost dorsal rib; f, slit-like fossa caudal to prezygapophyses; g, groove; ipdf, infraprediapophyseal fossa; ipodf, infrapostdiapophyseal fossae; nc, notochordal tubercle; ns, neural spine; pop, postzygapophysis; prz, prezygapophysis; st, striations; tp, transverse process; vk, ventral keel. Color annotation: white, depressions/fossae/grooves; green, articular surfaces; blue circles, foramina; light blue lines, marginal contours; peach dashed lines, ridges/internal contours. Scale bar 5 cm.

At least eight unfused dorsal neural arches and two partially fused dorsal vertebrae are preserved in variable states of damage and distortion. Cranial dorsal vertebrae are craniocaudally short and relatively gracile (Fig 15A–15H). The neural arch of D1 and D2 bear infraprezygadiapophyseal fossae (Fig 15C), although they are nearly absent in the latter. The prezygapophyses on D1 project from the cranial aspect of the transverse process (Fig 15B and 15F). Caudal to this they rapidly transition to distinctly projecting facets. They are widely separated and somewhat laterally splayed in the cranial dorsals and migrate mesially toward the sacrum, narrowing in transverse width and reorienting closer to the midline. Cranialmost dorsals (D1–D3) also bear deep slit-like fossa on the cranial neural arch, just caudal to the prezygapophyses (Fig 15A, 15C and 15E) as in *Te*. *tilletti* (OMNH 58340). These facets would have received and caudally braced the postzygapophyses when maximally dorsiflexing the neck. The neural spine of D1 appears to have remained subtriangular as in the cervical series (Fig 15A–15D); however, we cannot rule out preservational damage. Caudal to this, the neural spines are rectangular, transitioning from craniocaudally narrow and shorter to wider and taller caudally. They remain relatively short throughout the series as in *Zalmoxes* [61, 79] and *Tenontosaurus* [95], in contrast to the taller spines of *Rhabdodon* [65]. Infrapostdiapophyseal fossae are present throughout the entire dorsal series and transition from subtriangular fossae cranially, to deeper, more medially subcircular pits caudally (Fig 15D, 15H, 15L, 15N and 15S). Transverse processes are dorsally elevated and strap-like cranially, and transition caudally to stouter, more robust features. One caudal dorsal (D11 or D12?) bears a somewhat pendant transverse process (Fig 15M). Caudal to this, two dorsal neural (D15? & D16?) arches bear blunt transverse processes with concave termini indicative of single-headed, reduced dorsal ribs (Fig 15Q, 15R and 17L). The caudalmost dorsal neural arch (D16?) preserves an unfused, blunt cylindrical rib that attaches to the transverse process (Fig 15S). The neural spine on the caudalmost dorsals widens ventrally into a fan-shaped structure with at least a caudal hook (the cranial portion is damaged) (Fig 15R).

The caudal dorsal series (~>D10) bears a centrum to neural arch articulation whereby two pointed projections extend from the constricted portion of the centra to insert into the neural arch (Fig 15U and 15V). This trait is also observed on thescelosaurids (UT130831-1). On *Iani*, this condition appears to become more pronounced caudally. In more cranial dorsals, this manifests as more of a gentle dorsal convexity observable in lateral view (Fig 15T), rather than a distinct projection; nonetheless, the neural arch-centrum articulation in all dorsals is dorsally arched in the middle to some degree. This articular prong does not appear to be present on *Mochlodon* [39] or *Zalmoxes* [79], but may be present on the caudalmost dorsals of *Rhabdodon* [65] and *Tenontosaurus* (OMNH 58340); however, it is difficult to identify in fused vertebrae overall and warrants further investigation. Additional features of interest regarding the neural arch/centrum articulation include subtriangular articulation surfaces that project medially to variable degrees (Fig 15W) but generally extend inward toward the midline, nearly contacting one another and excluding the centrum from participating in the floor of the neural canal (Fig 15W). The nature of this articulation extends from the dorsal series through the sacral series; however, in the dorsal series, a deep slit-like groove penetrates the dorsal centra (Fig 15W); whereas on the sacrals this feature manifests as a central pit on the dorsal surface of the centra (see sacral vertebrae below).

A partial centrum (cranial articular facet) is preserved on? D3; it is taller than wide and heart-shaped. Caudal to this, the centra became more spool-shaped and robust. All are amphicoelous and bear deep, rugose, craniocaudal striations along the ventral margins of the cranial and caudal articular facets (Fig 15V, 15X and 15Y) not exhibited by *Mochlodon* [39] or *Z*. *shqiperorum* [79] yet notably present on thescelosaurids. A notochordal tubercle is present on some centra (Fig 15O, 15U and 15V) as in *Z*. *robustus* [61]. Two additional isolated centra likely belong to the dorsal series because they bear ventral rugosities (not present on cervicals, sacrals, or caudals) and lack parapophyses, chevron facets, and evidence of fusion with adjacent centra; however, they are elongate and their position cannot be matched to the existing dorsal neural arches, which all appear substantially shorter.

#### Sacral vertebrae

**Fig 16:**
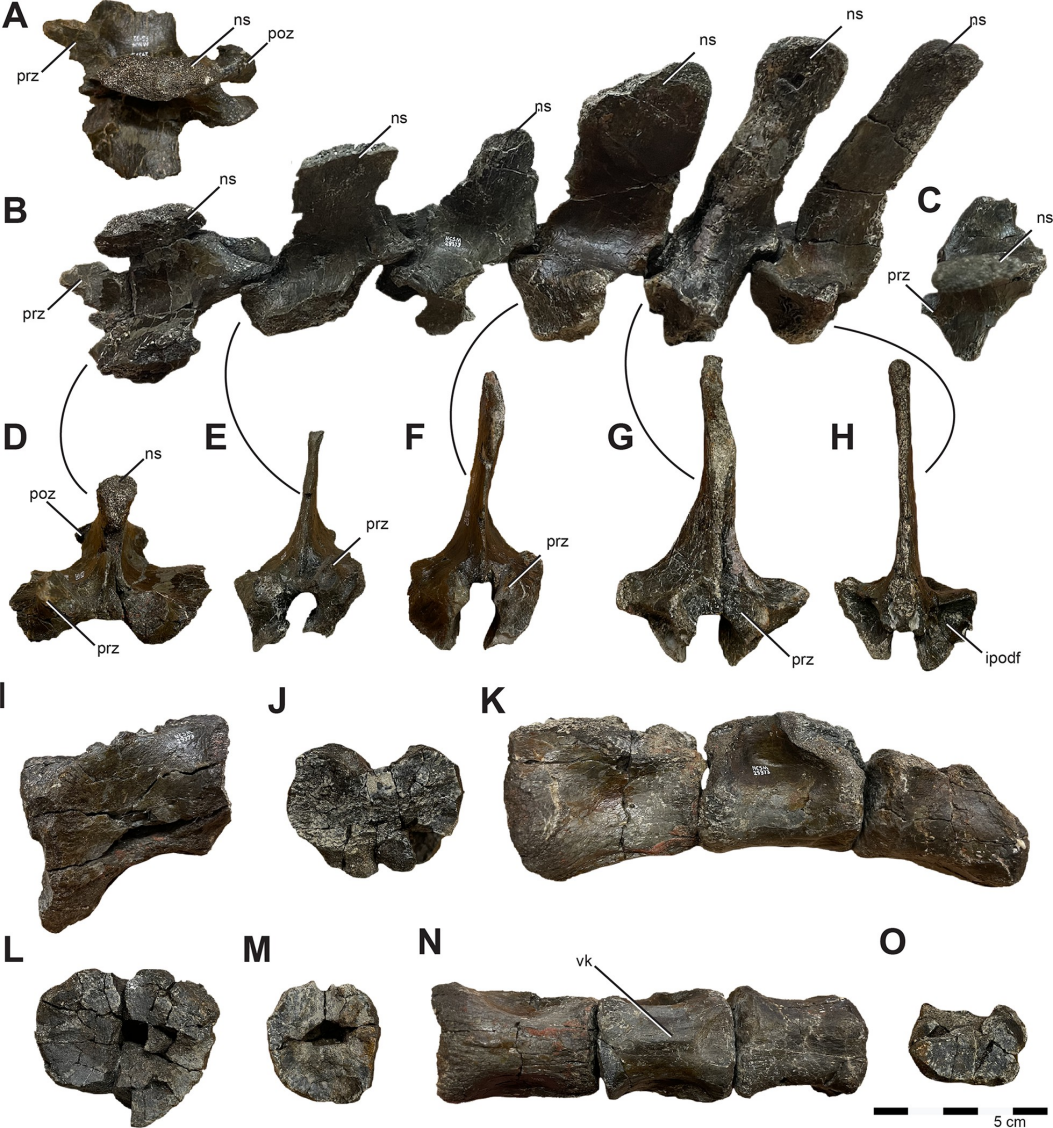
Sacral vertebrae of *Iani smithi* (NCSM 29373). Neural arch of S1 in (A) dorsal and (D) cranial views; (B) sacral neural arches 1–6 in left lateral view; (C) neural arch of S6 in caudal view; (E) neural arch of S2 in cranial view; (F) neural arch of S4 in cranial view; (G) neural arch of S5 in cranial view; (H) neural arch of S4 in caudal view; Sacral centrum S1? in (I) left lateral, (J) caudal, and (L) cranial views; Sacral centra S2?–5? in (K) left lateral and (N) ventral views; (M) sacral centrum S2? In cranial view; (O) sacral centrum S5? in caudal view. Abbreviations: ipodf, infrapostdiapophyseal fossae; ns, neural spine; pop, postzygapophysis; prz, prezygapophysis; vk, ventral keel. Color annotation: white, depressions/fossae/grooves; green, articular surfaces; blue circles, foramina; light blue lines, marginal contours; peach dashed lines, ridges/internal contours. Scale bar 5 cm.

The sacrum is entirely unfused. Six sacral neural arches (Fig 16A–16H) and four isolated sacral centra (Fig 16I–16O) are preserved. Sacral neural arches transition from craniocaudally long with shorter, caudally trending neural spines, to craniocaudally compressed with taller, straight neural spines. Mid-sacral neural arches bear infrapostdiapophyseal fossae (Fig 16H) and have blunt, rudimentary pre- and postzygapophyses (Fig 16B, 16D–16G) that are inset into the arch, producing articular fossae for the reception of corresponding neural arches. All centra are asymmetrical with the caudal articular facet being reduced and oriented at an angle relative to the cranial facet, producing a curved sacral body (Fig 16I and 16K). Centra vary from bearing a low-profile, rounded, and nearly indistinct ventral keel in the mid-sacrum to a flattened, and slightly grooved ventral profile caudally (Fig 16N) unlike the ventrally grooved sacrum of *Te*. *tilletti* [95]. Cranial to mid-sacral centra bear bilateral concavities (Fig 16N) and, in dorsal view, a pit excavates the ventral floor of the neural canal. These features are not present in the caudalmost sacral centra.

#### Caudal vertebrae

**Fig 17:**
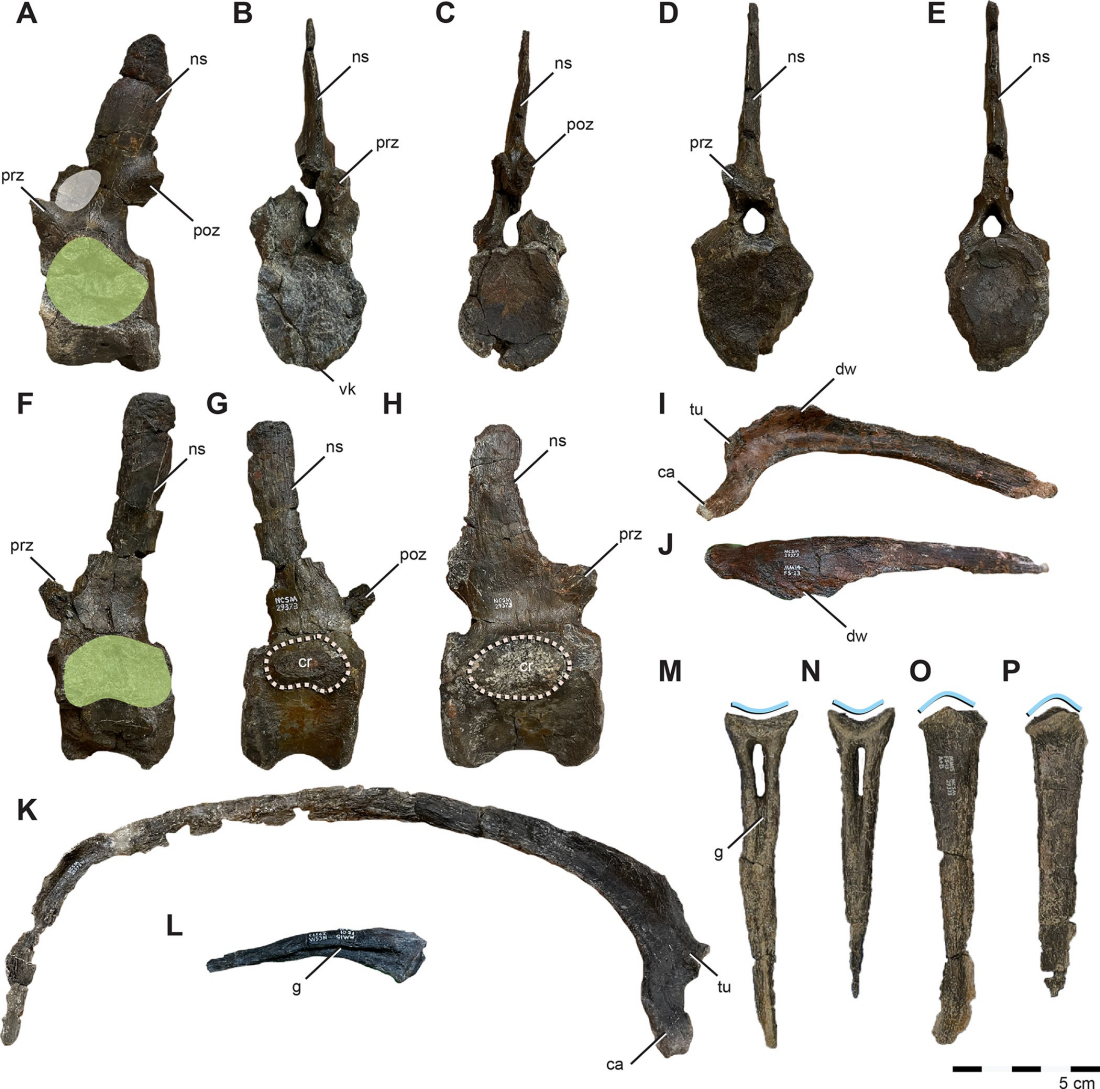
Caudal vertebrae, ribs, and haemal arches of *Iani smithi* (NCSM 29373). C1? in (A) left lateral, (B) cranial, and (C) caudal view. C5? in (D) cranial, (E) caudal, (F) left lateral, and (G) right lateral views. C7? In (H) left lateral view. (I) left dorsal rib in caudal view. Right dorsal rib in (K) caudal and (J) dorsal views. Caudal dorsal rib in (L) caudal view. Chevrons in (M, N) cranial and (O, P) lateral views. Abbreviations: ca, capitulum; cr, caudal rib; dw, dorsal wing; g, groove; ns, neural spine; pop, postzygapophysis; prz, prezygapophysis; tu, tuberculum. Color annotation: white, depressions/fossae/grooves; green, articular surfaces; blue circles, foramina; light blue lines, marginal contours; peach dashed lines, ridges/internal contours. Scale bar 5 cm.

Caudals 1–7 are preserved. Prezygapophyses are steeply inclined (Fig 17A and 17F). The proximalmost two caudal neural spines are straight and trend caudodorsally (Fig 17A), beyond this a single neural spine is craniocaudally narrower and bears the slightest cranial convexity (Fig 17F), as opposed to the strong convexity exhibited by *Tenontosaurus* [95]. The first three caudals bear bilateral depressions at the base of the neural spine caudal to the prezygapophyses (Fig 17A). All preserved centra are taller than wide (Fig 17C and 17E) and platycoelous as in *Te*. *tilletti* [95] (and less so in *Mochlodon* [39]) and in contrast to the proximal caudal centra of *Z*. *robustus* and *Rhabdodon*, which are transversely wider than dorsoventrally tall [61, 65]. Oddly, caudal ribs are unfused on the left lateral side of all but one (the distalmost) caudal and fused contralaterally on all but Cd1 (on which both are unfused). The bases of the caudal ribs are proportionally large and subcircular on the proximalmost caudals (greater than 50% the dorsoventral height of the centrum itself) and span the centrum/neural arch boundary (Fig 17A, 17F–17H). A weak rounded ventral keel is present on the proximalmost caudal centra (Fig 17B); mid-caudals (at least to position Cd8) do not bear a ventral groove.

#### Hemal arches

Portions of at least three proximal chevrons are preserved. They are elongate and subequal in length with the tallest complete proximal caudal vertebra (Fig 17M). The proximal facet is a single articular surface that is v-shaped in proximal view (Fig 17M and 17N), and dorsally vaulted in lateral view (Fig 17O and 17P). A deep axial groove divides the cranial and caudal aspects (Fig 17M and 17N) unlike *Rhabdodon* [65]. None of the preserved chevrons fan craniocaudally at their ventralmost extent; however, this feature varies positionally on *Te*. *tilletti* [95]. They are axially compressed only at their distalmost extent (no more than the ventral third on the best-preserved exemplar (Fig 17M). The hemal canal is a highly compressed oval (Fig 17N and 17O) as opposed to the more rounded canal in *Rhabdodon* [65].

#### Ribs

At least 14 dorsal ribs are represented including many with distinct tubercula and capitula (Fig 17I and 17K) and at least one single-headed exemplar (Fig 17L). Ribs are robust and strongly bowed, more so than reported for rhabdodontomorphs [65], and bear a distinct, subtriangular, caudally flaring dorsal wing extending from the tuberculum that roofs an extensive concavity in this region (Fig 17I, 17J). This feature is not present in *Rhabdodon* [65], *Z*. *robustus* [61], or *Te*. *tilletti* (OMNH 67502). Isolated sacral ribs are preserved.

#### Scapula

**Fig 18:**
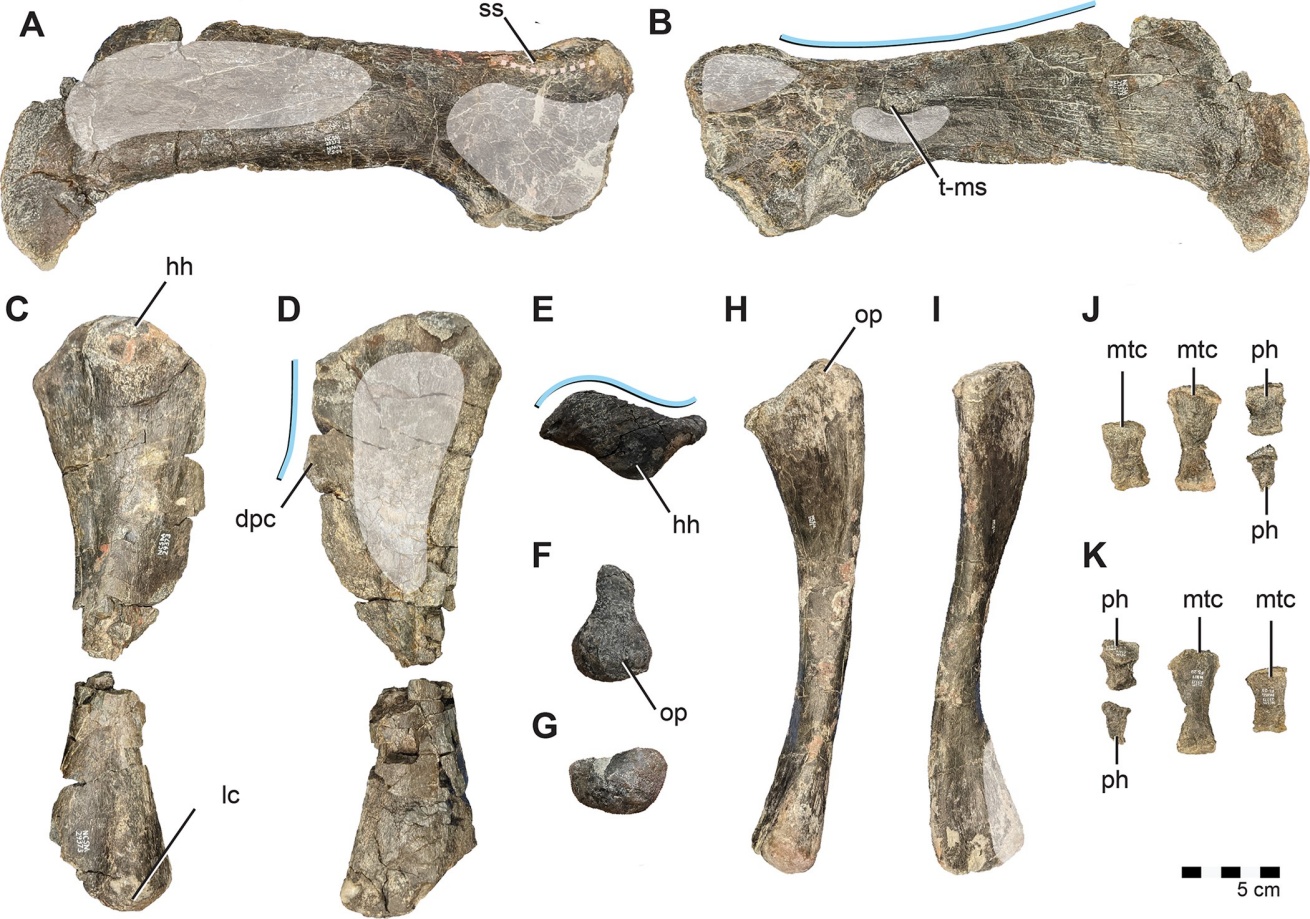
Pectoral girdle and forelimb of *Iani smithi* (NCSM 29373). Right scapula in (A) lateral and (B) medial views. Right humerus in (C) caudal, (D) cranial, and (E) proximal views. Right ulna in (F) proximal, (G) distal, (H) medial, and (I) cranial views. Metacarpals and manual phalanges in (J) extensor, and (K) flexor views. Abbreviations: dpc, deltopectoral crest; dr, deltoid ridge; hh, humeral head; lc, lateral condyle; mtc, metacarpal; op, olecranon process; ph, phalanx; t-ms, tuberosity for the M. subscapularis. Color annotation: white, depressions/fossae/grooves; green, articular surfaces; blue circles, foramina; light blue lines, marginal contours; peach dashed lines, ridges/internal contours. Scale bar 5 cm.

Right and left scapulae are preserved. They are similar in overall form and proportion to those of *Te*. *tilletti* (MOR 682) and one specimen of *Z*. *robustus* (BMNH R.381) [61] and are stouter and more robust than the slender, strap-like scapulae of *Z*. *shqiperorum*, *Z*. *robustus* (BMNH R.3810), or *M*. *vorosi* [39, 79], yet more elongate than those of *Rhabdodon* [65]. The lateral face of the proximal region is dominated by a subtriangular deltoid fossa bounded dorsally by a recognizable, but relatively poorly developed, deltoid ridge (Fig 18A). The acromion process is in line with the dorsal margin of the scapula (Fig 18A) and not rotated medially as in more derived ornithopods. In medial view, the acromion exhibits a pronounced depression, buttressed by a well-developed, robust caudoventral ridge (Fig 18B). Caudal to this there is a prominent tuberosity for the insertion of M. subscapularis at the base of the scapular blade (Fig 18B), as in *Oryctodromeus* and *Uteodon* [96]. This tuberosity appears to be absent on *Mochlodon* [39], *Zalmoxes* [79] (although a homologous ridge on the medial scapula closer to the distal end is noted as present on *Z*. *robustus* and *Z*. *shqiperorum* by Brusatte et al., [97], and is not described for *Hypsilophodon* [69], but may be present on *Rhabdodon* (CM-611) [65: Fig 4.22]. Ventral to this tuberosity is a small sulcus in the same location as described by Andrzejewski [64] for *Convolosaurus* (Fig 18B).

The dorsal margin of the blade is slightly concave dorsally as opposed to the relatively straight dorsal margin reported on *Te*. *tilletti* [95] (Fig 18A and 18B); however, Forster [95] reports that this feature varies ontogenetically with the concave condition of *Iani* representative of *Te*. *tilletti* juveniles. The distal blade of *Iani* possesses a substantial ventral hook that is similar to that of *Rhabdodon* [65] and *Hypsilophodon* [69], and more extreme than observed on *Te*. *tilletti*. A ventrally hooked scapular blade is a feature more pronounced in early-diverging ornithopods [98] (Fig 18A and 18B). Such a hook is not known on *Z*. *shqiperorum* or *M*. *vorosi* [39, 79] (although the shape of the scapular blade on reported specimens of *Z*. *robustus* [61] appears variable and is noted to be variable on *Te*. *tilletti* [95].

#### Humerus

The right humerus preserves a nearly complete proximal portion (missing most of the deltopectoral crest) and the lateral aspect of the distal portion (Fig 18C and 18D). The humeral head is large, comprising just over 50% of the transverse width of the proximal humerus (Fig 18E). It is dorsally elevated in craniocaudal view giving the proximal humerus a vaulted appearance as in *Te*. *dossi*, *Convolosaurus*, and *Z*. *robustus* [61] (Fig 18C). Later diverging ornithopods such as *Camptosaurus* [66] and *Dryosaurus* (e.g., YPM VP 1876) [66] have a squared-off proximal region. The humeral head expands craniomedially giving the cranial margin of the proximal humerus a sigmoidal outline in proximal view (Fig 18E). The deltopectoral crest was robust but is only partially preserved. Nonetheless, the preserved margin indicates that the region between the apex of the deltopectoral crest and proximolateralmost margin of the humerus was concave as in rhabdodontomorphs [37] and *Tenontosaurus* [62, 95] (Fig 18D). The proximal humerus is bowed medially.

#### Ulna

A relatively undistorted, well-preserved right ulna was recovered. The proximal end is robust and bears a bulbous olecranon process that, in contrast to *Te*. *tilletti*, *Z*. *robustus*, *Rhabdodon*, and *M*. *vorosi* [39, 61, 65, 95] nearly lacks a proximal expansion, instead lying almost inline with the cranialmost terminus of the cranial coronoid process as in *Dryosaurus* [99] (Fig 18H). The cranial coronoid process is only moderately projected and is transversely wide with a blunt terminus (Fig 18H and 18I). There is little to no development of a lateral coronoid process to brace the radius, unlike *Te*. *tilletti* [95] (Fig 18F). In cranial view, the proximal aspect is bowed laterally and the distal is bowed strongly medially (Fig 18I). In lateral view, the ulna is caudally bowed. The ulnar shaft is craniocaudally taller than mediolaterally wide in cross-section and subrectangular throughout most of its length, although it transitions to being transversely wider than tall just proximal to the distal end. At this point the ulnar shaft bends strongly laterally, producing an overall sigmoid shape in cranial view. The lateral bend is similar to, yet more pronounced than that observed on *Rhabdodon* [65], and *Te*. *tilletti* [95]. The distal ulna is hemispherical in form with a flat cranial face and convex caudal margin (Fig 18G). As in *Te*. *tilletti*, the axis of the distal end is rotated laterally [95].

#### Radius

A fragment of a proximal radius is preserved along with a highly eroded partial shaft missing both ends that likely represents the radial diaphysis. The proximal facet is concave, suboval, and compressed, and generally similar in form to *Te*. *telletti* [95].

#### Manus

Four manual elements are severely dorsoventrally compressed and poorly preserved, making precise identifications difficult. This is compounded by the fact that few early-diverging ornithopods (and rhabdodontomorphs specifically) preserve a good record of the manus, early digital evolution of the ornithopod manus is complex and poorly studied [100], and the best preserved manus of a closely related taxon (*Te*. *tilletti*) is highly autapomorphic [100].

Two elements were found associated and likely represent metacarpals (Fig 18J and 18K). One of these is elongate and likely represents MC II or III. It bears subequally wide proximal and distal articular surfaces in contrast to a similarly positioned metacarpal described for *Rhabdodon* [65], exhibits collateral ligament pits bilaterally, and apparently had a subtriangular proximal articular end. The other, although shorter, retains a convex proximal articular surface suggestive of a metacarpal and could represent MC I, IV, or V. It also bears a subtriangular proximal surface and collateral ligament pits. Two additional elements were found articulated and represent penultimate and ultimate (ungual) phalanges of digits 1, 2, or 3 (Fig 18J and 18K). The penultimate phalanx is wider proximally and lacks any evidence of collateral ligament pits. The ungual is relatively elongate as in *Te*. *tilletti* [100] and *Hypsilophodon* [69], in contrast to the spade-shaped ungual reported for *Rhabdodon* [65].

#### Ischium

**Fig 19:**
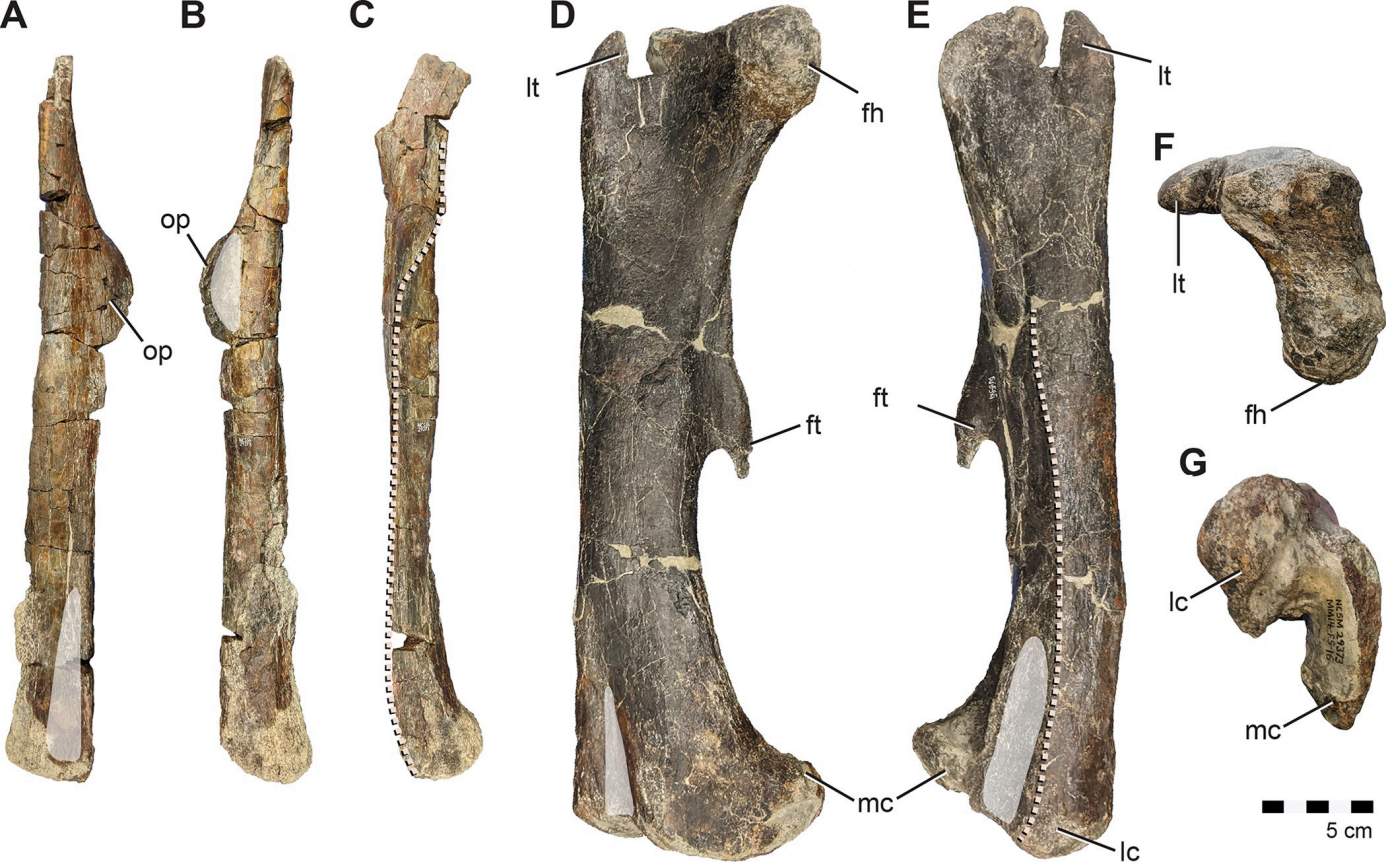
Pelvic girdle and hind limb of *Iani smithi* (NCSM 29373). Right ischium in (A) dorsal, (B) ventral, and (C) medial views. Right femur in (D) medial, (E) lateral, (F) proximal, and (G), distal views. Abbreviations: fh, femoral head; ft, fourth trochanter; lc, lateral condyle; mc, medial condyle; op, obturator process. Color annotation: white, depressions/fossae/grooves; green, articular surfaces; blue circles, foramina; light blue lines, marginal contours; peach dashed lines, ridges/internal contours. Scale bar 10 cm.

*Iani* preserves the shaft and distal end of a left ischium (Fig 19A–19C). The shaft is subtriangular in cross-section proximally, with a flattened lateral surface bearing a well-defined groove and pointed ventromedial surface leading into the obturator process, as in *Hypsilophodon* [69]. Distally, the shaft flattens. This condition is distinct from that of *Tenontosaurus*, which has a flattened and strap-like ischial shaft throughout [95]. Although generally straight shafted, there is a distinct twist to the distal ischial shaft on *Iani*, which transitions from having a dorsoventrally long axis proximally to a mediolaterally long axis distally, as in *Te*. *dossi* [62], *Jeholosaurus* [101], and *Hypsilophodon* [69]. On *Hypsilophodon*, this twist is approximately 45 degrees; whereas on *Iani*, the axes are nearly perpendicular. A twisted ischial shaft is unreported in *Te*. *tilletti* [95] and also appears absent on *Rhabdodon* [65] and *Zalmoxes* [61, 79]. The obturator process is positioned proximally, originating where the proximal end of the ischium begins expanding for articulation with the ilium (Fig 19C), as in *Dryosaurus* [99] and *Rhabdodon* [65]. It is more distally positioned on *Te*. *tilletti* [95]. An obturator process is absent on *Zalmoxes* [61, 79]. *Iani* exhibits an asymmetrical ventral boot.

#### Femur

A right femur with erosion to the region between the greater and lesser trochanters is preserved (Fig 19D and 19E); it is otherwise in good condition. Overall, it is relatively straight to slightly sigmoid, with a lateral bow proximally and a medial bow distally, similar to *Dryosaurus* [99]. The femoral head is robust and spherical, extending on a thickened neck (Fig 19F), with a slight groove for the foveal ligament along the caudal face as in *Te*. *tilletti* [95]. The fossa trochanteris is shallow as in the Vegagete ornithopod [37]. In dorsal view, the proximal femur is not symmetrical (neither wedge-shaped as in *Hypsilophodon* [69], mushroom-shaped as in *Dryosaurus* [99], or hourglass-shaped as in *Te*. *tilletti* [95] and to a lesser extent *Burianosaurus* [34], but rather concave cranially and straight caudally, bearing a greater trochanter that is substantially craniocaudally wider than the femoral head and neck (more similar to *M*. *vorosi*; [39]) (Fig 19F). In craniocaudal and medial views, there is a moderate depression between the femoral head and greater trochanter (Fig 19D) as in *Hypsilophodon* [69], *Burianosaurus* [34], *M*. *vorosi* [39], and *Dryosaurus* [99] and unlike the deep depression of *Z*. *shqiperorum* [79], or the proximally straight condition of *Rhabdodon* [65]. The lesser trochanter is columnar and extends as far proximally as the greater trochanter (Fig 19D) as in *Hypsilophodon* [69], *M*. *vorosi* [39], *Te*. *tilletti* [95], and *Dryosaurus* [99] in contrast to *Z*. *shqiperorum* [79] where the lesser trochanter terminates distal to the greater trochanter. It is separated from the greater trochanter by a cleft as in *Z*. *shqiperorum* and *M*. *vorosi* [39, 79], as opposed to the shallow depression of *Rhabdodon* [65]. In contrast to the secondarily reduced condition of rhabdodontomorphs and *Burianosaurus* [34], the fourth trochanter retains a well-developed, pendant form (Fig 19D). Its distalmost extent terminates at approximately 55% the length of the femur. In distal view, the medial condyle is transversely compressed and craniocaudally more elongate than the lateral condyle (Fig 19G) as in *Te*. *tilletti* [95] and *Dryosaurus* [99]. In medial view, the medial condyle is flush with the shaft cranially, yet extends a great distance caudally as in *Burianosaurus* [34]. There is a narrow, relatively shallow extensor groove on the cranial face of the distal aspect (Fig 19D).

## Phylogenetic results

**Fig 20:**
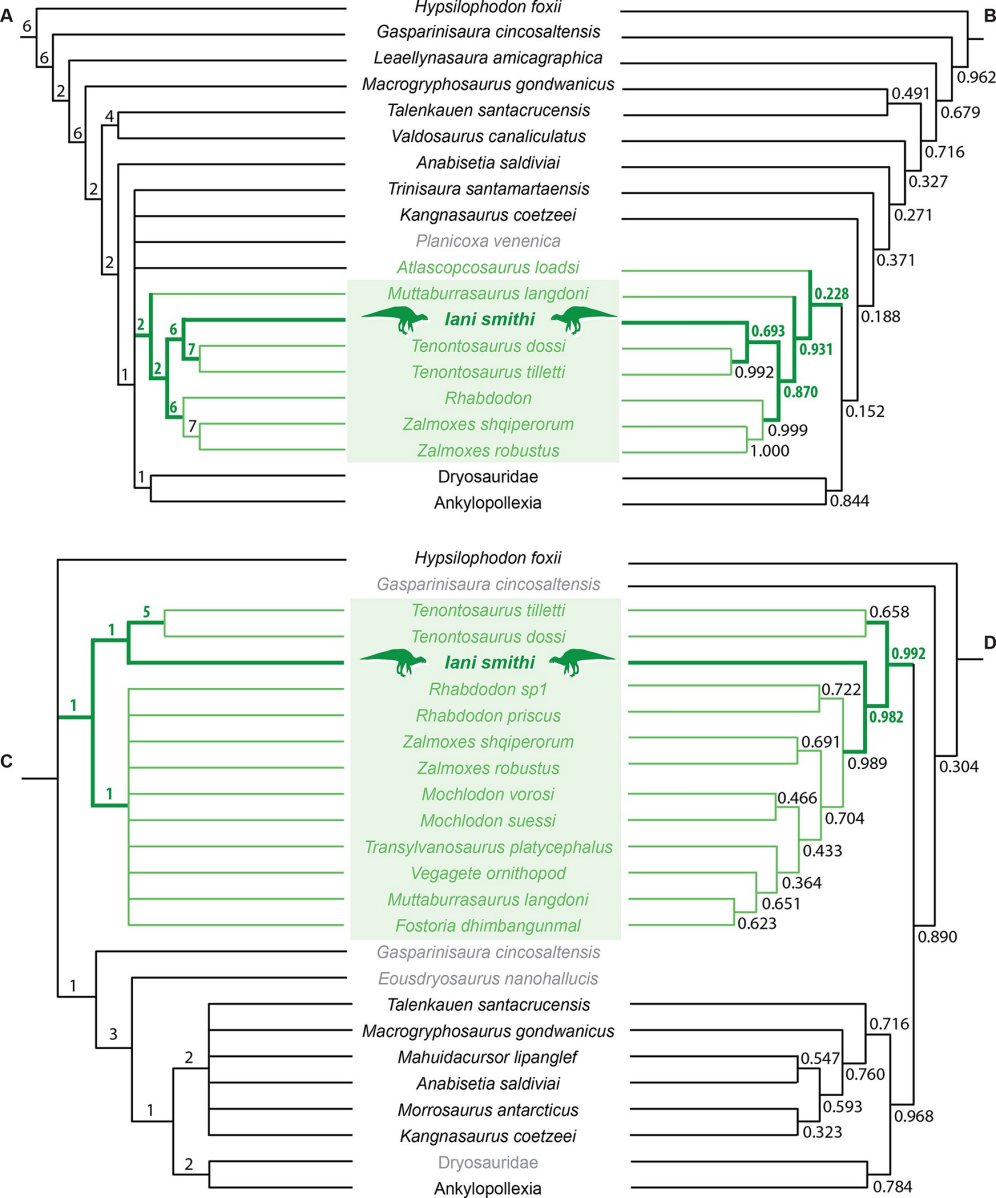
Hypothesized evolutionary relationships of *Iani smithi*. Strict consensus tree of the Poole [32] matrix using (A) maximum parsimony optimality, and (B) Bayesian inference, and strict consensus tree of the Dieudonné et al., [33] matrix using (C) maximum parsimony optimality, and (D) Bayesian inference. Rhabdodontomorph taxa in green. Bremer support values (left) and posterior probabilities (right) shown. Grey names reflect taxa outside the illustrated clade for a particular analysis, or in the case of Dryosauridae (an ad hoc combined OTU with variable composition using different analytical approaches).

We tested the evolutionary relationships of *Iani* using three recent phylogenetic matrices focusing on early-diverging neornithischians, rhabdodontomorphs, and early-diverging iguanodontians (Barta and Norell [31]; Dieudonné [33], and Poole [32], respectively). Our primary findings—that *Iani* is closely related to the genus *Tenontosaurus* and rhabdodontids—are stable regardless of matrix chosen or analytical approach employed. Recovery of *Iani* as a member of Rhabdodontomorpha (sensu Madzia et al., [28]) is consistent in all analyses with the exception of the Barta and Norell [31] matrix, which does not contradict this hypothesis, but rather is too poorly resolved to be informative. The Barta and Norell [31] matrix is based on Boyd [35] and is targeted at resolving relationships among thescelosaurids. It contains the poorest representation of rhabdodontomorph taxa and traits (we note that this region of the tree in Boyd’s [35] analysis was also unresolved), thus we do not discuss it further here. In our other analyses using maximum parsimony as an optimality criterion, *Iani* is posited as the earliest diverging member of an exclusive subclade with *Te*. *dossi* and *Te*. *tilletti* from the Lower Cretaceous of North America, which together comprise the sister taxon to rhabdodontids (Fig 20A and 20C). The same topology is recovered using the Poole [32] matrix with Bayesian inference (Fig 20B). Bayesian analysis of the Dieudonné [33] matrix produces a slightly different hypothesis whereby *Iani* is recovered as an evolutionary step between the divergence of *Tenontosaurus* and Rhabdodontomorphs including the Australian taxa *Muttaburrasaurus* and *Fostoria*, and the unnamed, Early Cretaceous “Vegagete ornithopod” (Fig 20D). In no analysis do we recover *Iani* as a rhabdodontid (sensu Madzia et al., [28]), and in no analysis do we recover *Iani* outside Rhabdodontomorpha. In sum, using currently available phylogenetic matrices, we find analytical support for *Iani* as a rhabdodontomorph, and as a transitional taxon linking *Te*. *dossi* and *Te*. *tilletti* from the Lower Cretaceous of North America with Late Cretaceous rhabdodontomorphs more generally.

## Discussion

### Rhabdodontomorpha: A clade of unstable composition

The early evolution of Ornithopoda and its divergence from other neornithischian clades remains one of the most contentious and poorly resolved areas of Ornithischian research (e.g., [28, 102]. *Tenontosaurus* has long featured as a transitional representative between “hypsilophodonts” and later diverging ornithopods (dryomorphs) (e.g., [90, 102–104]) a position it later came to share with a variety of early-diverging taxa from the Gondwanan landmasses (e.g., elasmarians, Australian ornithopods)(e.g., [105, 106]) and a recently expanding conception of Rhabodontomorpha (e.g., [33]). However, its relationships with these taxa, particularly rhabdodontomorphs, and dryomorphs, fluctuate in recent phylogenetic analyses, as does the composition of Rhabodonotomorpha itself, which is inconsistent across recent studies.

Norman [107] recovered a sister taxon relationship between “tenontosaurs” and rhabdodontids. Similarly, Poole [32] found *Tenontosaurus* to be closely related to rhabdodontids and recovered it and *Muttaburrasaurus* as nested within Rhabdodontomorpha. In contrast, the majority of recent analyses fail to recover the former relationship (e.g., [27, 33, 34, 60, 64, 106]). For example, Madzia et al., [34] and Bell et al., [27] recovered *Tenontosaurus* as a transitional clade diverging between Rhabdodontomorpha (incl. *Muttaburrasaurus*) and Dryomorpha, and Dieudonné et al., [33] recovered it as the sister taxon to a Rhabdodontomorpha (incl. *Muttaburrasaurus* and *Fostoria*) + Dryomorpha clade. Bell et al., [106] did not find either taxon as a member of Rhabdodontomorpha.

Our phylogenetic analyses posit a close relationship between *Iani*, *Tenontosaurus*, and rhabdodontomorphs. This relationship is supported by the following traits: a subrectangular orbit, lack of a well-defined primary ridge on the maxillary dentition, and a grooved depression on the lateral aspect of the maxillary process of the jugal (*Iani* + *Tenontosaurus* + other rhabdodontomorphs); heavily ridged dentary crowns bearing 12 or more secondary ridges on the lingual surface, a continuous convex ventral margin of the dentary, and a posttemporal foramen housed entirely in the squamosal (exclusive to *Iani* + other rhabdodontomorphs); and a subrectangular tab on the caudal margin of the squamosal appressed against the paroccipital process (*Iani* + *Tenontosaurus*)(not coded in analyses). Several of these traits were considered unambiguous synapomorphies of rhabdodontids in previous studies (e.g., [39, 108]), although the most recent diagnosis provided by Dieudonné et al., [33] relies more heavily on characters of the postcranial skeleton. The distribution of these features in our analyses follows published character assessments captured by recent matrices and our direct observations of select taxa among the global representation of ornithopods, including extensive character recoding of *Te*. *tilletti* and *Te*. *dossi* based on first-hand observation. Although we find evidence that *Iani* is most closely related to rhabdodontomorphs, we note several concerns. To date, a posttemporal foramen puncturing the body of the squamosal is documented only in *Iani* and rhabdodontids; however, the position of this foramen is not commonly described and can be difficult to assess on articulated skulls, thus it is possible it has a wider distribution. In addition, the number of ridges on the dentary crowns is arbitrarily discretized in the matrices we employ (lacking evidence for gap-coding approaches). For example, character states for the number of secondary ridges in Poole [32] and Dieudonné et al., [33] are divided between 11 and 12 (the former being one state and the latter being a different state). *Iani* possesses up to 12 secondary ridges per dentary crown and is thus aligned with rhabdodontomorphs in these studies based on the presence of only one additional ridge over other taxa. We also observe that the number of ridges varies across the tooth row of *Iani* (and therefore likely other species) and for some taxa may the number coded may not be accurately assessed (e.g., *Talenkauen* for which no number is described, but more than 11 secondary ridges may be present [105: Fig 11]). As noted by Poole [32], it is possible that similar dietary ecology resulted in convergent evolution, particularly in the dental apparatus of some early-branching ornithopods, and that these features reflect similar ecomorphology rather than shared heritage. Finally, we note that the position of *Muttaburrasaurus*, *Fostoria*, and the Vegagete ornithopod as the latest diverging rhabdodontomorphs in our analysis of the Dieudonné et al., [33] matrix creates complex character transformations and biogeographical scenarios, suggesting potential topological problems. This is mirrored by conflicting morphological and temporal patterns between *Iani* and *Tenontosaurus*.

Although *Iani* is geologically younger than *Te*. *dossi* (latest Albian ~113 Ma [62]) and *Te*. *tilletti* (124–98 Ma [109]) patterns of morphological evolution as reconstructed via our phylogenetic results do not suggest that *Iani* is a descendant (“survivor”) of an evolving “*Tenontosaurus* lineage.” Rather, *Iani* is somewhat transitional between *Tenontosaurus* and other rhabdodontomorphs in some features (e.g., number of ridges on dentary teeth, supraoccipital participating in the foramen magnum, a more reduced and tapering predentary, a steeper premaxillary body), and in other respects exhibits traits that are more plesiomorphic than either clade and not expected to vary ontogenetically (e.g., three premaxillary teeth). This complicates biogeographical scenarios leading up to the appearance of *Iani* in Cenomanian ecosystems of North America. Our problems generating confident phylogenetic relationships for *Iani* mirror those of other recent studies (e.g., [110]). Although we find evidence for a monophyletic Rhabodontomorpha that includes *Tenontosaurus* and *Iani*, we note weak and/or conflicting character support and inconsistent trait characterizations within the matrices we analyzed and, therefore, caution that standardizing interpretations of morphological evolution broadly across early-diverging ornithopods in light of new taxa is both a needed endeavor and could overturn these hypotheses.

### Rhabdodontomorph assemblage data

Quantitative and descriptive studies of rhabdodontid dietary ecology suggest these ornithischians were specialized, high-fiber herbivores that occupied dietary niches distinct from those of hadrosauromorphs and nodosaurids [111], with possible overlap among early ceratopsians [108]. However, to date, our understanding of ornithischian niche partitioning and sympatry in rhabdodontomorph-bearing assemblages is limited, stemming predominantly from the mosaic Late Cretaceous record of the European archipelago, with inconsistently resolved taxa from Australia and North America.

Throughout most of the Late Cretaceous, rhabdodontomorphs lived alongside titanosaurs and ankylosaurians on the Ibero-Armorican landmass (southern France and Iberia) (e.g., [37, 108]). However, a rapid faunal turnover event whereby rhabdodontomorphs and nodosaurs were replaced by hadrosaurids is hypothesized to have occurred in the latest Maastrichtian [108, 112, 113]. At least two sites seemingly refute this hypothesis, preserving evidence of sympatric rhabdodontomorphs and hadrosauromorphs (Vitrolles-la-Plaine, southern France, and Laño, Iberia). One is noted to be an allochthonous assemblage, potentially capturing faunal components of different ecosystems [108, 114], and the other is based on the preservation of a single hadrosauromorph tooth [113, 115]. Thus, there is some, albeit weak, evidence for an extended period of faunal mixing between rhabdodontomorphs and hadrosauromorphs in Ibero-Armorica during the terminal Cretaceous. By contrast, rhabdodontomorphs persisted alongside nodosaurids, titanosaurians, and hadrosauromorphs in Transylvania (e.g., Haţeg Basin, Romania) through to the terminal Maastrichtian [61, 116], indicating that a complex trophic structure of ornithischians including rhabdodontomorphs and later-diverging ornithopods characterized a large region of the European Archipelago in the Maastrichtian [113].

Although rhabdodontids appear to have been endemic to Europe, the Australian ornithopods *Muttaburrasaurus* and *Fostoria* have been variably recovered as members of the broader clade Rhabdodontomorpha (e.g., [23, 32, 33, 37 but see 106]). *Muttaburrasauru*s *langdoni* stems from the Makunda Formation (and *Muttaburrasauru*s sp. from the Allaru Mudstone) [27, 117], for which the dinosaurian record is poor. However, the ornithischian record of the Griman Creek Formation is more complete and captures a diverse assemblage including four ornithopods (*Fostoria*, *Weewarrasaurus*, and indeterminate taxa), ankylosaurians, and sauropods [27, 106, 117]. To date, no later-diverging iguanodontians are known to have cohabited with potential rhabdodontomorphs in Australian ecosystems.

The presence of rhabdodontomorphs in North America is a relatively recent hypothesis stemming from newly recovered phylogenetic relationships of the early-diverging ornithopod genus *Tenontosaurus*—one of the most common Aptian-Albian macrovertebrates described from multiple formations spanning ~25 million years of evolutionary time [60, 62, 95, 109, 118]. Specimens referred to *Tenontosaurus* are currently divided into only two species, *Te*. *tilletti* from the Cloverly and Antlers formations and *Te*. *dossi*, from the Twin Mountains Formation (the latter overlaps in age with specimens referred to *Te*. *tilletti* at our current level of temporal resolution) [62, 109]. Although Thomas [60] did not find evidence for dividing the current hypodigm of *Te*. *tilletti* into multiple species, we find it unlikely only a single taxon is represented amongst all the specimens and occurrences referred to this species and suggest future work accounting for ontogeny will provide additional resolution. As a case in point, a taxon morphologically similar to *Tenontosaurus* was initially recognized in the Mussentuchit Member of the Cedar Mountain Formation from isolated teeth (cf. *Tenontosaurus* [12, 18, 119]. Discovery of the partial skeleton NCSM 29373, allows us to herein refine this identification to the new genus and species, *Iani smithi*.

**Fig 21:**
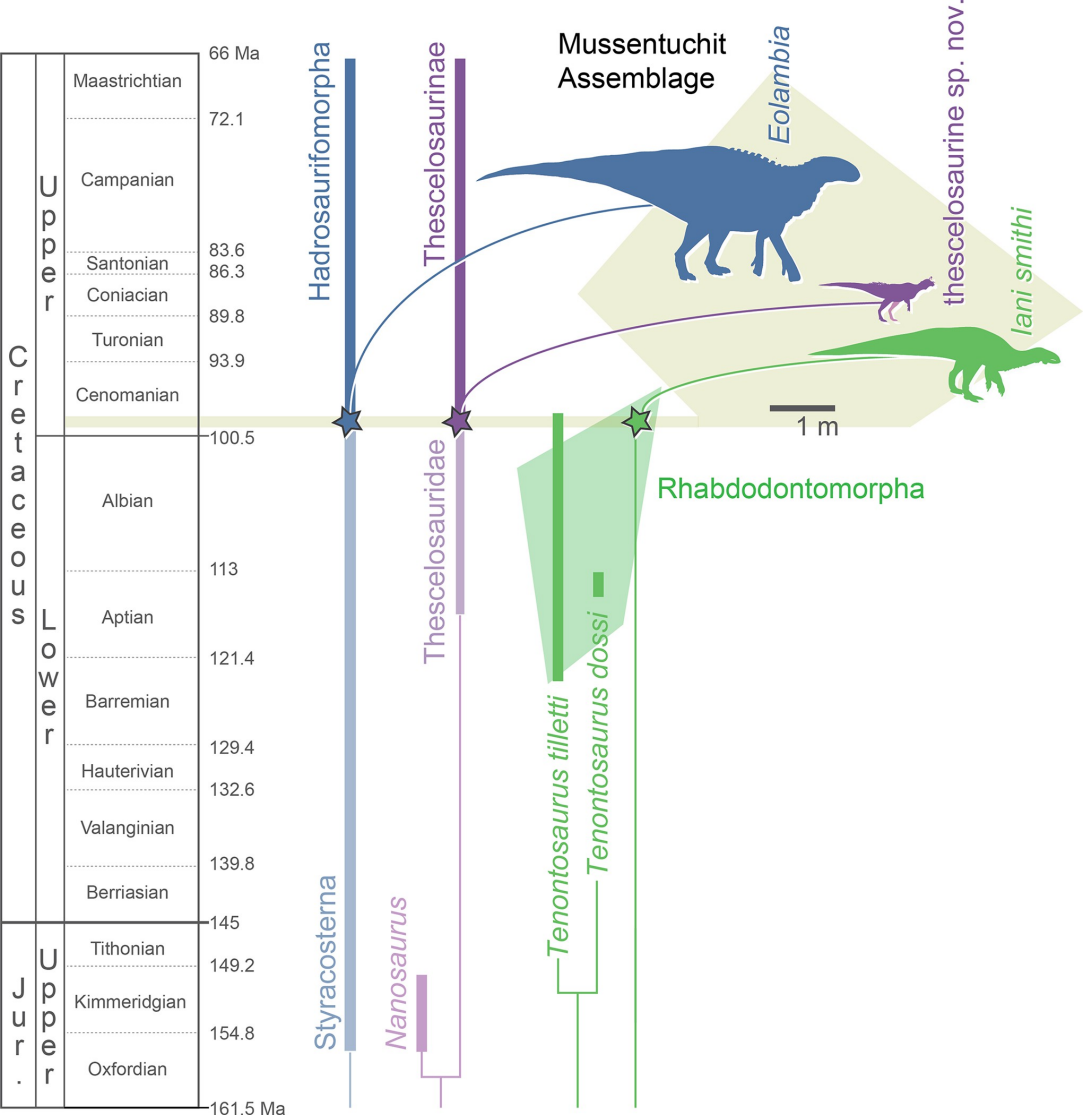
Chronostratigraphic and assemblage data of non-ceratopsian neornithischians of the Mussentuchit member. (A) stratigraphic ranges of North American members of select clades, (B) silhouettes of non-ceratopsian neornithischians of the Mussentuchit assemblage showing relative taxon size. Stratigraphic range of Thescelosauridae based on *Zephyrosaurus* (first occurrence)(middle of the Little Sheep Member, Cloverly Formation [122], mean age ~116.5 Myr [109]) and *Thescelosaurus* (last occurrence)(Hell Creek Formation, youngest age 66 Ma [123]). Stratigraphic range of Styracosterna based on *Uteodon* (first occurrence)(Brushy Basin Member, Morrison Formation, mean age ~151 Ma [124]) and *Edmontosaurus* (last occurrence)(Hell Creek Formation, youngest age 66 Ma [123]) and the nested clade Hadrosauromorpha based on first *Eolambia* (first occurrence)(Mussentuchit Member, Cedar Mountain Formation, oldest age ~99.5 Ma [59]) and *Edmontosaurus* (last occurrence)(Hell Creek Formation, youngest age 66 Ma [123]). Phylogenetic topology and clade composition follow [125, 126]. Taxonomic definitions follow Madzia et al., [28].

Spatiotemporal data suggests that Thescelosauridae, Hadrosauriformes, and Rhabdodontomorpha were present in late Early Cretaceous ecosystems of North America. For example, thescelosaurids and hadrosauriforms inhabited the continent at minimum from the Aptian through Maastrichtian (e.g., *Zephyrosaurus* to *Thescelosaurus*; *Eolambia/Protohadros* to *Edmontosaurus*) and *Tenontosaurus* is known from multiple formations of Aptian-Albian age [62, 95] (Fig 21). The discovery of *Iani* in the Mussentuchit Member, when combined with an undescribed new species of thescelosaurine [91, 120, 121], the hadrosauriform *Eolambia caroljonesa*, and fragmentary records of ceratopsians and ankylosaurians in the same strata, definitively documents that at least five clades of neornithischians cohabitated within Cenomanian ecosystems of western North America and also survived across the Early–Late Cretaceous boundary in the region.

### Paleoenvironmental associations

Forster [127] proposed an increasing abundance of *Tenontosaurus tilletti* between the Little Sheep Mudstone Member and the relatively wetter, more coastal Himes Member of the Cloverly Formation, and used these data to suggest that the taxon was more abundant in settings proximal to the shoreline of the Western Interior Seaway. However, as noted by Forster [127], sampling and preservational biases could also explain this pattern, since the Himes Member preserves more macrovertebrate fossils [118], whereas more microvertebrate bonebeds are described from the Little Sheep Mudstone [109, 122]. With the current data at hand, preservation bias versus paleoenvironmental signals cannot be parsed.

Issues of relative abundance notwithstanding, *Te*. *tilletti* is known to have inhabited coastal settings including the Antlers Formation, which represents fluvial, deltaic, and strandplain environments [128, 129]. Likewise, specimens of *Te*. *dossi* were recovered from brackish water estuarine environments of the Twin Mountains Formation, overlain by deltaplain channels [62]. In a similar vein, the holotype skeleton of *Iani* derives from paralic sediments of the Mussentuchit Member, Cedar Mountain Formation, capturing life on an evolving deltaplain [130]. The lower Mussentuchit Member represents a wet, humid environment inundated by a high base level of brackish groundwater and minor tidal influences [130]. This is comparable with paleoenvironmental interpretations of *Tenontosaurus*-bearing strata of the Twin Mountains and Antlers formations. Thus, the presence of *Iani* in the Mussentuchit Member provides additional evidence for paleoenvironmental links between North American rhabdodontomorphs and coastal environments. However, it should be noted that there is also evidence for closely related taxa in the more arid Wayan-Vaughn Assemblage and underlying Ruby Ranch Formation in the form of isolated teeth and fragmentary remains [12, 131]; therefore, North American “tenontosaurs” were clearly not restricted to such habitats. Regardless, further research into the paleoenvironmental distribution of North American rhabdodontomorphs, particularly in assemblages with cohabiting hadrosauroids, may yield important insight into their distinct dietary ecology and habitat preferences.

To date, few macrovertebrate fossils are known from Turonian–Santonian Formations, thus the timing of rhabdodontoid extirpation in the Western Interior Basin is presently indeterminate. Given the potential for habitat preferences, reexamination of existing microvertebrate bonebed collections from late mid-Cretaceous coastal settings of the Western Interior Basin (e.g., the Straight Cliffs of southern Utah [132]) may ultimately add key information constraining the timing of this event.
